# Estimating the costs of air pollution to the National Health Service and social care: An assessment and forecast up to 2035

**DOI:** 10.1371/journal.pmed.1002602

**Published:** 2018-07-10

**Authors:** Laura Pimpin, Lise Retat, Daniela Fecht, Laure de Preux, Franco Sassi, John Gulliver, Annalisa Belloni, Brian Ferguson, Emily Corbould, Abbygail Jaccard, Laura Webber

**Affiliations:** 1 UK Health Forum, London, United Kingdom; 2 Small Area Health Statistics Unit, MRC-PHE Centre for Environment and Health, School of Public Health, Imperial College London, London, United Kingdom; 3 Centre for Health Economics & Policy Innovation, Imperial College Business School, London, United Kingdom; 4 Public Health England, London, United Kingdom; Edinburgh University, UNITED KINGDOM

## Abstract

**Background:**

Air pollution damages health by promoting the onset of some non-communicable diseases (NCDs), putting additional strain on the National Health Service (NHS) and social care. This study quantifies the total health and related NHS and social care cost burden due to fine particulate matter (PM_2.5_) and nitrogen dioxide (NO_2_) in England.

**Method and findings:**

Air pollutant concentration surfaces from land use regression models and cost data from hospital admissions data and a literature review were fed into a microsimulation model, that was run from 2015 to 2035. Different scenarios were modelled: (1) baseline ‘no change’ scenario; (2) individuals’ pollutant exposure is reduced to natural (non-anthropogenic) levels to compute the disease cases attributable to PM_2.5_ and NO_2_; (3) PM_2.5_ and NO_2_ concentrations reduced by 1 μg/m^3^; and (4) NO_2_ annual European Union limit values reached (40 μg/m^3^). For the 18 years after baseline, the total cumulative cost to the NHS and social care is estimated at £5.37 billion for PM_2.5_ and NO_2_ combined, rising to £18.57 billion when costs for diseases for which there is less robust evidence are included. These costs are due to the cumulative incidence of air-pollution-related NCDs, such as 348,878 coronary heart disease cases estimated to be attributable to PM_2.5_ and 573,363 diabetes cases estimated to be attributable to NO_2_ by 2035. Findings from modelling studies are limited by the conceptual model, assumptions, and the availability and quality of input data.

**Conclusions:**

Approximately 2.5 million cases of NCDs attributable to air pollution are predicted by 2035 if PM_2.5_ and NO_2_ stay at current levels, making air pollution an important public health priority. In future work, the modelling framework should be updated to include multi-pollutant exposure–response functions, as well as to disaggregate results by socioeconomic status.

## Abstract 2

Using microsimulation, Laura Webber and colleagues project the health and social care cost burden of fine particulate matter (PM2.5) and nitrogen dioxide (NO2) in England.

## Author summary

**Why was this study done?:**

- Air pollution is the leading environmental cause of early death—contributing to the equivalent of 5% of all deaths globally and an estimated 40,000 premature deaths each year in the UK. Air pollution is also responsible for a substantial amount of morbidity, causing short- and long-term health effects, as well as being a significant contributor to health inequalities.
- Tackling air pollution will improve both the environment and public health. In the new National Institute for Health and Care Excellence/Public Health England air pollution guidance, local authorities are urged to consider a range of effective interventions that promote low or zero emission areas.
- Knowing the local impact of air pollution on health and related healthcare costs over time is important for future policy and resource planning and to target mitigation measures and public health campaigns.

**What did the researchers do and find?:**

- This study used a dynamic microsimulation model to predict the future health and economic impact of the air pollutants PM_2.5_ and NO_2_ to the year 2035. This microsimulation examined data on air pollution exposure by age and sex, making use of disease and population data collected from the literature and from publicly available databases. The microsimulation method is an advanced method for modelling chronic diseases because of its capacity to simulate entire populations at an individual level over a lifetime.
- Between 2017 and 2025, the total cost to the NHS and social care of air pollution for diseases for which there is more robust evidence for an association is estimated to be £5.56 billion, corresponding to 1.15 million new cases of disease.

**What do these findings mean?:**

- This is the first time to our knowledge that the healthcare costs of air pollution have been estimated in England. The microsimulation tool provides estimates of the prevalence of disease and related NHS and social care costs due to air pollution. These estimates are important and useful statistics that highlight the burden of air pollution to the healthcare system and quantify the health and related NHS and social care cost savings under different scenarios compared to a ‘do nothing’ scenario.
- The impacts of air pollution and the action required to address them are highly relevant to local government priorities: health, housing, transport, education, local economies, greenspace, and quality of life. Local authorities have long had specific legal air quality powers to tackle air pollution locally where there is evidence from either the local or national assessment regimes that it exceeds legal limits. Alongside these specific obligations, strategic decisions on transport, planning, and public health taken by local governments all contribute to the quality of the air that people breathe in local communities.
- The UK Government will publish a final clean air strategy by the end of the year. This strategy will set out the range of actions the government will take in the coming years to tackle emissions of 5 key pollutants from a wide range of sources. There will be a need to continually improve our understanding of the important health, environmental, and economic consequences of air pollution in order to deliver an ambitious programme of actions.

## Introduction

Air pollution is a major public health concern with a high burden of disease. Exposure to fine particulate matter (PM_2.5_) and nitrogen dioxide (NO_2_) increases the risk of non-communicable diseases (NCDs) such as cardiovascular disease, respiratory disease, and lung cancer [1–3], as well as exacerbating existing conditions such as asthma and chronic obstructive pulmonary disease (COPD). These chronic conditions are expensive to treat and put unnecessary strain on an already overworked healthcare system. Little is known about the extent of the attributable cost of these diseases to air pollution, now and into the future. Understanding the extent and magnitude of air pollution’s impact on long-term health is important for future National Health Service (NHS) budget allocation, priority setting, and policy planning.

In light of this, and in response to the UK Parliament Environmental Audit Committee’s conclusion [4] that ‘the Government must…urgently quantify the impact on morbidity and the cost to the NHS of poor air quality’, this study developed a modelling framework to monetise the present and future morbidity attributable to long-term exposure to PM_2.5_ and NO_2_ from outdoor sources in terms of primary care visits, prescriptions, secondary care (inpatient and outpatient) visits, and social care. Social care is defined here as state-funded out-of-hospital care provided in the community and at a patient’s home with the primary aim of reducing or managing the deterioration in health status for people with a degree of long-term dependency, helping them with activities of daily living, and assisting them to live independently. In estimating the health and economic burden of air pollution, we build an economic case for investing in preventative interventions.

Using a microsimulation model, the population of England was simulated, and the number of new air-pollution-related disease cases and resultant costs to the NHS and social care from exposure to PM_2.5_ and NO_2_ were estimated from 2017 to 2035, as a total and separately for various conditions, while taking into account overlaps between the 2 pollutants.

## Methods

### The projection and microsimulation model

We used a microsimulation model to produce longitudinal projections of air-pollution-attributable NCDs and related NHS and social care costs from 2017 to 2035. The microsimulation method is a rigorous method for modelling and projecting the long-term health impacts of chronic diseases into the future. The methods applied have been described in detail before [5,6]. Study-specific adaptations to the model are described in depth in S1 Text. We created a virtual population of 50 million individuals in the microsimulation. Each individual was exposed to PM_2.5_ and NO_2_ according to 2015 exposure data by age and sex (described in detail below). The population was distributed based on 2015 demographic characteristics from the Office for National Statistics (ONS) (S2 Text) [7]. Fifty million individuals were modelled to enhance power and detection of low-incidence diseases. All outputs were scaled to the ONS 2015 mid-year estimates for England. The microsimulation started in 2015 since the most recent population statistics were available from that year. However, the future scenarios were implemented from 2017, since this was the start year of the study. Therefore, cumulative figures were calculated from 2017 onwards. Population characteristics varied over time because of aging populations, births, and deaths. In the microsimulation, PM_2.5_ and NO_2_ were treated as individual risk factors, which were constant over time.

Asthma, COPD, coronary heart disease, stroke, type 2 diabetes, and lung cancer were included within the microsimulation based on estimates of associations between exposure to the pollutants and risk of developing the diseases, which were obtained from meta-analyses of prospective cohort studies. Low birth weight and dementia were also included, although the evidence is less well established for these conditions and, for dementia, came from a single prospective cohort study. No costs were available for low birth weight, so only the epidemiological model outputs for this condition are reported (see S3 Text). At birth, each individual in the microsimulation was probabilistically assigned an age- and sex-specific PM_2.5_ and NO_2_ exposure (i.e., their risk factor). Then, each year, exposure levels were derived using the assumption that the exposure percentile for an individual stays constant over time. The exposure trends were assumed to be static over time. Each year, a simulated individual was at risk of developing a new air-pollution-related NCD, dying from an existing NCD or from other non-air-pollution-related causes, surviving with an existing NCD, or remaining in a disease-free state.

The microsimulation also contained an economic module that produced Markov-type simulations of long-term health benefits and healthcare costs, including primary and secondary care, medication, and social care costs. The initial cost inputs were adjusted and inflated to 2015 values with a 1.5% inflation rate. The outputs of the simulation were discounted at a 1.5% rate [8].

The confidence limits that accompany the sets of output data represent the accuracy of the microsimulation as opposed to the confidence of the input data itself. Confidence intervals around the input data were not available.

### Exposure data

We used outdoor air pollution concentration surfaces from land use regression (LUR) models covering England on a 100-m grid for PM_2.5_ [9] and 200-m grid for NO_2_ [10], the highest resolution map available for each pollutant. These models were developed to support epidemiological studies and take into account both local and long-range transport sources. They have been extensively validated against measured concentrations from the Automatic Urban and Rural Network, which contains over 100 spatially distributed continuous measurement sites [9].

We adopted a method described in Gulliver et al. for extrapolation of the air pollution surfaces (from 2010 for PM_2.5_ and 2009 for NO_2_) to 2015 [10]. The method compares the difference in rural background concentrations at concomitant sites from the source year (i.e., the year each air pollution surface was developed) and the target year (i.e., 2015) for exposure estimation. These absolute differences are then applied to extrapolate (i.e., reduce) modelled concentrations from the source year to the target year (see S2 Text).

Postcode centroids (*x*,*y* locations) represent the central address of all addresses sharing the same postcode (an average of 18 households share a postcode in England). We intersected all postcode centroids, from the collection of postcode headcount information as part of the 2011 census in England (*n* = 1,227,431), with the air pollution surface data to obtain PM_2.5_ and NO_2_ estimates for each postcode. We applied a difference of −1.2 μg/m^3^ to all PM_2.5_ exposure estimates from the 2010 model and −2.3 μg/m^3^ to all NO_2_ exposure estimates from the 2009 model to forward extrapolate them to the 2015 context (see S2 Text). We estimated pollutant exposures by 5-year age group and sex, by assigning each postcode to the census output area age-sex structure from the ONS mid-year population estimates for 2015 (the most recently available at the time of this study). We then derived exposure distributions by 5-year age group and sex for each exposure tertile category specific to England.

We used information on the contribution to total PM_2.5_ from dust and sea salt from the Atmospheric Composition Analysis Group at Dalhousie University, Canada (spatial scale approximately 620 m × 620 m) [11], to distinguish between the anthropogenic contribution (e.g., from combustion) and the non-anthropogenic contribution (e.g., wind-blown desert and mineral dust and sea salt spray) [12]. This enabled us to compare each individual’s exposure at non-anthropogenic, i.e., natural, levels to a ‘no change’ scenario where exposure stays at current levels, in order to compute the total attributable number of new diseases caused by PM_2.5_. We did not include information on non-anthropogenic NO_2_ estimates as these data were not readily available for this study and most NO_2_ is from anthropogenic sources, with a very low natural, non-anthropogenic source of air pollution that might vary over short-term periods [13].

### Disease data

We collected data on incidence, prevalence, mortality, survival, and exposure–response relationships for long-term air-pollution-related diseases from the published literature and national databases. See Table 1 and S2 Text for a summary of the references for the sources of epidemiological data. Table 2 presents the exposure–response relationships identified for each pollutant. All of these diseases were modelled as chronic, lifelong terminal diseases with no remission possible, with the exception of type 2 diabetes and low birth weight, which were considered non-terminal. Where epidemiological parameters were not available, for instance survival rates for stroke, we computed the parameters from other sources of data using a method adapted from the World Health Organization equations from the DISMOD II tool.

**Table 1:** Summary of disease statistics used in the model.

Disease	Incidence	Prevalence	Mortality	Survival
**Asthma**	BLF asthma statistics [14]	BLF asthma statistics [14]	ONS death registration summary statistics, England and Wales, 2015 [15]	Computed from prevalence and mortality
**COPD**	Computed from prevalence and mortality	PHE modelled estimates, 2008 [16]	ONS death registration summary statistics, England and Wales, 2015 [15]	Computed from prevalence and mortality
**CHD**	Smolina et al. corrected data on incidence and mortality in 2013 [17]	BHF cardiovascular disease statistics, 2014 [18]	ONS death registration summary statistics, England and Wales, 2015 [15]	Computed from prevalence and mortality
**Diabetes**	Personal communication with Dr Craig Curry from Cardiff University	National Diabetes Audit 2015–2016 [19]	Non-terminal	Non-terminal
**Stroke**	BHF stroke statistics, 2009 [20]	BHF cardiovascular disease statistics, 2014 [18]	ONS death registration summary statistics, England and Wales, 2015 [15]	Computed from prevalence and mortality
**Dementia**	Computed from prevalence and mortality	Alzheimer’s Society Dementia UK report, 2014 [21]	ONS death registration summary statistics, England and Wales, 2015 [15]	Computed from prevalence and mortality
**Low birth weight**	ONS birth characteristics, 2015	Considered equivalent to incidence	Non-terminal	Non-terminal
**Lung cancer**	CRUK, 2012–2014 [22]	Not required in model as model uses incidence	CRUK, 2012–2014 [22]	1, 5 year: ONS, 2010–2014 [23]; 10 year: ONS, 2008–2012 [24]

BHF, British Heart Foundation; BLF, British Lung Foundation; CHD, coronary heart disease; COPD, and chronic obstructive pulmonary disease; CRUK, Cancer Research UK; ONS, Office for National Statistics; PHE, Public Health England.

**Table 2:** Exposure–response relationships identified for each disease by pollutant.

Outcome	PM_2.5_ per 10 μg/m^3^ (95% CI)	NO_2_ per 10 μg/m^3^* (95% CI)
**Respiratory outcomes**		
Asthma (children ≤6 years)	Not available	OR 1.08 (1.01 to 1.12)
Asthma (children >6 years)	OR 1.48 (1.22 to 1.97)	OR 1.03 (1.00 to 1.06)
Asthma (adults)	Not available	OR 1.04 (1.00 to 1.08)
COPD/chronic bronchitis (adults)	OR 1.49 (1.03 to 2.14)	Not available
**Cardiovascular outcomes**		
CHD (adults)	HR 1.41 (1.00 to 2.01)	Not available
Stroke (adults)	HR 1.13 (1.04 to 1.23)	Not available
Diabetes (adults)	RR 1.10 (1.02 to 1.18)	RR 1.05 (1.02 to 1.07)
**Cancer and other outcomes**		
Lung cancer (adults)	RR 1.09 (1.04 to 1.14)	RR 1.02 (1.00 to 1.03)
Dementia (adults)	Not available	HR 1.01 (1.01 to 1.03)
Low birth weight^†^	OR 1.39 (1.12 to 1.77)	OR 1.04 (1.00 to 1.07)

While confidence intervals are presented here, the microsimulation input only includes the central point estimates. Figures highlighted in grey represent evidence of a strong causal link.

*Reduced by 60% from the published values as per UK Committee on the Medical Effects of Air Pollution recommendations.

^†^Modelled as associated with a woman who gives birth, see S2 Text.

CHD, coronary heart disease; COPD, and chronic obstructive pulmonary disease; HR, hazard ratio; OR, odds ratio; RR, relative risk.

While some exposure–disease relationships have been reviewed and quantified by the UK Committee on the Medical Effects of Air Pollution (COMEAP), others have been extracted from the peer-reviewed literature [2,25], and serve as the current best estimate as to what the true exposure–response relationship is. Therefore, the strength of the association with air pollution varied for each disease. Specifically, the evidence linking air pollution to dementia has recently emerged; therefore, we interpret these findings with more caution. Those diseases with robust evidence of a causal relationship are highlighted in grey in Table 2. Those diseases where evidence is just associative and/or where evidence is emerging are not highlighted.

Exposure–response estimates for NO_2_ were adjusted and reduced by 60% to take account of overlaps in risks with other pollutants including PM_2.5_, as advised and described by COMEAP [26]. See Table 2 for the exposure–response relationships used, and S2 Text for the sources and methods for their selection and adjustment.

### Cost data

Four different categories of costs were included: primary care, prescription, secondary care (inpatient and outpatient), and social care costs.

In order to derive the NHS costs associated with each health condition, we used 2 different sources: published literature and the Hospital Episode Statistics (HES) dataset. Inpatient costs for each disease were estimated using HES, which captures actual healthcare utilisation in NHS hospitals. We adopted a conservative approach and selected only the HES episodes in 2015 for which the main diagnosis corresponded to one of the ICD-10 codes of interest. ICD-10 codes selected for the analysis were as follows: I20–I25 (coronary heart disease); E10, E11, O24.4 (type 2 diabetes); J40–J44 (COPD); C34 (lung cancer); and I60–I63 (stroke). Dementia was not analysed using HES as HES does not include specialised institutions, and we therefore relied entirely on the literature. We then matched each episode based on its NHS Healthcare Resource Group (HRG) to the tariff at which it was reimbursed, and used the NHS Market Forces Factor to account for regional differences in the cost of land, capital, and labour. We estimated an average episode cost per health condition, and multiplied the number of yearly diagnosed cases by the estimated average cost to account for errors in HRG reporting that did not allow a direct match to a tariff.

When healthcare utilisation data were not available, we relied on literature providing estimates of health and social care costs associated with the conditions of interest. We conducted a literature review and selected studies based on robust and transparent methods. The relevant literature was identified using PubMed and MeSH terms, or Google when no indexed publications reported the cost data of interest. S3 Text describes the costs extracted from the literature in more detail. When costs were based on UK-wide or Scottish figures, costs were adjusted for the England population.

There was a large variation in the definition of social care in the literature. This is due to the lack of available data and the need to rely on some proxy measures, the absence of a clear definition of what counts as social care, and a lack of clear distinction between privately and publicly funded care. Social care costs usually capture costs related to informal care that is funded publicly.

The microsimulation model uses cost per case or cost per death to calculate the total health and social care costs incurred due to the prevalence of disease.

Table 3 provides the cost per case or cost per death for each disease used in the microsimulation model by type of care. These costs were adjusted for inflation in 2015 using the Hospital and Community Health Services (HCHS) inflation index to match the year of population data in the model (HCHS pay and price inflation is a weighted average of 2 separate inflation indices, the Pay Cost Index and the Health Service Cost Index). These inflated total costs were then divided by the study country’s disease prevalence in 2015 in order to have an average prevalence cost per case.

**Table 3:** Cost based on total prevalence of each disease, expressed in British pounds per prevalence case per year.

Disease	Cost (British pounds per case) by type of care			
	Primary care	Secondary care	Medication	Social care
Asthma	21.28	27.02	87.57	0.50*
Chronic obstructive pulmonary disease	400.43	587.48	126.79	85.30*
Coronary heart disease	71.57	1,460.46	818.60	109.70
Stroke	36.45	722.84	504.10	76.05*
Diabetes	375.00	536.75	276.88	601.56*
Lung cancer	51.73*	466.63*	35.10*	89.38*
Dementia	430.62	197.24	310.24	6,174.47

*Indicates cost per death for palliative care.

### Scenarios

Different scenarios were run within the microsimulation:

1. Baseline ‘no change’ scenario, where PM_2.5_ and NO_2_ exposure stays at 2015 levels.
2. Each individual’s PM_2.5_ and NO_2_ exposure is reduced to natural (non-anthropogenic sources) levels and compared to the no-change scenario, where exposure stays at current levels. This enables the total attributable number of new diseases caused by PM_2.5_ or NO_2_ pollution to be quantified. Natural (non-anthropogenic) sources of PM_2.5_ arise from natural sources, e.g., mineral dust and sea-salt.
3. Each individual’s PM_2.5_ and NO_2_ exposure is reduced by 1 μg/m^3^ in 2017 only and is compared to the no-change scenario, where exposure stays at current levels.
4. Each individual’s NO_2_ exposure is reduced to meet the EU limit value for NO_2_ (annual average of 40 μg/m^3^) and is compared to the no-change scenario, where exposure stays at current levels. This scenario was not run for PM_2.5_ because EU limit values (annual average of 25 μg/m^3^) have been met in England.

## Results

### Disease cases and costs attributable to PM_2.5_ and NO_2_

**Fig 1:**
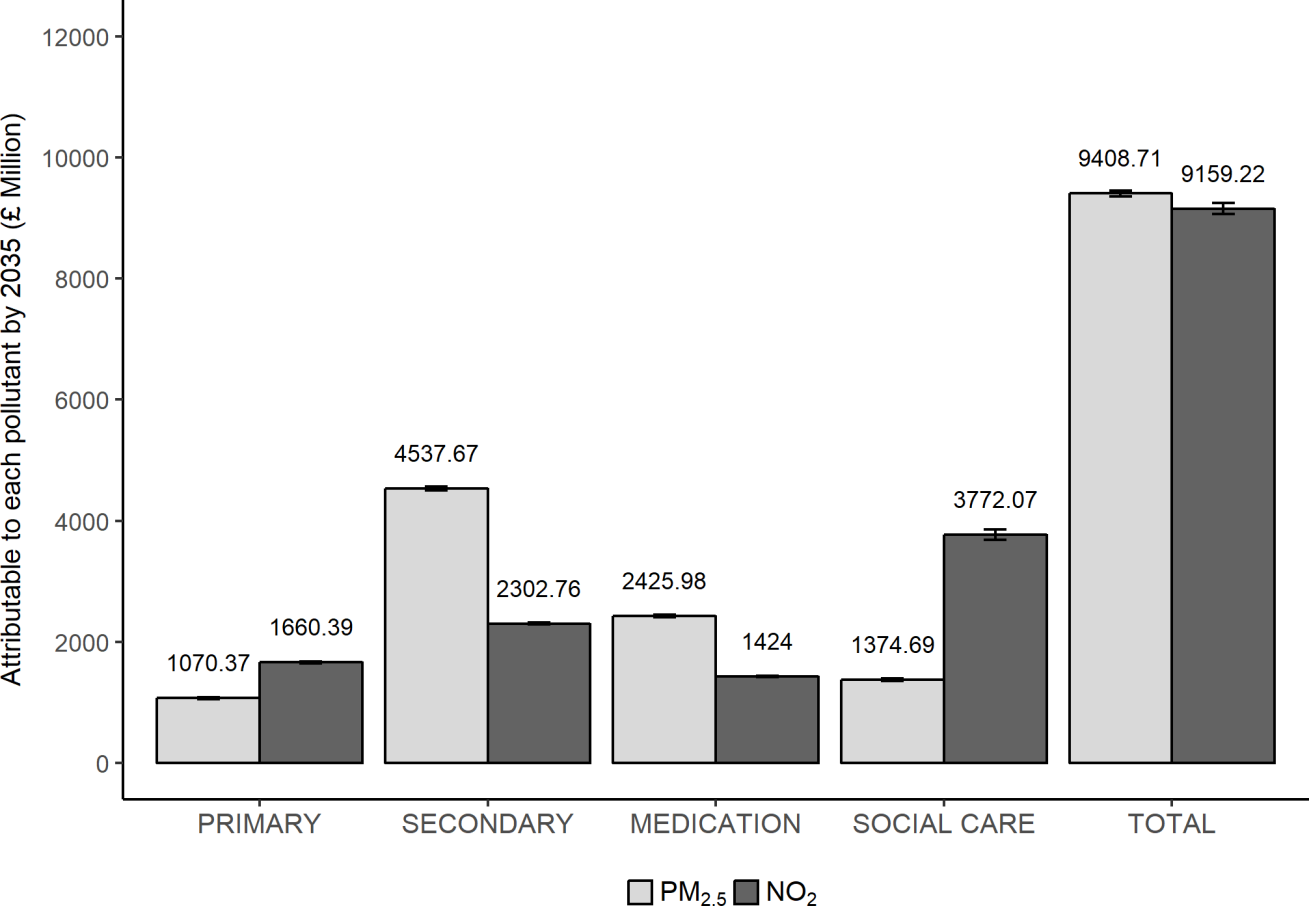
Costs attributable to PM_2.5_ and NO_2_ by 2035 for each cost parameter. The confidence limits that accompany the sets of output data represent the accuracy of the microsimulation as opposed to the confidence of the input data itself. Confidence intervals around the input data were not available.

Table 4 and Fig 1 provide a summary of the total disease cases and costs attributable to PM_2.5_ and NO_2_. These costs were based on both the total costs of the stronger and weaker evidence combined. In 2017, the attributable cases of disease due to PM_2.5_ were estimated to be 114 (95% CI 110 to 118) per 100,000, which scales to 63,430 (95% CI 61,276 to 65,584) cases in the total population of England, at a cost of £71.10 (95% CI 67.9 to 84.3) million to the NHS and social care. Cumulatively, by 2025 and 2035, we predicted a total of 607,917 (95% CI 601,661 to 614,173) and 1,327,424 (95% CI 1,317,505 to 1,337,343) disease cases attributable to PM_2.5_, costing the NHS and social care £2.81 (95% CI 2.79 to 2.84) billion and £9.41 (95% CI 9.36 to 9.45) billion, respectively. The largest contribution of cost came from secondary care: £36.8 (95% CI 31.0 to 42.7) million in 2017, £1.37 (95% CI 1.35 to 1.39) billion by 2025, and £4.54 (95% CI 4.51 to 4.57) billion by 2035.

**Table 4:** Total disease cases and costs attributable to PM_2.5_ and NO_2_.

Parameter	PM_2.5_			NO_2_		
	2017 (95% CI)	2017–2025 (95% CI)	2017–2035 (95% CI)	2017 (95% CI)	2017–2025 (95% CI)	2017–2035 (95% CI)
**Attributable incident cases per 100,000**	114 (110 to 118)	1,062 (1,050 to 1,074)	2,248 (2,231 to 2,265)	109 (104 to 114)	943 (928 to 958)	1,933 (1,911 to 1,955)
**Attributable incident cases in England**	63,430 (61,276 to 65,584)	607,917 (601,661 to 614,173)	1,327,424 (1,317,505 to 1,337,343)	60,648 (58,099 to 63,197)	539,527 (531,657 to 547,397)	1,140,018 (1,128,218 to 1,151,818)
**Attributable NHS and social care costs in England (millions of British pounds)**	76.10 (67.86 to 84.34)	2,814.79 (2,787.51 to 2,842.07)	9,408.71 (9,363.33 to 9,454.08)	81.06 (64.75 to 97.37)	2,749.91 (2,695.16 to 2,804.66)	9,159.22 (9,066.51 to 9,251.93)
Primary care costs	10.45 (7.86 to 13.04)	332.02 (323.43 to 340.61)	1,070.37 (1,056.10 to 1,084.64)	12.61 (9.91 to 15.31)	469.87 (460.92 to 478.82)	1,660.39 (1,645.51 to 1,675.27)
Secondary care costs	36.83 (30.99 to 42.67)	1,368.70 (1,349.36 to 1,388.04)	4,537.67 (4,505.49 to 4,569.85)	17.09 (13.47 to 20.71)	642.75 (630.79 to 654.71)	2,302.76 (2,282.94 to 2,322.58)
Medication costs	18.81 (15.52 to 22.10)	718.53 (707.65 to 729.41)	2,425.98 (2,407.87 to 2,444.09)	10.95 (8.86 to 13.04)	399.80 (392.88 to 406.72)	1,424.00 (1,412.52 to 1,435.48)
Social care costs	10.01 (5.99 to 14.03)	395.54 (382.19 to 408.89)	1,374.69 (1,352.51 to 1,396.87)	40.41 (24.88 to 55.94)	1,237.49 (1,185.27 to 1,289.71)	3,772.07 (3,683.48 to 3,860.66)

Scaling to the population of England may lead to rounding discrepancies between cases and cases avoided.

NHS, National Health Service.

In 2017, the attributable cases of disease due to NO_2_ were estimated to be 109 (95% CI 104 to 114) per 100,000, which scales to 60,648 (95% CI 58,099 to 63,197) cases in England, at a cost of £81.06 (95% CI 64.75 to 97.37) million to the NHS and social care. Cumulatively, by 2025 and 2035, we predicted a total of 539,527 (95% CI 531,657 to 547,397) and 1,140,018 (95% CI 1,128,218 to 1,151,818) disease cases attributable to air pollution, costing the NHS and social care £2.75 (95% CI 2.70 to 2.80) billion and £9.16 (95% CI 9.07 to 9.25) billion, respectively. The largest contributor to the costs came from social care, with £1.24 (95% CI 1.19 to 1.29) billion and £3.77 (95% CI 3.68 to 3.86) billion attributable to NO_2_ by 2025 and 2035, respectively. Diseases and costs attributable to PM_2.5_ and NO_2_ by disease and cost parameter are presented in S4 Text.

### Disease cases and costs avoided due to each scenario

Table 5 presents the cumulative incidence of cases and cumulative costs avoided due to a 1-year 1-μg/m^3^ reduction in each pollutant relative to the no-change baseline, and the disease cases and costs avoided if EU limit values were met for NO_2_ relative to baseline. Reducing PM_2.5_ by 1 μg/m^3^ was estimated to reduce the air-pollution-related disease burden by 86,483 (95% CI 80,227 to 92,739) cases by 2025, and 188,415 (95% CI 178,496 to 198,334) cases by 2035. Reducing NO_2_ by 1 μg/m^3^ was estimated to reduce the air-pollution-related disease burden by 17,173 (95% CI 9,303 to 19,866) cases by 2025, and 33,589 (95% CI 21,789 to 36,435) cases by 2035. These 1-μg/m^3^ reductions in population exposure to PM_2.5_ and NO_2_ would result in £1.42 (95% CI 1.38 to 1.47) billion and £353.3 (95% CI 260.2 to 446.5) million avoided, respectively, in NHS and social care costs by 2035. If England met the EU limit values for NO_2_, it was estimated that 96,786 (95% CI 88,916 to 104,656) new cases of NO_2_-related diseases could be avoided by 2025, and 225,465 (95% CI 213,665 to 237,265) by 2035, resulting in £1.69 (95% CI 1.60 to 1.79) billion in health and social care costs avoided by 2035.

**Table 5:** Summary of cumulative incidence of cases avoided and cumulative costs avoided in England for each scenario relative to baseline for PM_2.5_ and NO_2_, by period.

Scenario	Parameter	PM_2.5_			NO_2_		
		2017 (95% CI)	2017–2025 (95% CI)	2017–2035 (95% CI)	2017 (95% CI)	2017–2025 (95% CI)	2017–2035 (95% CI)
**Baseline**	**Cumulative incidence of cases**	644,873 (643,114 to 646,632)	6,119,754 (6,114,646 to 6,124,862)	13,654,072 (13,645,973 to 13,662,171)	661,566 (659,721 to 663,411)	6,215,963 (6,210,268 to 6,221,658)	13,855,589 (13,847,049 to 13,864,129)
**1-**μ**g/m**^**3**^ **reduction in PM**_**2.5**_ **and NO**_**2**_	**Cumulative incidence of cases**	636,526 (634,767 to 638,285)	6,033,265 (6,028,157 to 6,038,373)	13,465,656 (13,457,557 to 13,473,755)	659,896 (658,051 to 661,741)	6,198,788 (6,193,093 to 6,204,483)	13,822,000 (13,813,460 to 13,830,540)
	**Cumulative incidence of cases avoided (relative to baseline)**	8,345 (6,191 to 10,499)	86,483 (80,227 to 92,739)	188,415 (178,496 to 198,334)	1,669 (−880 to 4,218)	17,173 (9,303 to 19,866)	33,589 (21,789 to 36,435)
	Total cost avoided relative to baseline (millions of British pounds)*	11.6 (3.4 to 19.8)	408.3 (380.8 to 435.8)	1,424.2 (1,378.3 to 1,470.1)	4.09 (−12.24 to 20.42)	108.71 (53.73 to 163.69)	353.33 (260.16 to 446.5)
	Primary care cost avoided	1.59 (−1 to 4.18)	50.27 (41.64 to 58.9)	167.16 (152.8 to 181.52)	0.69 (−2.01 to 3.39)	19.89 (10.89 to 28.89)	65.63 (50.61 to 80.65)
	Secondary care cost avoided	5.56 (−0.31 to 11.42)	196.62 (177.09 to 216.15)	683.71 (651 to 716.42)	0.97 (−2.65 to 4.59)	27.94 (15.91 to 39.97)	92.1 (72.08 to 112.12)
	Medication cost avoided	2.91 (−0.38 to 6.2)	102.95 (91.97 to 113.93)1	362.59 (344.2 to 380.98)	0.56 (−1.53 to 2.65)	16.07 (9.12 to 23.02)	53.05 (41.47 to 64.63)
	Social care cost avoided	1.51 (−2.51 to 5.53)	58.48 (45.1 to 71.86)	210.73 (188.45 to 233.01)	1.87 (−13.68 to 17.42)	44.81 (−7.62 to 97.24)	142.55 (53.55 to 231.55)
**EU limit values met**	**Cumulative incidence of cases**	Not applicable			652,106 (650,261 to 653,951)	6,119,176 (6,113,481 to 6,124,871)	13,630,127 (13,621,587 to 13,638,667)
	**Cumulative incidence of cases avoided (relative to baseline)**				9,458 (6,909 to 12,007)	96,786 (88,916 to 104,656)	225,465 (213,665 to 237,265)
	Total cost avoided relative to baseline (millions of British pounds)*	Not applicable			12.92 (−3.41 to 29.25)	442.81 (387.84 to 497.78)	1,692.87 (1,599.75 to 1,785.99)
	Primary care cost avoided				2.34 (−0.36 to 5.04)	83.19 (74.2 to 92.18)	324.1 (309.11 to 339.09)
	Secondary care cost avoided				3.25 (−0.37 to 6.87)	116.9 (104.88 to 128.92)	456.06 (436.08 to 476.04)
	Medication cost avoided				2.02 (−0.07 to 4.11)	71.7 (64.75 to 78.65)	280.19 (268.63 to 291.75)
	Social care cost avoided				5.31 (−10.24 to 20.86)	171.02 (118.6 to 223.44)	632.52 (543.56 to 721.48)

Scaling to the population of England may lead to rounding discrepancies between cases and costs avoided.

*All costs are in millions of British pounds and are for the total population of England.

## Discussion

In England, in 2017, PM_2.5_ was estimated to cost the NHS and social care £76.10 million and NO_2_ was estimated to cost £81.06 million. These estimated costs are the result of 63,430 attributable incident cases of PM_2.5_-related diseases and 60,648 attributable incident cases of NO_2_-related diseases occurring in 2017. This accounts for around 10% of the total burden of diseases related to PM_2.5_ and around 9% of the total burden of diseases related to NO_2_ in 2017. Between 2017 and 2025, the total cost to the NHS and social care of air pollution in England was estimated to be £1.54 billion for PM_2.5_ and £60.81 million for NO_2_, increasing to £2.81 billion and £2.75 billion, respectively, when all diseases associated with these pollutants are included (strong and weaker evidence combined). Secondary care was estimated to contribute most to PM_2.5_-related costs, and social care to contribute most to NO_2_-related costs. This latter finding is due to the inclusion of dementia as a disease related to NO_2_ (but not PM_2.5_), which has a very high social care cost burden. We estimated that reducing each pollutant by 1 μg/m^3^ in 1 year alone would have important long-term impacts on health. Avoiding disease cases is necessary if disease burden from air pollution is to be reduced.

This study complements existing work in a number of ways. First, using population attributable fractions, studies have found that outdoor air pollution contributes to around 40,000 premature deaths a year [27], of which 29,000 deaths are attributed to PM_2.5_ [28] and 11,000 to NO_2_ [29,30]. Second, previous calculations costed mortality due to air pollution, estimating between £8.5 billion and £20.2 billion a year [4], based on the ‘willingness to pay’ approach [31]. In a complementary approach, the costs quantified in the present study represent the costs of treating air-pollution-related diseases in the NHS and social care system. Finally, this study also complements the work by COMEAP on the short-term impacts of NO_2_ on health while extending their work on long-term impacts [32].

This study has both strengths and limitations. The use of a microsimulation model is a key strength of this study since it models many millions of individuals over time (rather than groups/cohorts using weighted averages, as in many studies) and records this history to determine an individual’s future risk of NCDs over the long term. However, this approach prevented us from estimating the impact of short-term risks, since we were interested in annual disease and cost outputs. Short-term peaks in air pollution are unpredictable, both temporally and geospatially, and driven by short-term meteorology, and are therefore not straightforward to predict based on annual averages alone. Short-term peaks may exacerbate disease and increase secondary care, therefore causing greater NHS costs. However, it is unclear how short-term and long-term risks overlap. Further, to analyse short-term risks, the microsimulation model would require daily/monthly disease incidence data in order to initialise the microsimulation model population and quantify the impact of a daily spike in air pollution versus baseline levels, data for which are not available. Further, we assumed a flat trend of pollutant exposure into the future. Modelling dynamic future air pollution trends would require establishing future emission and policy scenarios that could inform the model. Modelling dynamic future trends is very complex, as illustrated by Williams et al. [33], and often results in unintended consequences (such as the promotion of diesel fuel 2 decades ago and the resultant increase in nitrogen dioxide levels). For this study, we therefore decided against modelling future air pollution scenarios and their related uncertainties and instead focused on scenarios that impact the study population equally, e.g., reduction of exposure levels by 1 μg/m^3^. The set-up of the microsimulation would, however, allow running specific scenarios in the future.

One limitation of the model is that it does not incorporate the effect of uncertainty of parameters, such as the distribution of standard errors from the air pollution surfaces, which would alter the surface and consequently the postcode estimates and resulting cost estimates. Such uncertainty might arise from the use of ambient concentrations at place of residence (the exposure metrics that are used for most long-term air pollution exposure studies such as those used to derive the exposure–response functions used in this study) rather than personal exposure. Similarly, the uncertainty around the exposure–response association is likely to impact findings since effect estimates were obtained from meta-analyses of prospective longitudinal studies, which cannot adjust for all potential confounding. The robustness of effect sizes varied across the conditions included in the model, and some emerging exposure–response relationships, i.e., for dementia and low birth weight, were obtained from a more recent, developing evidence base. We noted the difference in robustness, and interpret the results including the costs of dementia and low birth weight with more caution. The microsimulation was also limited by the availability and quality of the cost data, most of which were extracted from the literature, so they are not fully comparable. However, their different magnitudes are reliable estimates of the cost burdens. In many cases the cost estimates represent lower bound estimates of the true costs to the health and social care systems; therefore, our results are likely to underestimate the true burden. Further work is required, potentially using primary care datasets to gather more up-to-date and accurate estimates of healthcare utilisation and the cost per case of each disease. As the main objective of this project was to estimate the direct cost of pollution for the NHS and social care, societal costs, such as sick leave and loss of income, were not accounted for. Therefore, one should bear in mind that the costs reported here represent only a share of the overall societal costs related to air pollution.

There is a lack of reporting of uncertainty on input data as only dose–response uncertainties were available in the literature (S2 Text). Furthermore, correlation between the model parameters as well as the complexity and non-linearity of the microsimulation meant that we could only include Monte Carlo errors using a normal distribution around the outputs. Indeed, parametric uncertainty analysis using a highly complex tool such as the UK Health Forum microsimulation would require running many thousands of consecutive runs, and would require a super computer. Unfortunately, it was beyond the resources and scope of the present project to do this.

This study had a relatively narrow focus on just PM_2.5_ and NO_2_; therefore, we are likely to be underestimating the full impact of air pollution on the NHS and social care. Future work should build on this model to include additional pollutants such as ozone, ultra-fine particulates, and volatile organic compounds. Using COMEAP recommendations for mortality, we adjusted NO_2_ dose–response relationships by 60% to take account of overlapping effects with PM_2.5_ [26]. However, no guidance exists for possible adjustments for PM_2.5_ dose–response metrics. Future work might model multiple interacting pollutant risks for morbidity outcomes.

While England has reduced PM_2.5_ levels to below the limit values set by the EU, the magnitude of the number of cases and the NHS and social care costs that could potentially be avoided by reaching EU targets for NO_2_ makes a strong argument for policy action towards significantly reducing air pollution concentrations in England, to improve the quality of life of individuals as well as reduce current pressures on the health system, specifically secondary and social care.

This study therefore has important implications for policy makers since it makes the economic case for investment in interventions to reduce air pollution and substantiates why exposure to air pollution is a key public health priority, as highlighted in Public Health England’s 2016–2017 remit letter [34]. This study supports work that sets out actions to be taken to reduce air pollution, namely the Department for Environment, Food and Rural Affairs’ report on reducing roadside NO_2_ [35] and National Institute for Health and Care Excellence guidance on traffic-related air pollution [36], and provides evidence to support third sector campaigns, such as the campaign for a new Clean Air Act [37] and Client Earth’s legal action to bring down illegal levels of pollution [38]. Measures aimed at reducing air pollution do not just impact health but, importantly, could have many co-benefits, such as reducing road traffic accidents [39], increasing use of public transport, supporting sustainable development [40], mitigating climate change [41], reducing noise pollution [42], improving mental health and well-being [43], increasing workers’ productivity [44], increasing active travel and consequently physical activity [45,46], and improving the health of vulnerable groups (children, the elderly, and socioeconomically deprived individuals) [47].

More work is needed to build on the current evidence base. The microsimulation model presented here is a flexible and validated tool that can easily be expanded to include additional pollutants and different populations (e.g., local authorities) and to test the long-term effectiveness and cost-effectiveness of a range of different interventions to reduce future air-pollution-related disease burden. The microsimulation also has the capability to include non-healthcare costs due to disease burden (i.e., indirect costs due to lost productivity) so a wider societal approach could be taken. Future work might also explore combining methods such as a spatial agent-based modelling approach with a microsimulation to enable short- and long-term effects to be modelled together or running additional pollutants in the model.

Investment in reducing air pollution is crucial if a broad range of benefits related to health, quality of life, and the wider society are to be gained; therefore, controlling and reducing air pollution should be a key priority for the UK Government. Effective interventions should be implemented through collaboration between environmental, town planning, and public health policy makers, ensuring that environmental policies consider health as a primary outcome.

## Supporting information

S1 Text Technical appendix. (DOCX)

S2 Text Population and disease data. (DOCX)

S3 Text Cost methods. (DOCX)

S4 Text Results by disease and cost parameter. (DOCX)

## Abbreviations

COMEAP: Committee on the Medical Effects of Air Pollution
COPD: and chronic obstructive pulmonary disease
HES: Hospital Episode Statistics
NCD: non-communicable disease
NHS: National Health Service
ONS: Office for National Statistics
