# Supplementary material for: Estimating the costs of air pollution to the National Health Service and social care: An assessment and forecast up to 2035
Source: PLoS Med. 2018 Jul 10;15(7):e1002602. doi: 10.1371/journal.pmed.1002602 (PMC6039053; doi:10.1371/journal.pmed.1002602)
Supplement: S1 Text — (DOCX) [file pmed.1002602.s001.docx]

## S1 TEXT: TECHNICAL APPENDIX

# Microsimulation framework

Our simulation consists of two modules. The first module calculates the predictions of risk factor trends over time based on data from rolling cross-sectional studies. The second module performs the microsimulation of a virtual population, generated with demographic characteristics matching those of the observed data. The health trajectory of each individual from the population is simulated over time allowing them to contract, survive or die from a set of diseases or injuries related to the analysed risk factors. The detailed description of the two modules is presented below.

# Microsimulation Module one: Predictions of PM_2.5_ and NO_2_ over time

NO_2_ and PM_2.5_ are analysed within the model as risk factors (RF), as described in Table A.

**Table A Description of the categories used for the risk factors NO2 and PM2.5 (based on tertiles across all postcodes in England)**

| Risk factor (RF) | Number of categories (N) | Categories |
| --- | --- | --- |
| Particulate matter (PM_2.5_) | 3 | PM_2.5_ < 12.3 µg/m^3^  PM_2.5_ ≥ 12.3 to 13.5 µg/m^3^  PM_2.5_ ≥ 13.5 µg/m^3^ |
| Nitrogen dioxide (NO_2_) | 3 | NO_2_ < 20.5 µg/m^3^  NO_2_ ≥ 20.5 to 28.5 µg/m^3^  NO_2_ ≥ 28.5 µg/m^3^ |

For the RF, let *N* be the number of categories for a given risk factor, e.g. *N* = 3 for NO_2_. Let $k$= 1, 2, …, *N* number these categories and $p_{k}(t)$ denote the prevalence of individuals with RF values that correspond to the category $k$ at time *t*. We estimate $p_{k}(t)$ using multinomial logistic regression model with prevalence of RF category $k$ as the outcome, and time *t* as a single explanatory variable. For $k<N$, we have

$$\ln\left(\frac{p_{k}\left(t\right)}{p_{1}\left(t\right)}\right)=\beta_{0}^{k}+\beta_{1}^{k}t$$

The prevalence of the first category is obtained by using the normalisation constraint $\sum_{k=1}^{N} p_{k}(t)=1$. Solving equation for $p_{k}(t)$, we obtain

$$p_{k}\left(t\right)=\frac{\exp\left(\beta_{0}^{k}+\beta_{1}^{k}t\right)}{1+\sum_{\text{k}'=1}^{\text{N}} \text{exp}\left(\beta_{0}^{k'}+\beta_{1}^{k'}t\right)} ,$$

which respects all constraints on the prevalence values, i.e. normalisation and [0, 1] bounds.

## Multinomial logistic regression for each risk factor

Measured data consist of sets of probabilities, with their variances, at specific time values (typically the year of the data were collected). For any particular time the sum of these probabilities is unity. Typically such data might be the probabilities of low, medium and high pollution exposure, as they are extracted from the data set. Each data point is treated as a normally distributed[[1]](#footnote-1) random variable; together they are a set of *N* groups (number of years: 18 years) of *K* probabilities {{*t*_i_, μ_ki_, σ_ki_ |*k*∈[0,*K*-1]} | *i*∈[0,*N*-1]}. For each year the set of *K* probabilities form a distribution – their sum is equal to unity.

The regression consists of fitting a set of logistic functions {*p*_k_(**a**, **b***, t*)|*k*∈[0,*K*-1]} to these data – one function for each *k*-value. At each time value the sum of these functions is unity. Thus, for example, when measuring NO_2_ in the three states already mentioned, the *k*= 0 regression function represents the probability of low pollution exposure over time, *k*= 1 the probability of medium pollution exposure and *k*= 2 the probability of high pollution exposure.

The regression equations are most easily derived from a familiar least square minimization. In the following equation set the weighted difference between the measured and predicted probabilities is written as *S*; the logistic regression functions *p_k_*(**a**,**b***;t*) are chosen to be ratios of sums of exponentials (this is equivalent to modelling the log probability ratios, *p_k_*/*p*_0_, as linear functions of time).

l

$$\begin{matrix} p_{k}\left(\mathbf{a},\mathbf{b},t\right)\equiv\frac{e^{A_{k}}}{1+e^{A_{1}}+..+e^{A_{K-1}}} \\ \mathbf{a}\equiv\left(a_{0},a_{1},..,a_{K-1}\right), \mathbf{b}\equiv\left(b_{0},b_{1},..,b_{K-1}\right) \\ A_{0}\equiv0, A_{k}\equiv a_{k}+b_{k}t \end{matrix}$$

The parameters *A*_0_, *a*_0_ and *b*_0_ are all zero and are used merely to preserve the symmetry of the expressions and their manipulation. For a *K*-dimensional set of probabilities there will be 2(*K*-1) regression parameters to be determined.

For a given dimension *K* there are *K*-1 independent functions *p_k_* – the remaining function being determined from the requirement that complete set of *K* form a distribution and sum to unity.

Note that the parameterization ensures that the necessary requirement that each *p_k_* be interpretable as a probability – a real number lying between 0 and 1.

The minimum of the function *S* is determined from the equations

$$\frac{\partial S}{\partial a_{j}}=\frac{\partial S}{\partial b_{j}}=0\text{ for j=1,2,....,k-1}$$

noting the relations

$$\begin{matrix} \frac{\partial p_{k}}{\partial A_{j}}=\frac{\partial}{\partial A_{j}}\left(\frac{e^{A_{k}}}{1+e^{A_{1}}+..+e^{A_{K-1}}}\right)=p_{k}\delta_{kj}-p_{k}p_{j} \\ \frac{\partial}{\partial a_{j}}=\frac{\partial}{\partial A_{j}} \\ \frac{\partial}{\partial b_{j}}=t\frac{\partial}{\partial A_{j}} \end{matrix}$$

The values of the vectors **a**, **b** that satisfy these equations are denoted $\hat{\mathbf{a}}\mathbf{,}\hat{\mathbf{b}}$. They provide the trend lines, for the separate probabilities. The confidence intervals for the trend lines are derived most easily from the underlying Bayesian analysis of the problem.

## Bayesian interpretation

The 2*K*-2 regression parameters {**a,b**} are regarded as random variables whose posterior distribution is proportional to the function exp(-*S*(**a**,**b**)). The maximum likelihood estimate of this probability distribution function, the minimum of the function S, is obtained at the values $\hat{\mathbf{a}}\mathbf{,}\hat{\mathbf{b}}$. Other properties of the (2*K*-2)-dimensional probability distribution function are obtained by first approximating it as a (2*K*-2)-dimensional normal distribution whose mean is the maximum likelihood estimate. This amounts to expanding the function *S*(**a**,**b**) in a Taylor series as far as terms quadratic in the differences $\left(\mathbf{a}-\hat{\mathbf{a}}\right)()\mathbf{,}\left(\mathbf{b}-\hat{\mathbf{b}}\right)()$ about the maximum likelihood estimate . Hence

The (2*K*-2)-dimensional covariance matrix *P* is the inverse of the appropriate expansion coefficients. This matrix is central to the construction of the confidence limits for the trend lines.

### Estimation of the confidence intervals

The logistic regression functions *p*_k_(*t*) can be approximated as a normally distributed time-varying random variable by expanding *p*_k_ about its maximum likelihood estimate (the trend line)

$$\begin{matrix} p_{k}\left(\mathbf{a},\mathbf{b},t\right)=p_{k}\left(\hat{\mathbf{a}}+\mathbf{a}-\hat{\mathbf{a}},\hat{\mathbf{b}}+\mathbf{b}-\hat{\mathbf{b}},t\right) \\ =\hat{p}_{k}\left(t\right)+\left(\nabla_{\hat{a}},\nabla_{\hat{b}}\right)\hat{p}_{k}\left(t\right)\left(\begin{matrix} \mathbf{a}-\hat{\mathbf{a}} \\ \mathbf{b}-\hat{\mathbf{b}} \end{matrix}\right)+... \end{matrix}$$

Denoting mean values by angled brackets, the variance of *p*_k_ is thereby approximated as

$$\begin{matrix} \sigma_{k}^{2}\left(t\right)\equiv\left\langle\left(p_{k}\left(\mathbf{a},\mathbf{b},t\right)-\hat{p}_{k}\left(t\right)\right)^{2}\right\rangle=\left(\nabla_{\hat{a}}\hat{p}_{k}\left(t\right),\nabla_{\hat{b}}\hat{p}_{k}\left(t\right)\right)\left\langle\left(\begin{matrix} \mathbf{a}-\hat{\mathbf{a}} \\ \mathbf{b}-\hat{\mathbf{b}} \end{matrix}\right)\left(\begin{matrix} \mathbf{a}-\hat{\mathbf{a}} \\ \mathbf{b}-\hat{\mathbf{b}} \end{matrix}\right)^{T}\right\rangle\times\\ \left(\nabla_{\hat{a}}\hat{p}_{k}\left(t\right),\nabla_{\hat{b}}\hat{p}_{k}\left(t\right)\right)^{T}=\left(\nabla_{\hat{a}}\hat{p}_{k}\left(t\right),\nabla_{\hat{b}}\hat{p}_{k}\left(t\right)\right)\text{ }P\text{ }\left(\nabla_{\hat{a}}\hat{p}_{k}\left(t\right),\nabla_{\hat{b}}\hat{p}_{k}\left(t\right)\right)^{T} \end{matrix}$$

When *K*=3 this equation can be written as the 4-dimensional inner product

$$\sigma_{k}^{2}\left(t\right)=\left(\begin{matrix} \frac{\partial\hat{p}_{k}\left(t\right)}{\partial\hat{a}_{1}} & \frac{\partial\hat{p}_{k}\left(t\right)}{\partial\hat{a}_{2}} & \frac{\partial\hat{p}_{k}\left(t\right)}{\partial\hat{b}_{1}} & \frac{\partial\hat{p}_{k}\left(t\right)}{\partial\hat{b}_{2}} \end{matrix}\right)\left[\begin{matrix} P_{aa11} & P_{aa12} & P_{ab11} & P_{ab12} \\ P_{aa21} & P_{aa22} & P_{ab21} & P_{ab22} \\ P_{ba11} & P_{ba12} & P_{bb11} & P_{bb12} \\ P_{ba21} & P_{ba22} & P_{bb21} & P_{bb22} \end{matrix}\right]\left(\begin{matrix} \frac{\partial\hat{p}_{k}\left(t\right)}{\partial\hat{a}_{1}} \\ \frac{\partial\hat{p}_{k}\left(t\right)}{\partial\hat{a}_{2}} \\ \frac{\partial\hat{p}_{k}\left(t\right)}{\partial\hat{b}_{1}} \\ \frac{\partial\hat{p}_{k}\left(t\right)}{\partial\hat{b}_{2}} \end{matrix}\right)$$

where . The 95% confidence interval for *p*_k_(*t*) is centred given as .

# Module two: Microsimulation model

## Microsimulation initialisation: birth, disease and death models

Simulated people are generated with the correct demographic statistics in the simulation’s start-year. In this year women are stochastically allocated the number and years of birth of their children – these are generated from known fertility and mother’s age at birth statistics (valid in the start-year) [1]. If a woman has children then those children are generated as members of the simulation in the appropriate birth year.

The microsimulation is provided with a list of air pollution-related diseases. These diseases used the best available incidence, mortality, survival, relative risk and prevalence statistics (by age and gender) (see S1 Table 5, appendix 2 for references). Individuals in the model are simulated from their year of birth (which may be before the start year of the simulation). In the course of their lives, simulated people can die from one of the diseases caused by an air pollutant that they might have acquired or from some other cause(s). The probability that a person of a given age and gender dies from a cause other than the disease are calculated in terms of known death and disease statistics valid in the start-year. It is constant over the course of the simulation.

The microsimulation incorporates a sophisticated economic module. The module employs a Markov-type simulation of long-term health benefits and health care costs. It synthesises and estimates evidence on cost-utility analysis. The model is used to project the differences in quality-adjusted life years (QALYs), and direct lifetime health-care costs over a specified time scale. The direct healthcare costs are presented separately in terms of hospital admissions, general practitioner costs, medication costs and social care costs. Outputs can be discounted for any specific discount rate.

This following section provides an overview of the main assumptions of the model.

## Population models

Populations are implemented as instances of the TPopulation C++ class. The TPopulation class is created from a population (*.ppl) file. Usually a simulation will use only one population but it can simultaneously process multiple populations (for example, different ethnicities within a national population).

### Population Editor

The Population Editor Allows editing and testing of TPopulation objects. The population is created in the start-year and propagated forwards in time. An example population pyramid which can be used when initialising the model is shown in S1 Figure 1 shows the population distribution for England in 2015 used in the initialisation of the model.


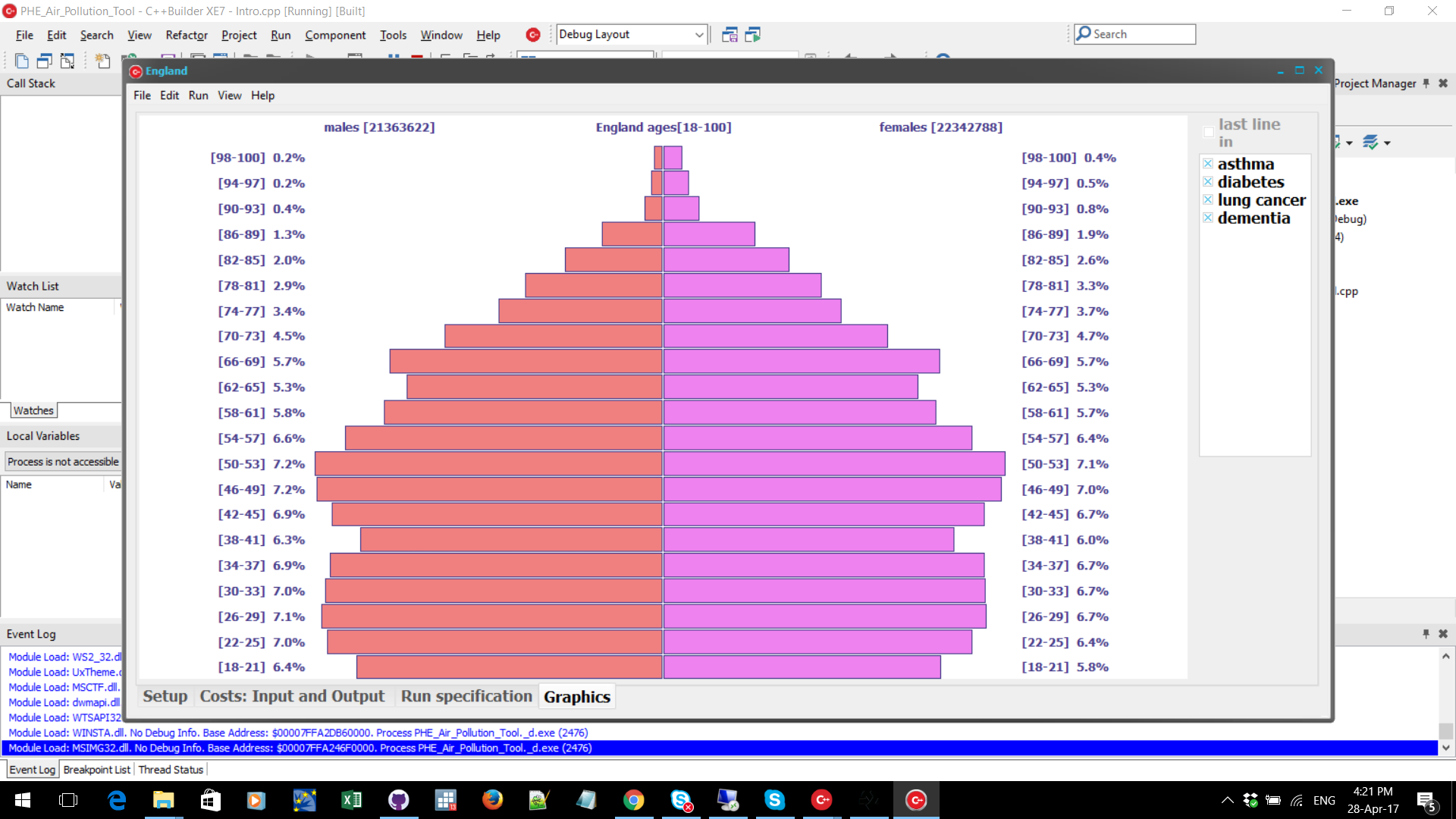


Figure A Population pyramid for England in 2015

People within the model can die from specific diseases or from other causes. A disease file is created within the program to represent deaths from other causes. The following distributions are required by the population editor (**Table B**).

**Table B Summary of the parameters representing the distribution component**

| Distribution name | symbol | note |
| --- | --- | --- |
| MalesByAgeByYear | $p_{m}\left( a \right)$ | Input in year0 – probability of a male having age a |
| FemalesByAgeByYear | $p_{f}\left( a \right)$ | Input in year0 – probability of a female having age a |
| BirthsByAgeofMother | $p_{b}\left( a \right)$ | Input in year0 – conditional probability of a birth at age a| the mother gives birth. |
| NumberOfBirths |  | λ≡TFR, Poisson distribution, probability of giving birth to n children |

#### Birth model

Any female in the child bearing years is deemed capable of giving birth. The number of children, n, that she has in her life is dictated by the Poisson distribution where the mean of the Poisson distribution is the Total Fertility Rate (TFR) parameter[[2]](#footnote-2)[2].

The probability that a mother (who does give birth) gives birth to a child at age a is determined from the BirthsByAgeOfMother distribution as $p_{b}\left( a \right)$. For any particular mother the births of multiple children are treated as independent events, so that the probability that a mother who produces N children produces n of them at age a is given as the Binomially distributed variable,

$$p_{b}\left(\text{n at a}|N\right)= \frac{N!}{n!\left(N-n\right)!}\left(p_{b}\left(a\right)\right)^{n}\left(1-p_{bm}\left(a\right)\right)^{N-n}$$

The probability that the mother gives birth to n children at age a is

$$p_{b}\left(n at a\right)= e^{-\lambda}\sum_{N=n}^{\infty} \frac{\lambda^{N}}{N!}p_{b}\left(\text{n at a}|N\right)= e^{-\lambda}\sum_{N=n}^{\infty} \frac{\lambda^{N}}{n!\left(N-n\right)!}\left(p_{b}\left(a\right)\right)^{n}\left(1-p_{b}\left(a\right)\right)^{N-n}$$

Performing the summation in this equation gives the simplifying result that the probability *p_b_*(*n at a*) is itself Poisson distributed with mean parameter ${\lambda p}_{b}\left( a \right)$,

$$p_{b}\left(n at a\right)= e^{-\lambda p_{b}\left(a\right)}\frac{\left(\lambda p_{b}\left(a\right)\right)^{n}}{n!}=p_{\lambda p_{b}\left(a\right)}\left(n\right)$$

Thus, on average, a mother at age $a$ will produce ${\lambda p}_{b}\left( a \right)$ children in that year.

The gender of the children[[3]](#footnote-3) is determined by the probability *p_male_*=1-*p_female_*. In the baseline model this is taken to be the probability *N_m_*/(*N_m_*+*N_f_*).

The Population editor’ menu item Population Editor\Tools\Births\show random birthList creates an instance of the TPopulation class and uses it to generate and list a (selectable) sample of mothers and the years in which they give birth.

### Deaths from modelled diseases

The simulation models any number of specified diseases some of which may be fatal. In the start year the simulation’s death model uses the diseases’ own mortality statistics to adjust the probabilities of death by age and gender. In the start year the net effect is to maintain the same probability of death by age and gender as before; in subsequent years, however, the rates at which people die from modelled diseases will change as modelled risk factors change.

## The risk factor model

The distribution of risk factors (RF) in the population is estimated using regression analysis stratified by both sex *S* = {male, female} and age group *A* = {0-4, 5-9, ..., 70-74, 75+}. The fitted trends are extrapolated to forecast the distribution of each RF category in the future. For each sex-and-age-group stratum, the set of cross-sectional, time-dependent, discrete distributions $D=\{p_{k}\left( t \right)|k=1,\ldots N; t>0\}$, is used to manufacture RF trends for individual members of the population. Each air pollutant (e.g. NO_2_, PM2.5) is modelled as a continuous risk factor.

### Continuous risk factors

In the case of a continuous RF, for each discrete distribution $D$ there is a continuous counterpart. Let $\beta$ denote the RF value in the continuous scale and let $f(\beta|A,S,t)$ be the probability density function of $\beta$ for age group $A$ and sex $S$ at time $t$. Then

$$p_{k}\left(t\text{|}A,S\right)=\int_{\beta\in k} f\left(\beta\text{|}A,S,t\right)d\beta.$$

Equation and both refers to the same quantity. Equation uses the definition of the probability density function to express the age-and-sex-specific percentage of individuals in RF category *k* at time *t*. Equation gives an estimate of this quantity using equation for all *k* = 0, …, N. The cumulative distribution function of $\beta$ is

$$F\left(\beta\text{|}A,S,t\right)=\int_{0}^{\beta} f\left(\beta\text{|}A,S,t\right)d\beta.$$

At time *t*, a person with sex $S$ belonging to the age group $A$ is said to be on the $p$–th percentile of this distribution if $F\left( \beta| A,S,t \right)=p/100.$ Given the cross-sectional information from the set of distributions $D$, it is possible to simulate longitudinal trajectories by forming pseudo-cohorts within the population. A key requirement for these sets of longitudinal trajectories is that they reproduce the cross-sectional distribution of RF categories for any year with available data. The method adopted here and in our earlier work (1) is based on the assumption that person’s RF value changes throughout their lives in such a way that they always have the same associated percentile rank. As they age, individuals move from one age group to another and their RF value changes so that they have the same percentile rank but of a different RF distribution. Crucially it meets the important condition that the cross-sectional RF distributions obtained by simulation match the RF distributions of the observed data.

The above procedure can be explained using the example of the NO_2_ distribution. The NO_2_ distributions are known for the population stratified by sex and age for all years of the simulation (by extrapolation of fitted model, see equation ). A person who is in age group $A$ and who grows ten years older will at some time move into the next age group $A'$ and will have a BMI that was described first by the distribution $f(\beta|A,S,t)$ and then at the later time $t'$ by the distribution $f(\beta|A',S,t')$. If the NO_2_ exposure level of that individual is on the $p$*-*th percentile of the NO_2_ distribution, their NO_2_ exposure level will change from $\beta$ to $\beta'$ so that

$$\beta=F^{-1}\left(\frac{p}{100}\text{|}A,S,t\right)$$

$$\beta'=F^{-1}\left(\frac{p}{100}\text{|}A',S,t'\right)\mathop{\Rightarrow}\limits\beta'=F^{-1}\left(F(\beta|A,S,t)\text{|}A',S,t'\right)$$

Where $F^{-1}$ is the inverse of the cumulative distribution function of $\beta$, which we model with a continuous uniform distribution within the RF categories (see **Error! Reference source not found.**). Equation guarantees that the transformation taking the random variable $\beta$ to $\beta'$ ensures the correct cross-sectional distribution at time $t'$..

The microsimulation first generates individuals from the RF distributions of the set $D$ and, once generated, grows the individual’s RF in a way that is also determined by the set $D$. It is possible to implement equation as a suitably fast algorithm.

## Relative risks

Suppose that α is a risk factor state of some risk factor Α and denote by p_A­_ (d|α,a,s) the incidence probability for the disease d given the risk state, α, the person’s age, a, and gender, s. The relative risk ρ_A_ is defined by equation .

$$\begin{matrix} p_{A}\left(d\left|\alpha,a,s\right.\right)=\rho_{A|d}\left(\alpha\left|a,s\right.\right)p_{A}\left(d\left|\alpha_{0},a,s\right.\right) \\ \rho_{A|d}\left(\alpha_{0}\left|a,s\right.\right)\equiv1 \end{matrix}$$

Where α_0_ is the zero risk state.

The incidence probabilities, as reported, can be expressed in terms of the equation,

$$\begin{matrix} p\left(d\left|a,s\right.\right)=\sum_{\alpha} p_{A}\left(d\left|\alpha,a,s\right.\right)\pi_{A}\left(\alpha\left|a,s\right.\right) \\ =p_{A}\left(d\left|\alpha_{0},a,s\right.\right)\sum_{\alpha} \rho_{A|d}\left(\alpha\left|a,s\right.\right)\pi_{A}\left(\alpha\left|a,s\right.\right) \end{matrix}$$

Combining these equations allows the conditional incidence probabilities to be written in terms of known quantities

$$p\left(d\left|\alpha,a,s\right.\right)=\rho_{A|d}\left(\alpha\left|a,s\right.\right)\frac{p\left(d\left|a,s\right.\right)}{\sum_{\beta} \rho_{A|d}\left(\beta|a,s\right)\pi_{A}\left(\alpha\left|a,s\right.\right)}$$

Previous to any series of Monte Carlo trials the microsimulation program pre-processes the set of diseases and stores the *calibrated* incidence statistics *p_A_* (*d*|*α*_0_, *a*, *s*). These incidence statistics are calibrated to national level data. In this project the risk factor distributions and incidence risks for England are used to calculate the calibrated risks.

## Modelling diseases

Disease modelling relies heavily on the sets of incidence, mortality, survival, relative risk and prevalence statistics. In some cases where a data set is unavailable or not available is the specified form for the model, data has been approximated from the known sets of the data.

The microsimulation uses risk dependent incidence statistics and these are inferred from the relative risk statistics and the distribution of the risk factor within the population. In the simulation, individuals are assigned a risk factor trajectory giving their personal risk factor history for each year of their lives. Their probability of getting a particular risk factor related disease in a particular year will depend on their risk factor state in that year.

Once a person has a fatal disease (or diseases) their probability of survival will be controlled by a combination of the disease-survival statistics and the probabilities of dying from other causes. Disease survival statistics are modelled as age and gender dependent exponential distributions.

### Mortality statistics

In any year, in some population, in a sample of N people who have the disease a subset $N_{\omega}$ will die from the disease.

Mortality statistics record the cross sectional probabilities of death as a result of the disease – possibly stratifying by age

$$p_{\omega}=\frac{N_{\omega}}{N}$$

Within some such subset $N_{\omega}$of people that die in that year from the disease, the distribution by year-of-disease is not usually recorded. This distribution would be most useful. Consider two important idealised, special cases

Suppose the true probabilities of dying in the years after some age $a_{0}$ are $\left\{p_{\omega0},p_{\omega1},p_{\omega2},p_{\omega3},p_{\omega4}\right\}$

The probability of being alive after N years is simply that you don’t die in each year

$$p_{survive}\left(a_{0}+N\right)=\left(1-p_{\omega0}\right)\left(1-p_{\omega1}\right)\left(1-p_{\omega2}\right)..\left(1-p_{\omega N-1}\right)$$

### Survival rates

It is common practice to describe survival in terms of a survival rate R, supposing an exponential death-distribution. In this formulation the probability of surviving t years from some time t_0_ is given as

$$p_{\text{survival}}\left(t\right)=1-R\int_{0}^{t} due^{-Ru}=e^{-Rt}$$

For a time period of 1 year

$$\begin{matrix} p_{survival}\left(1\right)=e^{-R} \\ \Rightarrow\\ R=-\ln\left(p_{survival}\left(1\right)\right)=-\ln\left(1-p_{\omega}\right) \end{matrix}$$

For a time period of, for example, 4 years,

$$p_{\text{survival}}\left(t=4\right)=1-R^{-1}\int_{0}^{4} due^{-Ru}=e^{-4R}=\left(1-p_{\omega}\right)^{4}$$

In short, the Rate is minus the natural log of the 1-year survival probability.

### The survival models

For any potentially terminal disease the model can use any of the three survival models, numbered ((0, 1, 2)). The parameters describing these models are given below.

**Survival model 0**

A single probability of dying $\left\{p_{\omega0}\right\}$, where $p_{\omega0}$ is valid for all years. Given the 1-year survival probability $p_{survival}\left(1\right)$

The model uses 1 parameter ((R))

$$R=-\ln\left(p_{survival}\left(1\right)\right)$$

**Survival model 1**

Two different probabilities of dying $\left\{p_{\omega0},p_{\omega1}\right\}$, where $p_{\omega0}$ is valid for the first year; $p_{\omega1}$thereafter. The model uses two parameters ((p_1_, R)). Given the 1-year survival probability $p_{survival}\left(1\right)$and the 5-year survival probability $p_{survival}\left(5\right)$

$$\begin{matrix} p_{1}=1-p_{survival}\left(1\right) \\ R=-\frac{1}{4}\ln\left(\frac{p_{survival}\left(5\right)}{p_{survival}\left(1\right)}\right) \\ \end{matrix}$$

**Survival model 2**

Three different probabilities of dying $\left\{p_{\omega0},p_{\omega1},p_{\omega5}\right\}$, where $p_{\omega0}$ is valid for the first year; $p_{\omega1}$for the second to the fifth year;$p_{\omega5}$thereafter. The model uses three parameters ((p_1_, R, R_>5_))

Given the 1-year survival probability $p_{survival}\left(1\right)$and the 5-year survival probability $p_{survival}\left(5\right)$

$$\begin{matrix} p_{1}=1-p_{survival}\left(1\right) \\ R=-\frac{1}{4}\ln\left(\frac{p_{survival}\left(5\right)}{p_{survival}\left(1\right)}\right) \\ R_{>5}=-\frac{1}{5}\ln\left(\frac{p_{survival}\left(10\right)}{p_{survival}\left(5\right)}\right) \end{matrix}$$

Remember that different probabilities will apply to different age and gender groups. Typically the data might be divided into 10 year age groups.

### Modelling low birth weight

The modelling method assumes that low birth weight (LBW) is a disease associated with a woman who gives birth. The method also assumes that LBW is an acute disease; an incidence case in any year affects the prevalence rate in that year only. In the start year of the simulation the total of number of births associated to a woman and the year of each birth is computed. The probability of a newborn being LBW is calculated using the risk factor level (i.e., air pollution level) in the year of birth and the associated relative risk. This approach is used when modelling other diseases in the simulation.

There are two differences between modelling LBW and other diseases. Firstly, a mother can have multiple births in a given year which can result in multiple incident cases of LBW. In comparison other diseases can be contracted only once in a year. Secondly, it is possible that in some years of a mother's life she does not give birth. The probability of contracting a LBW in these years is therefore zero.

**Limitations**

The modelling method assumes that LBW is a disease per se. A limitation extending from this would be that we do not take account of subsequent diseases brought about by LBW, e.g., diabetes or CHD. The model therefore underestimates the long-term economic costs of LBW associated with air pollution. Another limitation is that we allow multiple births in the simulation (e.g. twins), but we do take account of the possible impact of multiple births on LBW. Multiple births are simulated as a list of independent births having the same probability of causing LBW. No costs were available for LBW so it is removed from the main body of the text, but retained within the epidemiological analysis.

## Approximating missing disease statistics

A number of tools have been developed in the model in order to compute missing disease statistics data such as incidence or prevalence.

### Approximating survival data from mortality and prevalence

An example is provided here with a standard life-table analysis for a disease *d*.

Consider the 4 following states:

| state | Description |
| --- | --- |
| 0 | alive without disease *d* |
| 1 | alive with disease *d* |
| 2 | dead from disease *d* |
| 3 | dead from another disease |

*p_ik_* is the probability of disease *d* incidence, aged *k*

*p*_ωk_ is the probability of dying from the disease *d*, aged *k*

$p_{\bar{\omega}k}$ is the probability of dying other than from disease d, aged *k*

The state transition matrix is constructed as follows

$$\left[\begin{matrix} p_{0}\left(k+1\right) \\ p_{1}\left(k+1\right) \\ p_{2}\left(k+1\right) \\ p_{3}\left(k+1\right) \end{matrix}\right]=\left[\begin{matrix} \left(1-p_{\bar{\omega}k}\right)\left(1-p_{ik}\right) & \left(1-p_{\bar{\omega}k}-p_{\omega k}\right)p_{\alpha k} & 0 & 0 \\ \left(1-p_{\bar{\omega}k}\right)p_{ik} & \left(1-p_{\bar{\omega}k}-p_{\omega k}\right)\left(1-p_{\alpha k}\right) & 0 & 0 \\ 0 & p_{\omega k} & 1 & 0 \\ p_{\bar{\omega}k} & p_{\bar{\omega}k} & 0 & 1 \end{matrix}\right]\left[\begin{matrix} p_{0}\left(k\right) \\ p_{1}\left(k\right) \\ p_{2}\left(k\right) \\ p_{3}\left(k\right) \end{matrix}\right]$$

It is worth noting that the separate columns correctly sum to unity.

The disease mortality equation is that for state-2,

$$p_{2}\left(k+1\right)=p_{\omega k}p_{1}\left(k\right)+p_{2}\left(k\right)$$

The probability of dying from the disease in the age interval [*k*, *k*+1] is $p_{\omega k}p_{1}\left(k\right)$ - this is otherwise the (cross-sectional) disease mortality, *p_mor_*(*k*). *p*_1_(*k*) is otherwise known as the disease prevalence, *p_pre_*(*k*). Hence the relation

$$p_{\omega k}=\frac{p_{mor}\left(k\right)}{p_{pre}\left(k\right)}$$

For exponential survival probabilities the probability of dying from the disease in the age-interval [*k*, *k*+1] is denoted *p*_Ω_*_k_* and is given by the formula

$$p_{\omega k}=1-e^{-R_{k}} \Rightarrow R_{k}=-\ln\left(1-p_{\omega k}\right)$$

When, as is the case for most cancers, these survival probabilities are known the microsimulation will use them, when they are not known or are too old to be any longer of any use, the microsimulation uses survival statistics inferred from the prevalence and mortality statistics (equation ). An alternative derivation equation is as follows. Let *N*_k_ be the number of people in the population aged *k* and let *n*_k_ be the number of people in the population aged *k* with the disease. Then, the number of deaths from the disease of people aged *k* can be given in two ways: as *p*_ωk_*n*_k_ and, equivalently, as *p*_mor_(*k*)*N*_k_ . Observing that the disease prevalence is *n*_k_/*N*_k_ leads to the equation

$$\begin{matrix} p_{\Omega k}n_{k}=p_{mor}\left(k\right)N_{k} \\ p_{pre}\left(k\right)=\frac{n_{k}}{N_{k}} \\ \Rightarrow\\ p_{\Omega k}=\frac{p_{mor}\left(k\right)}{p_{pre}\left(k\right)} \end{matrix}$$

### Approximating disease incidence from prevalence

The algorithm estimates the probability of contracting a disease given age and sex, $\hat{p}(d|a,s)$ from prevalence rates, survival rates and mortality rates.

**Step 1: State transition matrix of the algorithm**

$$\left(\begin{matrix} p_{\bar{d}}(a+1|s) \\ p_{d1}(a+1|s) \\ p_{d}(a+1|s) \\ p_{dead}(a+1|s) \end{matrix}\right)=\left(\begin{matrix} (1-p_{\bar{w}}(a|s))(1-\hat{p}(d|a,s)) & 0 & 0 & 0 \\ (1-p_{\bar{w}}(a|s))\hat{p}(d|a,s) & 0 & 0 & 0 \\ 0 & 1-p_{w1+\bar{w}1}(a|s) & 1-p_{w+\bar{w}}(a|s) & 0 \\ p_{\bar{w}}(a|s) & p_{w1+\bar{w}1}(a|s) & p_{w+\bar{w}}(a|s) & 1 \end{matrix}\right)\left(\begin{matrix} p_{\bar{d}}(a|s) \\ p_{d1}(a|s) \\ p_{d}(a|s) \\ p_{dead}(a|s) \end{matrix}\right)$$

The probability of being in a set of states:

| $S_{0}$ | $p_{\bar{d}}(a\vert s)$ | The probability of being alive without disease at age $a$ |
| --- | --- | --- |
| $S_{1}$ | $p_{d1}(a\vert s)$ | The probability of being alive with new disease (contracting within a year) at age $a$ |
| $S_{2}$ | $p_{d}(a\vert s)$ | The probability of being alive with old disease at age $a$ |
| $S_{3}$ | $p_{dead}(a\vert s)$ | The probability of being dead for any reason (from the disease or other reasons) at age $a$ |

$\hat{p}(d|a,s)$ The estimated incidence probability at age of$a$given sex type $s$ .

$p_{\bar{w}}(a|s)$ The probability of dying from other causes at age of$a$ given sex type $s$.

$p_{w1+\bar{w}1}(a|s)$ The probability of dying from any reason within the first years of contracting the disease at the age of$a$ given sex type $s$.

$p_{w+\bar{w}}(a|s)$ The probability of dying from any reasons after the first years of contracting the disease at the age$a$ given sex type $s$.

$p_{survival1st}(a|s)$ The probability of surviving the first year after contracting the disease at the age of$a$ given sex type $s$.

$p_{survival1}(a|s)$ The probability of surviving the year at the age of $a$ given sex type $s$.

**Step 2: The prevalence for a particular age group**

Estimated prevalence rate can be expressed by,

where

$$\hat{P}_{pre}(a|s)=\frac{p_{d}(a|s)+p_{d1}(a|s)}{p_{d}(a|s)+p_{d1}(a|s)+p_{\bar{d}}(a|s)}$$

where $\min\_a$is the youngest age in that age group and $\max\_a$ the oldest. $\pi(a|s)$ is the population distribution stratified by age given sex.

**Step 3: Regression**

We have two algorithms to find the optimum value of $\hat{p}(d|a,s)$: simplex algorithm and cauchy algorithm. Simplex algorithm finds an optimum set of incidence rates of all age groups by minimising the distance between the estimated global prevalence rate and the actual global prevalence rate, shown in . We use simplex algorithm for most diseases as it is faster.

$$\mathop{\mathrm{argmin}}\limits_{set(\hat{p}(d|a,s))} S=\mathop{\mathrm{argmin}}\limits_{set(\hat{p}(d|a,s))} S\left(\sum_{age\_group} (P_{pre\_mean}(agegroup|s)-\hat{P}_{pre\_mean}(agegroup|s))\right)$$

Cauchy algorithm finds an optimum incidence rate for each individual age group by minimising the distance between the estimated prevalence rate and the actual prevalence rate of the age group, shown in . We use Cauchy algorithm for diseases which are associated to certain age groups, e.g., dementia which is only associated to people older than 60.

$$\mathop{\mathrm{argmin}}\limits_{\hat{p}(d|a,s)} S=\mathop{\mathrm{argmin}}\limits_{\hat{p}(d|a,s)} S\left(P_{pre\_mean}(agegroup|s)-\hat{P}_{pre\_mean}(agegroup|s)\right)$$

## Model scenarios

A baseline case and three additional scenarios were modelled. The baseline related to the current exposure data, which included background levels of air pollution. The second scenario modelled the impact of the background air pollutions levels. This scenario was used to calculate the diseases and associated costs related to air pollution.

The two additional scenarios were:

An annual decrease by 1 µg/m^3^ in PM_2.5_ and NO_2_ exposure for each individual in 2017.

A “European Limit Values” scenario whereby all the highly exposed (>40 µg/m^3^) individuals in the population of interest decrease their NO_2_ exposure to the exact European threshold (40 µg/m^3^).

# References

1. Office for National Statistics. Birth Summary Tables - England and Wales. 2016.

2. Office for National Statistics. Birth characteristics 2015 2016. Available from: <https://www.ons.gov.uk/peoplepopulationandcommunity/birthsdeathsandmarriages/livebirths/datasets/birthcharacteristicsinenglandandwales>.

1. In general, the assumption of a normal distribution is both extremely useful and accurate for both simple and complex surveys: indeed, for simple surveys the individual Bayesian prior and posterior probabilities are Beta distributions – the likelihood being binomial. For reasonably large samples, the approximation of the beta distributions by normal distributions is both legitimate and a practical necessity. For complex, multi-PSU, stratified surveys, it is again assumed that these base probabilities are approximately normally distributed and, again, it is an assumption that makes the analysis tractable.

   Depending on the nature of the raw data set it may be possible to use non-parametric statistical methods for this analysis. This is possible for the HSE and GHS data sets of this study but when this has been done the authors can report no discernible difference in the results. [↑](#footnote-ref-1)
2. This could be made to be time dependent; in the baseline model it is constant. [↑](#footnote-ref-2)
3. The probability of child gender can be made time dependent. [↑](#footnote-ref-3)
