# Supplementary material for: Estimating the costs of air pollution to the National Health Service and social care: An assessment and forecast up to 2035
Source: PLoS Med. 2018 Jul 10;15(7):e1002602. doi: 10.1371/journal.pmed.1002602 (PMC6039053; doi:10.1371/journal.pmed.1002602)
Supplement: S2 Text — (DOCX) [file pmed.1002602.s002.docx]

## S2 TEXT: POPULATION AND DISEASE DATA

# Population data

Demographic data were collected for England and Wales combined (no separate data for England was available).

Information was collected on the age and sex distribution of the population, the distribution of births by mother’s age, the total fertility rate and the distribution of deaths by age and sex.

The data were processed as text files, in a format suitable for inclusion in the microsimulation programme. **Table A** presented the data sources as follows:

**Table A. Population data sources by geography**

| Demography | Geography | Source |
| --- | --- | --- |
| Total population by age and sex | England and Wales | ONS. Population Estimates for UK, England and Wales, Scotland and Northern Ireland: mid-2015. ONS; 2016.[1] |
| Births by mothers age | England and Wales | ONS. Birth Summary Tables – England and Wales. 2015. ONS; 2016 [2] |
| Total fertility rate | England and Wales | ONS. Birth Summary Tables – England and Wales. 2015. ONS; 2016 [2] |
| Deaths by age and sex | England and Wales | ONS. Deaths registered in England and Wales 2015. ONS; 2016 [3] |

# Disease data

A number of air pollution-related diseases were modelled (see **Table B**). The list of diseases modelled for each pollutant was determined after a review of the literature available on the dose-response relationship between exposure to air pollutants and risk of incidence of disease. Diseases were included or excluded based on the existence of a dose-response relationship and availability of epidemiological data. For instance, while an association exists between lung function in children and both PM_2.5_ and NO2, this condition is not a fixed disease with identifiable prevalence, incidence and mortality data, so this condition was excluded from the disease list. Pre-term birth was also identified, but low-birth weight was considered a better proxy of the process of intrauterine growth restriction, and excluding pre-term births may avoid double counting. Low-birth weight was not included in the cost study since costs of low birth weight are heterogeneous and not available. However, it is retained in the epidemiological analysis presented here.

**Table B. Characteristics of diseases modelled for each pollutant**

|  | Duration | Terminal | Age category | Pollutant | |
| --- | --- | --- | --- | --- | --- |
|  |  |  |  | **NO2** | **PM2.5** |
| *Respiratory outcomes* | | | | | |
| Asthma (children) | Chronic | Yes | Child | X | X |
| Asthma (adults) | Chronic | Yes | Adult | X |  |
| COPD | Chronic | Yes | Adult |  | X |
| *Cardiovascular outcomes* | | | | | |
| CHD | Chronic | Yes | Adult |  | X |
| Stroke | Chronic | Yes | Adult |  | X |
| Diabetes | Chronic | No | Adult | X | X |
| *Cancer and other outcomes* | | | | | |
| Dementia | Chronic | Yes | Adult | X |  |
| Low birth weight | Acute | No | Adult | X | X |
| Lung cancer | Chronic | Yes | Adult | X | X |

## Exposure response estimates

We searched all relevant and latest reports from Committee on the Medical Effects of Air Pollutants (COMEAP), Environmental Protection Agency (EPA) International Science Assessments [4], World Health Organization (WHO) for long-term effect estimates of exposure to the two pollutants on chronic diseases, focusing on respiratory and cardiovascular disease, and lung cancer. We also conducted PubMed searches for associations with long-term effects by disease group, using a validated and published search strategy [5], see example Pubmed searches:

**COPD:**

(((("air pollution"[Title/Abstract] OR "particulate matter"[Title/Abstract] OR PM[Title/Abstract] OR "nitrogen dioxide"[Title/Abstract] OR "NO2"[Title/Abstract])) AND (COPD OR "chronic obstructive pulmonary disease" OR "chronic bronchitis" OR emphysema)) AND systematic[sb] AND "last 5 years"[PDat])

**Cardiovascular outcomes:**

(("air pollution"[Title/Abstract] OR ozone[Title/Abstract] OR "particulate matter"[Title/Abstract] OR PM[Title/Abstract] OR "nitrogen dioxide"[Title/Abstract] OR "NO2"[Title/Abstract])) AND (CVD[Title/Abstract] OR cardiovascular[Title/Abstract] OR stroke[Title/Abstract] OR cerebrovascular[Title/Abstract] OR "blood pressure"[Title/Abstract] OR hypertension[Title/Abstract] OR diabetes[Title/Abstract])

Effect estimates were included using the following inclusion exclusion and preference criteria:

**Exposure**: Measured or modelled annual exposure to NO_2_ or PM_2.5_. Short term studies including 24hour, 8 hour concentration data were not extracted.

**Outcome**: Incidence or prevalence of respiratory diseases, including asthma, COPD, bronchitis; cardiovascular diseases, including CHD, stroke, diabetes and lung cancer and emerging diseases related to air pollution, including dementia and low birthweight.

**Source**: COMEAP publications were prioritised, after which systematic reviews and meta-analyses preferred to RCT and single cohort studies, but these latter sources were considered if no systematic reviews and meta-analyses for a given pollutant-outcome pair were identified. The most recent review and meta-analysis was preferred, but the inclusion/exclusion criteria and list of included studies was checked against other reviews to ensure the analysis was as complete and relevant as possible. When cohort and case-control/cross-sectional studies were included in the systematic review, these were included, but estimates from subgroup analyses of longitudinal cohort studies were preferred if available. Estimates from random effects meta-analyses were extracted as between-study heterogeneity is expected in studies of air pollution and health outcomes. When studies presented models adjusting for confounding, the model adjusting for the most variables, was selected.

**Effect estimates**: Effects presented as relative risk ratios (RR), hazard ratios (HR) and odds ratios (OR) were extracted. ORs are considered to approximate RR if the effect is rare (which is not the case for the majority of outcomes included) or when the effect size is small i.e above 20% increased odds [6]. For estimates above OR =1.2, the baseline risk was assessed and was found to range between 2.4 and 13.1%. This would result in an overestimation of the RR by less than 10%, so all ORs were considered to approximate RRs. HRs were also considered to approximate RRs.

Effect estimates were presented for a range of exposure units and were standardised to 10$\mu$g/m^3^ for all pollutants. When presented in parts per billion (ppb), conversion factors from Defra [7] were used to obtain estimates in $\mu$g/m^3^. Where effect estimates are based on PM_10_, a conversion factor of 1.43 was applied to convert the effect estimate to PM_2.5_, as recommended and performed in the a review of the quality of air index [8].

When using the dose-response estimate for effect of NO_2_ on dementia, the effect estimate was for NO_x_ which was converted to NO_2_ using a scaling factor of 0.44. This factor was developed by Anderson et al. based on the ratio that fell midway between the average or roadside vs urban background monitoring sites in London for 2001 (see online supplement number 2) [9].

Communication with COMEAP and steering group members led to the decision to reduce all NO_2_ effect estimates to 40% of the original RR (a reduction by 60%) to adjust for the effect of PM_2.5_ and other pollutants. This represents the mid-point of a range of 25-55% reduction recommended to be applied to unadjusted coefficients to account for the effect of PM_2.5_ and other pollutants [10].

presents features from the studies from which dose-response estimates were obtained.

All diseases except for low birth weight were lifelong, chronic diseases, so once acquired, were prevalent for the duration of an individual’s life (see section on modelling birth weight for further details). Individuals could develop more than one diseases, but these were considered independent of one another. All diseases apart from diabetes and low birth weight were terminal. Epidemiological data on each disease’s incidence, prevalence, mortality and survival and dose-response was collected (see Table C). When a parameter, e.g. Survival was not available from the literature or national statistics, this was computed from other data (as described in appendix 1). Note that low birth weight was removed from the cost analysis since no cost data were available, however it is retained within the epidemiological analysis.

**Table C. Summary of studies identified potential associations between long-term exposure to NO2 and PM2.5 of air pollution on chronic disease**

| Diseases | Incidence | Prevalence | Mortality | Survival | Relative Risk |
| --- | --- | --- | --- | --- | --- |
| Asthma | BLF Asthma Statistics [11] | BLF Asthma Statistics [11] | ONS, Deaths Registrations Summary Statistics, England and Wales, 2015 [3] | Computed from prevalence and mortality | **NO_2_:** Khreis *et al.* 2016 [12]  In children =<6 years : OR 1.08 (1.04; 1.12) per 4$\mu$g/m^3^🡪*Converted to*  *OR 1.212 (1.103; 1.328)* per 10$\mu$g/m^3^🡪*REDUCED by 60%* 🡪 **1.08 (1.01; 1.12) per 10**$\boldsymbol{\mu}$**g/m^3^**  In children >6 years: OR 1.03 (1.00; 1.06) per 4$\mu$g/m^3^🡪*Converted to OR 1.08 (1.00; 1.16)* per 10$\mu$g/m^3^🡪 *REDUCED by 60%* 🡪 **1.03 (1.00; 1.06) per 10**$\boldsymbol{\mu}$**g/m^3^**  Jaquemin *et al.* 2015 [4]  *In adults:* OR 1.10 (0.99;1.21) per 10$\mu$g/m^3^ 🡪*REDUCED by 60%* 🡪 **1.04 (0.996; 1.08*)* per 10**$\boldsymbol{\mu}$**g/m^3^**  **PM_2.5_:** Khreis et al. 2016 [12]  In children >6 years: OR 1.04 (1.02; 1.07) per 1$\mu$g/m^3^ 🡪Converted  OR **1.48 (1.22 ; 1.97) per 10**$\boldsymbol{\mu}$**g/m^3^** |
| COPD | Computed from prevalence and mortality. | PHE modelled estimates, 2008 [13] | ONS, Deaths Registrations Summary Statistics, England and Wales, 2015 [3] | Computed from prevalence and mortality | **PM_2.5_:** COMEAP 2016 [14]  COMEAP recommend using PM _10_ estimate based on Cai et al. 2014 estimate for chronic phlegm in never smokers in sensitivity analyses:  OR 1.32 (1.02; 1.71) per 10µg/m^3^ of PM_10_ 🡪 scale to PM_2.5_ using the conversion factor of PM_2.5_-> PM_10_: 0.7 (or PM_10_ -> PM_2.5_:1.42) recently used in the air quality index, COMEAP: Converted to **1.49 (1.03; 2.14) per 10µg/m^3^ of PM_2.5_** |
| CHD | Smolina et al 2012. Corrected data on incidence and mortality in 2013 [15] | BHF, Cardiovascular Disease Statistics 2014 [16] | ONS, Deaths Registrations Summary Statistics, England and Wales, 2015 [3] | Computed from prevalence and mortality | **PM_2.5_:** Cesaroni et al. 2014 [17]  Estimate used in CAPTOR tool from subgroup analysis of participants with additional information on CVD risk factors:  HR 1.19 (1.01; 1.42) per 5$\mu$g/m^3^🡪 Converted to **1.41 (1.00 - 2.01) per 10**$\boldsymbol{\mu}$**g/m^3^** |
| Diabetes | Personal communication with dr Craig Curry from Cardiff University | National Diabetes Audit 2015-2016[18] | Non-terminal | Non-terminal | **NO_2:_** Eze *et al.* 2015 [19]  RR 1.12 (1.05; 1.19) per 10$\mu$g/m^3^🡪*REDUCED by 60%* 🡪 **1.05 (1.02; 1.07) per 10**$\boldsymbol{\mu}$**g/m^3^**  **PM_2.5_**: Eze et al. 2015 [19]  RR **1.10 (1.02; 1.18) per 10**$\boldsymbol{\mu}$**g/m^3^** |
| Stroke | BHF, stroke statistics 2009 [20] | BHF, Cardiovascular Disease Statistics 2014 [16] | ONS, Deaths Registrations Summary Statistics, England and Wales, 2015 [3] | Computed from prevalence and mortality | **PM_2.5_:** Scheers et al. 2015 [21]  HR 1.064 (1.021; 1.109) per 5$\mu$g/m^3^ 🡪Converted to **1.13 (1.04; 1.23) per 10**$\boldsymbol{\mu}$**g/m^3^** |
| Dementia | Computed from prevalence and mortality | Dementia UK 2014 [22] | ONS, Deaths Registrations Summary Statistics, England and Wales, 2015 [3] | Computed from prevalence and mortality | **NO_2_**: Oudin et al. 2016[23]  HR 1.08 (1.00; 1.16) per 10$\mu$g/m^3^ NOx. Scaling factor: NOx → NO2: 0.44 which was developed by Anderson et al. based on the ratio that fell midway between the average or roadside vs urban background monitoring sites in London for 2001 (see Online Supp 2) [9]  Converted from NOx to NO2:HR 1.03 (1.00; 1.07) 🡪REDUCED by 60% 🡪 **1.01 (1.01; 1.03) per 10**$\boldsymbol{\mu}$**g/m3 of NO2** |
| Low birth weight | ONS Birth Characteristics, 2015 [24] | Considered equivalent to incidence | Non-terminal | Non-terminal | **NO_2_**: Pedersen et al. 2013 [25]  OR 1.09 (1.00; 1.19) per 10$\mu$g/m^3^🡪*REDUCED by 60%* 🡪 **1.04 (1.00; 1.07) per 10**$\boldsymbol{\mu}$**g/m^3^**  **PM_2.5_:** Pedersen et al. 2013 [25]  OR 1.18 (1.06; 1.33) per 5$\mu$g/m^3^🡪Converted OR **1.39 (1.12; 1.77) per 10**$\boldsymbol{\mu}$**g/m^3^:** |
| Lung cancer | CRUK, 2012-14 [26] | Not required in model as model uses incidence | CRUK, 2012-14 [26] | 1, 5 year: ONS, 2010-14 [27]; 10 year: ONS, 2008-12 [28] | **NO_2_**: Hamra et al. 2015 [29]  RR 1.04 (1.01; 1.08) per 10$\mu$g/m^3^🡪*REDUCED by 60%* 🡪 **1.02 (1.00; 1.03) per 10**$\boldsymbol{\mu}$**g/m^3^**  **PM_2.5_:** Hamra et al. 2014 [30]  **RR 1.09 (1.04; 1.14) per 10**$\boldsymbol{\mu}$**g/m^3^** |
| All NO_2_ relative risks reduced by 60% following COMEAP recommendations[10] | | | | | |

### Long term exposure to PM2.5

There is strong evidence for a causal association of long term exposure to PM_2.5_ with CHD [31-33], stroke [31-33], lung cancer [34] and asthma exacerbations [31-33]. There is also increasingly strong evidence that associations with development of asthma in children may be causal [32].

Although there is some evidence of an association between the incidence or prevalence of chronic bronchitis and long-term exposure to air pollution (mainly particulate matter measured as PM_10_) it is not sufficient to infer a causal relationship. COMEAP recommends that only sensitivity calculations are undertaken [35]. There is emerging evidence of significant associations between PM_2.5_ and low birth weight [31, 32], whilst evidence between diabetes and long term exposure to PM_2.5_ has recently begun to emerge. [31, 32]. Table D summarises these relationships.

### Long term exposure to NO2

The evidence for long-term exposure to NO_2_ and health effects is less certain than that for fine particulate matter. It is possible that, to some extent, NO_2_ acts as a marker of the effects of other traffic-related pollutants. The evidence has, however, strengthened in recent years and COMEAP note that the epidemiological and mechanistic evidence suggest that it would be sensible to regard NO_2_ as causing some of the health impact found to be associated with it in epidemiological studies [36].

There is strong evidence of causal associations of exacerbations of asthma [37] and NO_2._ There is increasing evidence that the associations of long term exposure to NO_2_ with development of asthma in children may be causal [32, 37].

The evidence between NO_2_ exposure and Diabetes [31, 32, 37], low birth weight [32, 37] and dementia [32] has recently begun to emerge. Compared to children, there is less evidence that asthma in adults is associated with NO_2_ [31, 37] and lung cancer studies have revealed associations with NO_2_ but the mode of action by which NO_2_ could directly cause cancer is unclear. It may be acting as a marker for other pollutants [37]. Table Dsummarises these relationships.

**Table D. Summary of established causal relationships ('strong evidence') and associations ('weaker evidence') for PM2.5 and NO2**

|  | **Long term exposure to PM_2.5_** | **Long term exposure to NO_2_** |
| --- | --- | --- |
| Stronger evidence suggestive for a causal association | Coronary heart disease  Stroke  Lung cancer  Asthma (children) | Asthma (children) |
| Evidence less certain or emerging evidence of associations | Chronic Obstructive Pulmonary Disease (as chronic bronchitis)  Diabetes  Low birth weight | Asthma (adults)  Diabetes  Lung cancer  Low birth weight  Dementia |

## Incidence, Prevalence, Mortality data by disease

### Asthma

**Table E. Asthma epidemiological data (per 100,000 population)**

| Incidence | | Prevalence | | Mortality | | |
| --- | --- | --- | --- | --- | --- | --- |
| BLF Asthma Statistics[11] | | BLF Asthma Statistics[11] | | ONS 2015 [3] | | |
| Data sourced from the THIN Database, ICD codes unclear | | Data sourced from the THIN Database, ICD codes unclear | | ICD 10: J45-J46 | | |
| Age group | Both genders | Age group | Both genders | Age group | Male | Female |
| 0-5 | 929.0 | 0-5 | 3114.0 | <1 | 0.0 | 0.0 |
|  |  |  |  | 1-4 | 0.1 | 0.0 |
| 6-10 | 561.0 | 6-10 | 10079.0 | 5-14 | 0.2 | 0.2 |
| 11-15 | 356.0 | 11-15 | 15899.0 |  |  |  |
| 16-20 | 170.0 | 16-20 | 20180.0 | 15-24 | 0.2 | 0.2 |
| 21-30 | 150.0 | 21-30 | 17306.0 |  |  |  |
|  |  |  |  | 25-34 | 0.2 | 0.2 |
| 31-40 | 180.0 | 31-40 | 13286.0 |  |  |  |
|  |  |  |  | 35-44 | 0.4 | 0.3 |
| 41-50 | 201.0 | 41-50 | 11751.0 |  |  |  |
|  |  |  |  | 45-54 | 0.5 | 1.0 |
| 51-60 | 204.0 | 51-60 | 10903.0 |  |  |  |
|  |  |  |  | 55-64 | 1.1 | 1.4 |
| 61-70 | 231.0 | 61-70 | 10848.0 |  |  |  |
|  |  |  |  | 65-74 | 1.5 | 2.9 |
| 71-80 | 194.0 | 71-80 | 11526.0 |  |  |  |
|  |  |  |  | 75-84 | 5.2 | 12.3 |
| 81+ | 111.0 | 81+ | 10135.0 | 85+ | 32.3 | 56.0 |

### Chronic obstructive pulmonary disease (COPD)

COPD incidence was estimated from prevalence and mortality data, – see S1 Text section *Approximating missing disease statistics* for methods.

**Table F. COPD epidemiological data (per 100,000 population)**

| Incidence | | | Prevalence | | | Mortality | | |
| --- | --- | --- | --- | --- | --- | --- | --- | --- |
| UKHF Derived estimates | | | PHE Modelled estimates (2008) [13] | | | ONS 2015 [3] | | |
| Computed from Prevalence and Mortality | | | COPD based on FEV1 measurements in the Health Survey for England 2001 using British Thoracic Society criteria | | | ICD 10: J40-J44 | | |
| Age group | Male | Female | Age group | Male | Female | Age group | Male | Female |
| 0-4 | 1.2 | 0.0 | 0-4 | 4.2 | 5.9 | <1 | 0.0 | 0.0 |
|  |  |  |  |  |  | 1-4 | 0.0 | 0.0 |
| 5-9 | 2.6 | 3.4 | 5-9 | 10.4 | 6.0 | 5-9 | 0.0 | 0.0 |
| 10-14 | 2.1 | 1.6 | 10-14 | 23.2 | 23.1 |  |  |  |
| 15-19 | 0.0 | 0.0 | 15-19 | 33.7 | 31.3 | 15-24 | 0.0 | 0.0 |
| 20-24 | 1.8 | 1.3 | 20-24 | 27.6 | 12.9 |  |  |  |
| 25-29 | 3.4 | 3.4 | 25-29 | 36.8 | 19.6 | 25-34 | 0.1 | 0.1 |
| 30-34 | 7.9 | 9.8 | 30-34 | 54.0 | 36.5 |  |  |  |
| 35-39 | 25.0 | 28.2 | 35-39 | 93.3 | 85.5 | 35-44 | 1.0 | 0.5 |
| 40-44 | 52.0 | 60.4 | 40-44 | 218.0 | 226.3 |  |  |  |
| 45-49 | 106.0 | 121.2 | 45-49 | 477.1 | 527.0 | 45-54 | 6.4 | 4.4 |
| 50-54 | 205.2 | 174.5 | 50-54 | 1003.6 | 1128.5 |  |  |  |
| 55-59 | 298.0 | 235.4 | 55-59 | 2015.4 | 1988.1 | 55-64 | 32.0 | 28.2 |
| 60-64 | 491.9 | 326.9 | 60-64 | 3466.8 | 3136.4 |  |  |  |
| 65-69 | 437.5 | 216.8 | 65-69 | 5817.7 | 4709.4 | 65-74 | 126.6 | 101.4 |
| 70-74 | 334.8 | 228.0 | 70-74 | 7860.1 | 5737.9 |  |  |  |
| 75-79 | 263.4 | 0.0 | 75-79 | 9392.4 | 6807.6 | 75-84 | 367.7 | 283.2 |
| 80-84 | 0.0 | 0.0 | 80-84 | 10579.6 | 6783.3 |  |  |  |
| 85+ | 0.0 | 0.0 | 85+ | 9857.29 | 5300.6 | 85+ | 939.45 | 558.4 |

### Coronary heart disease (CHD)

**Table G. CHD epidemiological data (per 100,000 population)**

| Incidence | | | Prevalence | | | Mortality | | |
| --- | --- | --- | --- | --- | --- | --- | --- | --- |
| Smolina et al. 2012[15] | | | BHF CVD Stats 2014[16] | | | ONS 2015 [3] | | |
| ICD 10: I21-I22 | | | ICD 10: I21 | | | ICD 10: I21-I22 | | |
| Age group | Male | Female | Age group | Male | Female | Age group | Male | Female |
|  |  |  | 0-44 | 60.0 | 30.0 | <1 | 0.0 | 0.3 |
|  |  |  |  |  |  | 1-4 | 0.0 | 0.0 |
|  |  |  |  |  |  | 5-9 | 0.0 | 0.0 |
|  |  |  |  |  |  | 15-24 | 0.1 | 0.0 |
|  |  |  |  |  |  | 25-34 | 0.8 | 0.2 |
| 30-54 | 88.1 | 21.2 |  |  |  |  |  |  |
|  |  |  |  |  |  | 35-44 | 4.9 | 1.4 |
|  |  |  | 45-54 | 1070.0 | 430.0 | 45-54 | 21.2 | 5.2 |
| 55-64 | 317.0 | 90.3 | 55-64 | 4510.0 | 1240.0 | 55-64 | 52.2 | 14.2 |
| 65-74 | 533.0 | 237.0 | 65-74 | 8660.0 | 2960.0 | 65-74 | 109.4 | 44.6 |
| 75-84 | 1017.0 | 597.0 | 75+ | 14780.0 | 6960.0 | 75-84 | 281.0 | 146.0 |
| 85+ | 1987.0 | 1395.0 |  |  |  | 85+ | 692.0 | 454.7 |

### Stroke

**Table H. Stroke epidemiological data (per 100,000 population)**

| Incidence | | | Prevalence | | | Mortality | | |
| --- | --- | --- | --- | --- | --- | --- | --- | --- |
| BHF Stroke Statistics 2009[20] | | | BHF CVD Stats 2014[16] | | | ONS 2015[3] | | |
| Based on general practice records, ICD codes not given | | | ICD 10: I60-I69 | | | ICD: I60-I64 | | |
| Age group | Male | Female | Age group | Male | Female | Age group | Male | Female |
| 0-44 | 7.0 | 6.0 | 0-44 | 110.0 | 110.0 | <1 | 0.3 | 0.0 |
|  |  |  |  |  |  | 1-4 | 0.0 | 0.1 |
|  |  |  |  |  |  | 5-9 | 0.1 | 0.2 |
|  |  |  |  |  |  | 15-24 | 0.5 | 0.3 |
|  |  |  |  |  |  | 25-34 | 0.9 | 1.0 |
|  |  |  |  |  |  | 35-44 | 3.3 | 2.9 |
| 45-64 | 114.0 | 69.0 | 45-54 | 890.0 | 790.0 | 45-54 | 9.9 | 8.0 |
|  |  |  | 55-64 | 2690.0 | 1960.0 | 55-64 | 26.1 | 19.1 |
| 65-74 | 393.0 | 275.0 | 65-74 | 6400.0 | 4390.0 | 65-74 | 67.7 | 56.7 |
| 75+ | 794.0 | 879.0 | 75+ | 14890.0 | 12430.0 | 75-84 | 284.6 | 260.5 |
|  |  |  |  |  |  | 85+ | 915.9 | 1031.7 |

### Diabetes Type 2

**Table I. Diabetes incidence and prevalence estimates (per 100,000 population)**

| Incidence | | | Prevalence | | | Mortality | | |
| --- | --- | --- | --- | --- | --- | --- | --- | --- |
| Personal communication with Dr Curry from Cardiff University [38] | | | National Diabetes Audit 2015-2016[18] | | |  |  |  |
| ICD 10 codes unknown | | | ICD 10 codes unknown | | |  |  |  |
| Age group | Male | Female | Age group | Male | Female | Age group | Male | Female |
| 0-4 | 56 | 53 | 0-4 | 1.999 | 2.727 | Non terminal | | |
| 5-9 | 34 | 42 | 5-9 | 6.681 | 6.372 |  |  |  |
| 10-14 | 43 | 40 | 10-14 | 15 | 19.285 |  |  |  |
| 15-19 | 83 | 107 | 15-19 | 41.744 | 64.613 |  |  |  |
| 20-24 | 75 | 145 | 20-24 | 85.329 | 160.621 |  |  |  |
| 25-29 | 101 | 226 | 25-29 | 202.748 | 352.739 |  |  |  |
| 30-34 | 150 | 242 | 30-34 | 561.584 | 684.461 |  |  |  |
| 35-39 | 240 | 263 | 35-39 | 1361.296 | 1249.819 |  |  |  |
| 40-44 | 355 | 333 | 40-44 | 2617.251 | 1898.323 |  |  |  |
| 45-49 | 561 | 482 | 45-49 | 4338.317 | 2858.298 |  |  |  |
| 50-54 | 820 | 636 | 50-54 | 6451.945 | 4227.206 |  |  |  |
| 55-59 | 1068 | 847 | 55-59 | 9371.893 | 6188.7 |  |  |  |
| 60-64 | 1316 | 965 | 60-64 | 11825.85 | 7780.135 |  |  |  |
| 65-69 | 1516 | 1234 | 65-69 | 13621.13 | 9047.041 |  |  |  |
| 70-74 | 1763 | 1378 | 70-74 | 16010.86 | 11196.63 |  |  |  |
| 75-79 | 1677 | 1483 | 75-79 | 18065.24 | 13559.67 |  |  |  |
| 80-84 | 1645 | 1336 | 80-84 | 18464.43 | 14217.44 |  |  |  |
| 85-89 | 1300 | 1169 | 85+ | 15210.91 | 11513.66 |  |  |  |
| 90+ | 546 | 440 |  |  |  |  |  |  |

### Dementia

Dementia incidence was estimated from prevalence and mortality data, – see S1 Text section *Approximating missing disease statistics* for methods.

In order to align the mortality age groups with the prevalence age groups, prevalence in age groups 85-89, 90-94 and 95+ were pooled . The pooling was weighted based on the number of cases in each age group, itself a function of population in each age group.

**Table J Dementia epidemiological data (per 100,000 population)**

| Incidence | | | Prevalence | | | Mortality | | |
| --- | --- | --- | --- | --- | --- | --- | --- | --- |
| UKHF Derived estimate | | | Dementia UK 2014 [22] | | | ONS 2015 [3] | | |
| Computed from Prevalence and Mortality | | | ICD 10: F00-F03 | | | ICD 10: F01,F03 | | |
| Age group | Male | Female | Age group | Male | Female | Age group | Male | Female |
| 0-59 | 0 | 0 |  |  |  | <1 |  |  |
|  |  |  |  |  |  | 1-4 |  |  |
|  |  |  |  |  |  | 5-14 |  |  |
|  |  |  |  |  |  | 15-24 |  |  |
|  |  |  |  |  |  | 25-34 |  |  |
|  |  |  |  |  |  | 35-44 |  |  |
|  |  |  |  |  |  | 45-54 | 0.1 | 0.2 |
|  |  |  |  |  |  |  |  |  |
|  |  |  |  |  |  | 55-64 | 2.2 | 1.9 |
| 60-64 | 488.06 | 515.88 | 60-64 | 900 | 900 |  |  |  |
| 65-69 | 304.79 | 492.8 | 65-69 | 1500 | 1800 | 65-74 | 30.4 | 26.9 |
| 70-74 | 1278.18 | 1031.38 | 70-74 | 3100 | 3000 |  |  |  |
| 75-79 | 1422.62 | 2875.31 | 75-79 | 5300 | 6600 | 75-84 | 349.9 | 360.4 |
| 80-84 | 4042.55 | 2985.94 | 80-84 | 10300 | 11700 |  |  |  |
| 85-89 | 3363.16 | 8573.13 | 85-89 | 15100 | 20200 | 85+ | 2025.7 | 2686.9 |
|  |  |  | 90-94 | 22600 | 33000 |  |  |  |
|  |  |  | 95+ | 28800 | 44200 |  |  |  |

### Low birth weight

The outcome of low birth weight is related to several health outcomes throughout the life course, however, these downstream consequences was not be modelled in this project. Low birth weight is modelled as an outcome of the mother. Breakdown by maternal age was not available, and rates of 7% of all live births being low birth weight have been stable since 2011[24]. Low birth weight was the only disease not modelled as a lifelong, chronic disease. Prevalence of low birth weight in 1 year is considered equivalent to incidence, as low birth weight is considered an acute event occurring only in a given year.

**Table K. Low birth weight epidemiological data (per 100,000 population)**

| Incidence | | | Prevalence | | | Mortality | | |
| --- | --- | --- | --- | --- | --- | --- | --- | --- |
| ONS 2015[24] | | |  |  |  |  |  |  |
| ICD 10: P07.1 | | |  |  |  |  |  |  |
| Age grp | Male | Female | Age grp | Male | Female | Age grp | Male | Female |
| 16-59 | NA | 7000 | Prevalence of low birth weight in 1 year is considered equivalent to incidence. | | | Not applicable (non terminal) | | |

### Lung cancer

Prevalence data was not available on lung cancer data, but the model does not require the input of prevalence, only of incidence, so this parameter was not required.

**Table L. Lung cancer epidemiological data (per 100,000 population)**

| Incidence | | | Prevalence | | | Mortality | | |
| --- | --- | --- | --- | --- | --- | --- | --- | --- |
| CRUK 2012-14 [26] | | | Prevalence is not a required input into the model | | | CRUK 2012-14 [26] | | |
| ICD 10: C33-C34 | | | N/A |  |  | ICD 10: C33-C34 | | |
| Age group | Male | Female | Age group | Male | Female | Age group | Male | Female |
| 0-4 | 0.1 | 0.0 |  |  |  | 0-5 | 0.0 | 0.0 |
|  |  |  |  |  |  |  |  |  |
| 5-9 | 0.0 | 0.0 |  |  |  | 5-9 | 0.0 | 0.0 |
| 10-14 | 0.0 | 0.0 |  |  |  | 10-14 | 0.0 | 0.0 |
| 15-19 | 0.1 | 0.1 |  |  |  | 15-19 | 0.0 | 0.0 |
| 20-24 | 0.3 | 0.3 |  |  |  | 20-24 | 0.1 | 0.0 |
| 25-29 | 0.5 | 0.6 |  |  |  | 25-29 | 0.1 | 0.1 |
| 30-34 | 0.9 | 1.3 |  |  |  | 30-34 | 0.4 | 0.5 |
| 35-39 | 2.3 | 2.4 |  |  |  | 35-39 | 1.4 | 1.1 |
| 40-44 | 6.7 | 5.6 |  |  |  | 40-44 | 4.3 | 3.0 |
| 45-49 | 15.9 | 15.1 |  |  |  | 45-49 | 10.9 | 9.6 |
| 50-54 | 36.0 | 34.0 |  |  |  | 50-54 | 24.9 | 21.6 |
| 55-59 | 80.0 | 72.7 |  |  |  | 55-59 | 58.1 | 48.0 |
| 60-64 | 151.5 | 126.2 |  |  |  | 60-64 | 109.8 | 84.1 |
| 65-69 | 239.5 | 190.5 |  |  |  | 65-69 | 176.2 | 131.1 |
| 70-74 | 367.9 | 264.4 |  |  |  | 70-74 | 279.9 | 190.9 |
| 75-79 | 463.2 | 305.5 |  |  |  | 75-79 | 367.8 | 234.1 |
| 80-84 | 538.1 | 337.6 |  |  |  | 80-84 | 453.6 | 283.6 |
| 85-89 | 608.7 | 339.1 |  |  |  | 85-89 | 564.4 | 309.4 |
| 90+ | 542.0 | 253.7 |  |  |  | 90+ | 521.2 | 245.6 |

## Survival data

Survival statistics for CHD, COPD, Stroke and dementia were not identified in the literature. We modelled these using prevalence and mortality data, – see S1 Text section *Approximating missing disease statistics* for methods.

### Asthma

**Table M. Probability of 1, 5 and 1 year survival computed from prevalence and mortality data for Asthma.**

| Age | Survival probability – 1 year | | Survival probability – 5 years | | Survival probability – 10 years | |
| --- | --- | --- | --- | --- | --- | --- |
|  | **M** | **F** | **M** | **F** | **M** | **F** |
| 0-74 | 1.000 | 1.000 | 1.000 | 1.000 | 1.000 | 1.000 |
| 75-84 | 1.000 | 0.999 | 1.000 | 0.999 | 1.000 | 0.999 |
| 85-94 | 0.998 | 0.997 | 0.998 | 0.997 | 0.998 | 0.997 |
| 95-107 | 0.999 | 0.997 | 0.999 | 0.997 | 0.999 | 0.997 |
| 108+ | 0.999 | 0.998 | 0.999 | 0.998 | 0.999 | 0.998 |

### CHD and COPD

**Table N. Probability of 1, 5 and 1 year survival computed from prevalence and mortality data for Coronary Heart Disease and Chronic Obstructive Pulmonary Disease.**

| Age | CHD | | | | | | COPD | | | | | |
| --- | --- | --- | --- | --- | --- | --- | --- | --- | --- | --- | --- | --- |
|  | **Survival probability – 1 year** | | **Survival probability – 5 year** | | **Survival probability – 10 year** | | **Survival probability – 1 year** | | **Survival probability – 5 year** | | **Survival probability – 10 year** | |
|  | **M** | **F** | **M** | **F** | **M** | **F** | **M** | **F** | **M** | **F** | **M** | **F** |
| 1-5 | 1.000 | 1.000 | 1.000 | 1.000 | 1.000 | 1.000 | 1.000 | 1.000 | 1.000 | 1.000 | 1.000 | 1.000 |
| 6 | 0.500 | 1.000 | 0.500 | 1.000 | 0.500 | 1.000 | 1.000 | 1.000 | 1.000 | 1.000 | 1.000 | 1.000 |
| 7 | 0.667 | 1.000 | 0.667 | 1.000 | 0.667 | 1.000 | 1.000 | 1.000 | 1.000 | 1.000 | 1.000 | 1.000 |
| 8 | 0.750 | 1.000 | 0.750 | 1.000 | 0.750 | 1.000 | 1.000 | 1.000 | 1.000 | 1.000 | 1.000 | 1.000 |
| 9 | 0.800 | 1.000 | 0.800 | 1.000 | 0.800 | 1.000 | 1.000 | 1.000 | 1.000 | 1.000 | 1.000 | 1.000 |
| 10 | 0.833 | 1.000 | 0.833 | 1.000 | 0.833 | 1.000 | 1.000 | 1.000 | 1.000 | 1.000 | 1.000 | 1.000 |
| 11 | 0.857 | 1.000 | 0.857 | 1.000 | 0.857 | 1.000 | 1.000 | 1.000 | 1.000 | 1.000 | 1.000 | 1.000 |
| 12 | 0.875 | 1.000 | 0.875 | 1.000 | 0.875 | 1.000 | 1.000 | 1.000 | 1.000 | 1.000 | 1.000 | 1.000 |
| 13 | 0.889 | 1.000 | 0.889 | 1.000 | 0.889 | 1.000 | 1.000 | 1.000 | 1.000 | 1.000 | 1.000 | 1.000 |
| 14 | 0.900 | 1.000 | 0.900 | 1.000 | 0.900 | 1.000 | 1.000 | 1.000 | 1.000 | 1.000 | 1.000 | 1.000 |
| 15 | 1.000 | 0.000 | 1.000 | 0.000 | 1.000 | 0.000 | 1.000 | 1.000 | 1.000 | 1.000 | 1.000 | 1.000 |
| 16 | 1.000 | 0.500 | 1.000 | 0.500 | 1.000 | 0.500 | 1.000 | 1.000 | 1.000 | 1.000 | 1.000 | 1.000 |
| 17 | 1.000 | 0.667 | 1.000 | 0.667 | 1.000 | 0.667 | 1.000 | 1.000 | 1.000 | 1.000 | 1.000 | 1.000 |
| 18 | 1.000 | 0.750 | 1.000 | 0.750 | 1.000 | 0.750 | 1.000 | 1.000 | 1.000 | 1.000 | 1.000 | 1.000 |
| 19 | 1.000 | 0.800 | 1.000 | 0.800 | 1.000 | 0.800 | 1.000 | 1.000 | 1.000 | 1.000 | 1.000 | 1.000 |
| 20 | 1.000 | 0.833 | 1.000 | 0.833 | 1.000 | 0.833 | 1.000 | 1.000 | 1.000 | 1.000 | 1.000 | 1.000 |
| 21 | 1.000 | 0.857 | 1.000 | 0.857 | 1.000 | 0.857 | 1.000 | 1.000 | 1.000 | 1.000 | 1.000 | 1.000 |
| 22 | 1.000 | 0.875 | 1.000 | 0.875 | 1.000 | 0.875 | 1.000 | 1.000 | 1.000 | 1.000 | 1.000 | 1.000 |
| 23 | 1.000 | 0.889 | 1.000 | 0.889 | 1.000 | 0.889 | 1.000 | 1.000 | 1.000 | 1.000 | 1.000 | 1.000 |
| 24 | 1.000 | 0.900 | 1.000 | 0.900 | 1.000 | 0.900 | 1.000 | 1.000 | 1.000 | 1.000 | 1.000 | 1.000 |
| 25 | 0.792 | 0.849 | 0.792 | 0.849 | 0.792 | 0.849 | 1.000 | 1.000 | 1.000 | 1.000 | 1.000 | 1.000 |
| 26 | 0.828 | 0.868 | 0.828 | 0.868 | 0.828 | 0.868 | 1.000 | 1.000 | 1.000 | 1.000 | 1.000 | 1.000 |
| 27 | 0.853 | 0.884 | 0.853 | 0.884 | 0.853 | 0.884 | 1.000 | 1.000 | 1.000 | 1.000 | 1.000 | 1.000 |
| 28 | 0.872 | 0.896 | 0.872 | 0.896 | 0.872 | 0.896 | 1.000 | 1.000 | 1.000 | 1.000 | 1.000 | 1.000 |
| 29 | 0.887 | 0.906 | 0.887 | 0.906 | 0.887 | 0.906 | 1.000 | 1.000 | 1.000 | 1.000 | 1.000 | 1.000 |
| 30 | 0.898 | 0.914 | 0.898 | 0.914 | 0.898 | 0.914 | 0.991 | 0.992 | 0.991 | 0.992 | 0.991 | 0.992 |
| 31 | 0.908 | 0.921 | 0.908 | 0.921 | 0.908 | 0.921 | 0.996 | 0.996 | 0.996 | 0.996 | 0.996 | 0.996 |
| 32 | 0.915 | 0.927 | 0.915 | 0.927 | 0.915 | 0.927 | 0.997 | 0.997 | 0.997 | 0.997 | 0.997 | 0.997 |
| 33 | 0.922 | 0.932 | 0.922 | 0.932 | 0.922 | 0.932 | 0.998 | 0.998 | 0.998 | 0.998 | 0.998 | 0.998 |
| 34 | 0.928 | 0.936 | 0.928 | 0.936 | 0.928 | 0.936 | 0.998 | 0.998 | 0.998 | 0.998 | 0.998 | 0.998 |
| 35 | 0.507 | 0.595 | 0.507 | 0.595 | 0.507 | 0.595 | 0.991 | 0.989 | 0.991 | 0.989 | 0.991 | 0.989 |
| 36 | 0.670 | 0.712 | 0.670 | 0.712 | 0.670 | 0.712 | 0.992 | 0.991 | 0.992 | 0.991 | 0.992 | 0.991 |
| 37 | 0.752 | 0.776 | 0.752 | 0.776 | 0.752 | 0.776 | 0.993 | 0.992 | 0.993 | 0.992 | 0.993 | 0.992 |
| 38 | 0.801 | 0.817 | 0.801 | 0.817 | 0.801 | 0.817 | 0.994 | 0.993 | 0.994 | 0.993 | 0.994 | 0.993 |
| 39 | 0.834 | 0.845 | 0.834 | 0.845 | 0.834 | 0.845 | 0.995 | 0.993 | 0.995 | 0.993 | 0.995 | 0.993 |
| 40 | 0.858 | 0.866 | 0.858 | 0.866 | 0.858 | 0.866 | 0.995 | 0.994 | 0.995 | 0.994 | 0.995 | 0.994 |
| 41 | 0.875 | 0.882 | 0.875 | 0.882 | 0.875 | 0.882 | 0.995 | 0.995 | 0.995 | 0.995 | 0.995 | 0.995 |
| 42 | 0.889 | 0.894 | 0.889 | 0.894 | 0.889 | 0.894 | 0.996 | 0.995 | 0.996 | 0.995 | 0.996 | 0.995 |
| 43 | 0.900 | 0.905 | 0.900 | 0.905 | 0.900 | 0.905 | 0.996 | 0.995 | 0.996 | 0.995 | 0.996 | 0.995 |
| 44 | 0.909 | 0.913 | 0.909 | 0.913 | 0.909 | 0.913 | 0.996 | 0.996 | 0.996 | 0.996 | 0.996 | 0.996 |
| 45 | 0.637 | 0.583 | 0.637 | 0.583 | 0.637 | 0.583 | 0.985 | 0.985 | 0.985 | 0.985 | 0.985 | 0.985 |
| 46 | 0.734 | 0.706 | 0.734 | 0.706 | 0.734 | 0.706 | 0.986 | 0.986 | 0.986 | 0.986 | 0.986 | 0.986 |
| 47 | 0.790 | 0.773 | 0.790 | 0.773 | 0.790 | 0.773 | 0.987 | 0.986 | 0.987 | 0.986 | 0.987 | 0.986 |
| 48 | 0.826 | 0.815 | 0.826 | 0.815 | 0.826 | 0.815 | 0.987 | 0.987 | 0.987 | 0.987 | 0.987 | 0.987 |
| 49 | 0.852 | 0.844 | 0.852 | 0.844 | 0.852 | 0.844 | 0.988 | 0.988 | 0.988 | 0.988 | 0.988 | 0.988 |
| 50 | 0.871 | 0.865 | 0.871 | 0.865 | 0.871 | 0.865 | 0.989 | 0.988 | 0.989 | 0.988 | 0.989 | 0.988 |
| 51 | 0.886 | 0.881 | 0.886 | 0.881 | 0.886 | 0.881 | 0.989 | 0.989 | 0.989 | 0.989 | 0.989 | 0.989 |
| 52 | 0.898 | 0.894 | 0.898 | 0.894 | 0.898 | 0.894 | 0.990 | 0.989 | 0.990 | 0.989 | 0.990 | 0.989 |
| 53 | 0.907 | 0.904 | 0.907 | 0.904 | 0.907 | 0.904 | 0.990 | 0.990 | 0.990 | 0.990 | 0.990 | 0.990 |
| 54 | 0.915 | 0.912 | 0.915 | 0.912 | 0.915 | 0.912 | 0.990 | 0.990 | 0.990 | 0.990 | 0.990 | 0.990 |
| 55 | 0.702 | 0.638 | 0.702 | 0.638 | 0.702 | 0.638 | 0.979 | 0.977 | 0.979 | 0.977 | 0.979 | 0.977 |
| 56 | 0.771 | 0.734 | 0.771 | 0.734 | 0.771 | 0.734 | 0.981 | 0.980 | 0.981 | 0.980 | 0.981 | 0.980 |
| 57 | 0.813 | 0.790 | 0.813 | 0.790 | 0.813 | 0.790 | 0.983 | 0.982 | 0.983 | 0.982 | 0.983 | 0.982 |
| 58 | 0.843 | 0.827 | 0.843 | 0.827 | 0.843 | 0.827 | 0.985 | 0.984 | 0.985 | 0.984 | 0.985 | 0.984 |
| 59 | 0.864 | 0.852 | 0.864 | 0.852 | 0.864 | 0.852 | 0.986 | 0.986 | 0.986 | 0.986 | 0.986 | 0.986 |
| 60 | 0.880 | 0.871 | 0.880 | 0.871 | 0.880 | 0.871 | 0.987 | 0.987 | 0.987 | 0.987 | 0.987 | 0.987 |
| 61 | 0.893 | 0.886 | 0.893 | 0.886 | 0.893 | 0.886 | 0.988 | 0.988 | 0.988 | 0.988 | 0.988 | 0.988 |
| 62 | 0.903 | 0.898 | 0.903 | 0.898 | 0.903 | 0.898 | 0.989 | 0.989 | 0.989 | 0.989 | 0.989 | 0.989 |
| 63 | 0.912 | 0.907 | 0.912 | 0.907 | 0.912 | 0.907 | 0.989 | 0.989 | 0.989 | 0.989 | 0.989 | 0.989 |
| 64 | 0.919 | 0.915 | 0.919 | 0.915 | 0.919 | 0.915 | 0.990 | 0.990 | 0.990 | 0.990 | 0.990 | 0.990 |
| 65 | 0.757 | 0.766 | 0.757 | 0.766 | 0.757 | 0.766 | 0.981 | 0.973 | 0.981 | 0.973 | 0.981 | 0.973 |
| 66 | 0.804 | 0.810 | 0.804 | 0.810 | 0.804 | 0.810 | 0.983 | 0.976 | 0.983 | 0.976 | 0.983 | 0.976 |
| 67 | 0.836 | 0.840 | 0.836 | 0.840 | 0.836 | 0.840 | 0.984 | 0.979 | 0.984 | 0.979 | 0.984 | 0.979 |
| 68 | 0.859 | 0.862 | 0.859 | 0.862 | 0.859 | 0.862 | 0.985 | 0.981 | 0.985 | 0.981 | 0.985 | 0.981 |
| 69 | 0.876 | 0.879 | 0.876 | 0.879 | 0.876 | 0.879 | 0.986 | 0.983 | 0.986 | 0.983 | 0.986 | 0.983 |
| 70 | 0.890 | 0.892 | 0.890 | 0.892 | 0.890 | 0.892 | 0.987 | 0.984 | 0.987 | 0.984 | 0.987 | 0.984 |
| 71 | 0.901 | 0.902 | 0.901 | 0.902 | 0.901 | 0.902 | 0.988 | 0.985 | 0.988 | 0.985 | 0.988 | 0.985 |
| 72 | 0.910 | 0.911 | 0.910 | 0.911 | 0.910 | 0.911 | 0.988 | 0.986 | 0.988 | 0.986 | 0.988 | 0.986 |
| 73 | 0.917 | 0.918 | 0.917 | 0.918 | 0.917 | 0.918 | 0.989 | 0.987 | 0.989 | 0.987 | 0.989 | 0.987 |
| 74 | 0.923 | 0.924 | 0.923 | 0.924 | 0.923 | 0.924 | 0.989 | 0.988 | 0.989 | 0.988 | 0.989 | 0.988 |
| 75 | 0.817 | 0.825 | 0.817 | 0.825 | 0.817 | 0.825 | 0.975 | 0.966 | 0.975 | 0.966 | 0.975 | 0.966 |
| 76 | 0.845 | 0.851 | 0.845 | 0.851 | 0.845 | 0.851 | 0.977 | 0.970 | 0.977 | 0.970 | 0.977 | 0.970 |
| 77 | 0.865 | 0.870 | 0.865 | 0.870 | 0.865 | 0.870 | 0.978 | 0.973 | 0.978 | 0.973 | 0.978 | 0.973 |
| 78 | 0.881 | 0.884 | 0.881 | 0.884 | 0.881 | 0.884 | 0.980 | 0.976 | 0.980 | 0.976 | 0.980 | 0.976 |
| 79 | 0.893 | 0.896 | 0.893 | 0.896 | 0.893 | 0.896 | 0.981 | 0.978 | 0.981 | 0.978 | 0.981 | 0.978 |
| 80 | 0.903 | 0.906 | 0.903 | 0.906 | 0.903 | 0.906 | 0.982 | 0.980 | 0.982 | 0.980 | 0.982 | 0.980 |
| 81 | 0.911 | 0.914 | 0.911 | 0.914 | 0.911 | 0.914 | 0.983 | 0.981 | 0.983 | 0.981 | 0.983 | 0.981 |
| 82 | 0.918 | 0.920 | 0.918 | 0.920 | 0.918 | 0.920 | 0.984 | 0.982 | 0.984 | 0.982 | 0.984 | 0.982 |
| 83 | 0.924 | 0.926 | 0.924 | 0.926 | 0.924 | 0.926 | 0.984 | 0.983 | 0.984 | 0.983 | 0.984 | 0.983 |
| 84 | 0.929 | 0.931 | 0.929 | 0.931 | 0.929 | 0.931 | 0.985 | 0.984 | 0.985 | 0.984 | 0.985 | 0.984 |
| 85 | 0.846 | 0.879 | 0.846 | 0.879 | 0.846 | 0.879 | 0.966 | 0.957 | 0.966 | 0.957 | 0.966 | 0.957 |
| 86 | 0.865 | 0.892 | 0.865 | 0.892 | 0.865 | 0.892 | 0.969 | 0.962 | 0.969 | 0.962 | 0.969 | 0.962 |
| 87 | 0.880 | 0.902 | 0.880 | 0.902 | 0.880 | 0.902 | 0.971 | 0.965 | 0.971 | 0.965 | 0.971 | 0.965 |
| 88 | 0.892 | 0.910 | 0.892 | 0.910 | 0.892 | 0.910 | 0.973 | 0.968 | 0.973 | 0.968 | 0.973 | 0.968 |
| 89 | 0.902 | 0.917 | 0.902 | 0.917 | 0.902 | 0.917 | 0.974 | 0.971 | 0.974 | 0.971 | 0.974 | 0.971 |
| 90 | 0.910 | 0.923 | 0.910 | 0.923 | 0.910 | 0.923 | 0.975 | 0.973 | 0.975 | 0.973 | 0.975 | 0.973 |
| 91 | 0.917 | 0.928 | 0.917 | 0.928 | 0.917 | 0.928 | 0.977 | 0.975 | 0.977 | 0.975 | 0.977 | 0.975 |
| 92 | 0.922 | 0.933 | 0.922 | 0.933 | 0.922 | 0.933 | 0.978 | 0.976 | 0.978 | 0.976 | 0.978 | 0.976 |
| 93 | 0.927 | 0.937 | 0.927 | 0.937 | 0.927 | 0.937 | 0.979 | 0.977 | 0.979 | 0.977 | 0.979 | 0.977 |
| 94 | 0.932 | 0.940 | 0.932 | 0.940 | 0.932 | 0.940 | 0.979 | 0.979 | 0.979 | 0.979 | 0.979 | 0.979 |
| 95 | 0.936 | 0.943 | 0.936 | 0.943 | 0.936 | 0.943 | 0.980 | 0.980 | 0.980 | 0.980 | 0.980 | 0.980 |
| 96 | 0.939 | 0.946 | 0.939 | 0.946 | 0.939 | 0.946 | 0.981 | 0.981 | 0.981 | 0.981 | 0.981 | 0.981 |
| 97 | 0.942 | 0.948 | 0.942 | 0.948 | 0.942 | 0.948 | 0.982 | 0.981 | 0.982 | 0.981 | 0.982 | 0.981 |
| 98 | 0.945 | 0.951 | 0.945 | 0.951 | 0.945 | 0.951 | 0.982 | 0.982 | 0.982 | 0.982 | 0.982 | 0.982 |
| 99 | 0.947 | 0.953 | 0.947 | 0.953 | 0.947 | 0.953 | 0.983 | 0.983 | 0.983 | 0.983 | 0.983 | 0.983 |
| 100 | 0.949 | 0.955 | 0.949 | 0.955 | 0.949 | 0.955 | 0.983 | 0.984 | 0.983 | 0.984 | 0.983 | 0.984 |
| 101 | 0.951 | 0.956 | 0.951 | 0.956 | 0.951 | 0.956 | 0.984 | 0.984 | 0.984 | 0.984 | 0.984 | 0.984 |
| 102 | 0.953 | 0.958 | 0.953 | 0.958 | 0.953 | 0.958 | 0.984 | 0.985 | 0.984 | 0.985 | 0.984 | 0.985 |
| 103 | 0.955 | 0.960 | 0.955 | 0.960 | 0.955 | 0.960 | 0.985 | 0.985 | 0.985 | 0.985 | 0.985 | 0.985 |
| 104 | 0.956 | 0.961 | 0.956 | 0.961 | 0.956 | 0.961 | 0.985 | 0.986 | 0.985 | 0.986 | 0.985 | 0.986 |
| 105 | 0.958 | 0.962 | 0.958 | 0.962 | 0.958 | 0.962 | 0.985 | 0.986 | 0.985 | 0.986 | 0.985 | 0.986 |
| 106 | 0.959 | 0.963 | 0.959 | 0.963 | 0.959 | 0.963 | 0.986 | 0.986 | 0.986 | 0.986 | 0.986 | 0.986 |
| 107 | 0.960 | 0.964 | 0.960 | 0.964 | 0.960 | 0.964 | 0.986 | 0.987 | 0.986 | 0.987 | 0.986 | 0.987 |
| 108 | 0.962 | 0.965 | 0.962 | 0.965 | 0.962 | 0.965 | 0.986 | 0.987 | 0.986 | 0.987 | 0.986 | 0.987 |
| 109 | 0.963 | 0.966 | 0.963 | 0.966 | 0.963 | 0.966 | 0.986 | 0.987 | 0.986 | 0.987 | 0.986 | 0.987 |
| 109+ | 0.963 | 0.966 | 0.963 | 0.966 | 0.963 | 0.966 | 0.986 | 0.987 | 0.986 | 0.987 | 0.986 | 0.987 |

### Stroke and Dementia

**Table O Probability of 1, 5 and 1 year survival computed from prevalence and mortality data for Stroke and Dementia.**

| Age | Stroke | | | | | | Dementia | | | | | |
| --- | --- | --- | --- | --- | --- | --- | --- | --- | --- | --- | --- | --- |
|  | **Survival probability – 1 year** | | **Survival probability – 5 year** | | **Survival probability – 10 year** | | **Survival probability – 1 year** | | **Survival probability – 5 year** | | **Survival probability – 10 year** | |
|  | **M** | **F** | **M** | **F** | **M** | **F** | **M** | **F** | **M** | **F** | **M** | **F** |
| 1 | 1.000 | 0.988 | 1.000 | 0.988 | 1.000 | 0.988 | 1.000 | 1.000 | 1.000 | 1.000 | 1.000 | 1.000 |
| 2 | 1.000 | 0.994 | 1.000 | 0.994 | 1.000 | 0.994 | 1.000 | 1.000 | 1.000 | 1.000 | 1.000 | 1.000 |
| 3 | 1.000 | 0.996 | 1.000 | 0.996 | 1.000 | 0.996 | 1.000 | 1.000 | 1.000 | 1.000 | 1.000 | 1.000 |
| 4 | 1.000 | 0.997 | 1.000 | 0.997 | 1.000 | 0.997 | 1.000 | 1.000 | 1.000 | 1.000 | 1.000 | 1.000 |
| 5 | 0.998 | 0.995 | 0.998 | 0.995 | 0.998 | 0.995 | 1.000 | 1.000 | 1.000 | 1.000 | 1.000 | 1.000 |
| 6 | 0.998 | 0.996 | 0.998 | 0.996 | 0.998 | 0.996 | 1.000 | 1.000 | 1.000 | 1.000 | 1.000 | 1.000 |
| 7 | 0.998 | 0.996 | 0.998 | 0.996 | 0.998 | 0.996 | 1.000 | 1.000 | 1.000 | 1.000 | 1.000 | 1.000 |
| 8 | 0.998 | 0.997 | 0.998 | 0.997 | 0.998 | 0.997 | 1.000 | 1.000 | 1.000 | 1.000 | 1.000 | 1.000 |
| 9 | 0.999 | 0.997 | 0.999 | 0.997 | 0.999 | 0.997 | 1.000 | 1.000 | 1.000 | 1.000 | 1.000 | 1.000 |
| 10 | 0.999 | 0.997 | 0.999 | 0.997 | 0.999 | 0.997 | 1.000 | 1.000 | 1.000 | 1.000 | 1.000 | 1.000 |
| 11 | 0.999 | 0.998 | 0.999 | 0.998 | 0.999 | 0.998 | 1.000 | 1.000 | 1.000 | 1.000 | 1.000 | 1.000 |
| 12 | 0.999 | 0.998 | 0.999 | 0.998 | 0.999 | 0.998 | 1.000 | 1.000 | 1.000 | 1.000 | 1.000 | 1.000 |
| 13 | 0.999 | 0.998 | 0.999 | 0.998 | 0.999 | 0.998 | 1.000 | 1.000 | 1.000 | 1.000 | 1.000 | 1.000 |
| 14 | 0.999 | 0.998 | 0.999 | 0.998 | 0.999 | 0.998 | 1.000 | 1.000 | 1.000 | 1.000 | 1.000 | 1.000 |
| 15 | 0.995 | 0.997 | 0.995 | 0.997 | 0.995 | 0.997 | 1.000 | 1.000 | 1.000 | 1.000 | 1.000 | 1.000 |
| 16 | 0.995 | 0.997 | 0.995 | 0.997 | 0.995 | 0.997 | 1.000 | 1.000 | 1.000 | 1.000 | 1.000 | 1.000 |
| 17 | 0.996 | 0.997 | 0.996 | 0.997 | 0.996 | 0.997 | 1.000 | 1.000 | 1.000 | 1.000 | 1.000 | 1.000 |
| 18 | 0.996 | 0.997 | 0.996 | 0.997 | 0.996 | 0.997 | 1.000 | 1.000 | 1.000 | 1.000 | 1.000 | 1.000 |
| 19 | 0.996 | 0.997 | 0.996 | 0.997 | 0.996 | 0.997 | 1.000 | 1.000 | 1.000 | 1.000 | 1.000 | 1.000 |
| 20 | 0.996 | 0.997 | 0.996 | 0.997 | 0.996 | 0.997 | 1.000 | 1.000 | 1.000 | 1.000 | 1.000 | 1.000 |
| 21 | 0.997 | 0.998 | 0.997 | 0.998 | 0.997 | 0.998 | 1.000 | 1.000 | 1.000 | 1.000 | 1.000 | 1.000 |
| 22 | 0.997 | 0.998 | 0.997 | 0.998 | 0.997 | 0.998 | 1.000 | 1.000 | 1.000 | 1.000 | 1.000 | 1.000 |
| 23 | 0.997 | 0.998 | 0.997 | 0.998 | 0.997 | 0.998 | 1.000 | 1.000 | 1.000 | 1.000 | 1.000 | 1.000 |
| 24 | 0.997 | 0.998 | 0.997 | 0.998 | 0.997 | 0.998 | 1.000 | 1.000 | 1.000 | 1.000 | 1.000 | 1.000 |
| 25 | 0.948 | 0.994 | 0.948 | 0.994 | 0.948 | 0.994 | 1.000 | 1.000 | 1.000 | 1.000 | 1.000 | 1.000 |
| 26 | 0.950 | 0.994 | 0.950 | 0.994 | 0.950 | 0.994 | 1.000 | 1.000 | 1.000 | 1.000 | 1.000 | 1.000 |
| 27 | 0.952 | 0.994 | 0.952 | 0.994 | 0.952 | 0.994 | 1.000 | 1.000 | 1.000 | 1.000 | 1.000 | 1.000 |
| 28 | 0.953 | 0.994 | 0.953 | 0.994 | 0.953 | 0.994 | 1.000 | 1.000 | 1.000 | 1.000 | 1.000 | 1.000 |
| 29 | 0.955 | 0.994 | 0.955 | 0.994 | 0.955 | 0.994 | 1.000 | 1.000 | 1.000 | 1.000 | 1.000 | 1.000 |
| 30 | 0.956 | 0.995 | 0.956 | 0.995 | 0.956 | 0.995 | 1.000 | 1.000 | 1.000 | 1.000 | 1.000 | 1.000 |
| 31 | 0.958 | 0.995 | 0.958 | 0.995 | 0.958 | 0.995 | 1.000 | 1.000 | 1.000 | 1.000 | 1.000 | 1.000 |
| 32 | 0.959 | 0.995 | 0.959 | 0.995 | 0.959 | 0.995 | 1.000 | 1.000 | 1.000 | 1.000 | 1.000 | 1.000 |
| 33 | 0.960 | 0.995 | 0.960 | 0.995 | 0.960 | 0.995 | 1.000 | 1.000 | 1.000 | 1.000 | 1.000 | 1.000 |
| 34 | 0.962 | 0.995 | 0.962 | 0.995 | 0.962 | 0.995 | 1.000 | 1.000 | 1.000 | 1.000 | 1.000 | 1.000 |
| 35 | 0.987 | 0.986 | 0.987 | 0.986 | 0.987 | 0.986 | 1.000 | 1.000 | 1.000 | 1.000 | 1.000 | 1.000 |
| 36 | 0.987 | 0.987 | 0.987 | 0.987 | 0.987 | 0.987 | 1.000 | 1.000 | 1.000 | 1.000 | 1.000 | 1.000 |
| 37 | 0.987 | 0.987 | 0.987 | 0.987 | 0.987 | 0.987 | 1.000 | 1.000 | 1.000 | 1.000 | 1.000 | 1.000 |
| 38 | 0.988 | 0.987 | 0.988 | 0.987 | 0.988 | 0.987 | 1.000 | 1.000 | 1.000 | 1.000 | 1.000 | 1.000 |
| 39 | 0.988 | 0.988 | 0.988 | 0.988 | 0.988 | 0.988 | 1.000 | 1.000 | 1.000 | 1.000 | 1.000 | 1.000 |
| 40 | 0.988 | 0.988 | 0.988 | 0.988 | 0.988 | 0.988 | 1.000 | 1.000 | 1.000 | 1.000 | 1.000 | 1.000 |
| 41 | 0.989 | 0.988 | 0.989 | 0.988 | 0.989 | 0.988 | 1.000 | 1.000 | 1.000 | 1.000 | 1.000 | 1.000 |
| 42 | 0.989 | 0.989 | 0.989 | 0.989 | 0.989 | 0.989 | 1.000 | 1.000 | 1.000 | 1.000 | 1.000 | 1.000 |
| 43 | 0.989 | 0.989 | 0.989 | 0.989 | 0.989 | 0.989 | 1.000 | 1.000 | 1.000 | 1.000 | 1.000 | 1.000 |
| 44 | 0.989 | 0.989 | 0.989 | 0.989 | 0.989 | 0.989 | 1.000 | 1.000 | 1.000 | 1.000 | 1.000 | 1.000 |
| 45 | 0.977 | 0.976 | 0.977 | 0.976 | 0.977 | 0.976 | 1.000 | 1.000 | 1.000 | 1.000 | 1.000 | 1.000 |
| 46 | 0.982 | 0.980 | 0.982 | 0.980 | 0.982 | 0.980 | 1.000 | 1.000 | 1.000 | 1.000 | 1.000 | 1.000 |
| 47 | 0.985 | 0.983 | 0.985 | 0.983 | 0.985 | 0.983 | 1.000 | 1.000 | 1.000 | 1.000 | 1.000 | 1.000 |
| 48 | 0.987 | 0.985 | 0.987 | 0.985 | 0.987 | 0.985 | 1.000 | 1.000 | 1.000 | 1.000 | 1.000 | 1.000 |
| 49 | 0.989 | 0.987 | 0.989 | 0.987 | 0.989 | 0.987 | 1.000 | 1.000 | 1.000 | 1.000 | 1.000 | 1.000 |
| 50 | 0.990 | 0.988 | 0.990 | 0.988 | 0.990 | 0.988 | 1.000 | 1.000 | 1.000 | 1.000 | 1.000 | 1.000 |
| 51 | 0.991 | 0.989 | 0.991 | 0.989 | 0.991 | 0.989 | 1.000 | 1.000 | 1.000 | 1.000 | 1.000 | 1.000 |
| 52 | 0.992 | 0.990 | 0.992 | 0.990 | 0.992 | 0.990 | 1.000 | 1.000 | 1.000 | 1.000 | 1.000 | 1.000 |
| 53 | 0.993 | 0.991 | 0.993 | 0.991 | 0.993 | 0.991 | 1.000 | 1.000 | 1.000 | 1.000 | 1.000 | 1.000 |
| 54 | 0.993 | 0.992 | 0.993 | 0.992 | 0.993 | 0.992 | 1.000 | 1.000 | 1.000 | 1.000 | 1.000 | 1.000 |
| 55 | 0.983 | 0.981 | 0.983 | 0.981 | 0.983 | 0.981 | 1.000 | 1.000 | 1.000 | 1.000 | 1.000 | 1.000 |
| 56 | 0.984 | 0.982 | 0.984 | 0.982 | 0.984 | 0.982 | 1.000 | 1.000 | 1.000 | 1.000 | 1.000 | 1.000 |
| 57 | 0.985 | 0.984 | 0.985 | 0.984 | 0.985 | 0.984 | 1.000 | 1.000 | 1.000 | 1.000 | 1.000 | 1.000 |
| 58 | 0.986 | 0.984 | 0.986 | 0.984 | 0.986 | 0.984 | 1.000 | 1.000 | 1.000 | 1.000 | 1.000 | 1.000 |
| 59 | 0.987 | 0.985 | 0.987 | 0.985 | 0.987 | 0.985 | 1.000 | 1.000 | 1.000 | 1.000 | 1.000 | 1.000 |
| 60 | 0.988 | 0.986 | 0.988 | 0.986 | 0.988 | 0.986 | 0.845 | 0.858 | 0.845 | 0.858 | 0.845 | 0.858 |
| 61 | 0.988 | 0.987 | 0.988 | 0.987 | 0.988 | 0.987 | 0.866 | 0.876 | 0.866 | 0.876 | 0.866 | 0.876 |
| 62 | 0.989 | 0.987 | 0.989 | 0.987 | 0.989 | 0.987 | 0.882 | 0.890 | 0.882 | 0.890 | 0.882 | 0.890 |
| 63 | 0.989 | 0.988 | 0.989 | 0.988 | 0.989 | 0.988 | 0.894 | 0.901 | 0.894 | 0.901 | 0.894 | 0.901 |
| 64 | 0.990 | 0.988 | 0.990 | 0.988 | 0.990 | 0.988 | 0.904 | 0.910 | 0.904 | 0.910 | 0.904 | 0.910 |
| 65 | 0.977 | 0.970 | 0.977 | 0.970 | 0.977 | 0.970 | 0.431 | 0.438 | 0.431 | 0.438 | 0.431 | 0.438 |
| 66 | 0.980 | 0.974 | 0.980 | 0.974 | 0.980 | 0.974 | 0.637 | 0.640 | 0.637 | 0.640 | 0.637 | 0.640 |
| 67 | 0.982 | 0.977 | 0.982 | 0.977 | 0.982 | 0.977 | 0.734 | 0.735 | 0.734 | 0.735 | 0.734 | 0.735 |
| 68 | 0.983 | 0.979 | 0.983 | 0.979 | 0.983 | 0.979 | 0.790 | 0.791 | 0.790 | 0.791 | 0.790 | 0.791 |
| 69 | 0.985 | 0.981 | 0.985 | 0.981 | 0.985 | 0.981 | 0.826 | 0.827 | 0.826 | 0.827 | 0.826 | 0.827 |
| 70 | 0.986 | 0.983 | 0.986 | 0.983 | 0.986 | 0.983 | 0.852 | 0.852 | 0.852 | 0.852 | 0.852 | 0.852 |
| 71 | 0.987 | 0.984 | 0.987 | 0.984 | 0.987 | 0.984 | 0.871 | 0.871 | 0.871 | 0.871 | 0.871 | 0.871 |
| 72 | 0.988 | 0.985 | 0.988 | 0.985 | 0.988 | 0.985 | 0.886 | 0.886 | 0.886 | 0.886 | 0.886 | 0.886 |
| 73 | 0.989 | 0.986 | 0.989 | 0.986 | 0.989 | 0.986 | 0.897 | 0.898 | 0.897 | 0.898 | 0.897 | 0.898 |
| 74 | 0.989 | 0.987 | 0.989 | 0.987 | 0.989 | 0.987 | 0.907 | 0.907 | 0.907 | 0.907 | 0.907 | 0.907 |
| 75 | 0.960 | 0.949 | 0.960 | 0.949 | 0.960 | 0.949 | 0.482 | 0.445 | 0.482 | 0.445 | 0.482 | 0.445 |
| 76 | 0.964 | 0.956 | 0.964 | 0.956 | 0.964 | 0.956 | 0.658 | 0.642 | 0.658 | 0.642 | 0.658 | 0.642 |
| 77 | 0.967 | 0.962 | 0.967 | 0.962 | 0.967 | 0.962 | 0.744 | 0.736 | 0.744 | 0.736 | 0.744 | 0.736 |
| 78 | 0.969 | 0.966 | 0.969 | 0.966 | 0.969 | 0.966 | 0.796 | 0.790 | 0.796 | 0.790 | 0.796 | 0.790 |
| 79 | 0.972 | 0.969 | 0.972 | 0.969 | 0.972 | 0.969 | 0.830 | 0.826 | 0.830 | 0.826 | 0.830 | 0.826 |
| 80 | 0.973 | 0.972 | 0.973 | 0.972 | 0.973 | 0.972 | 0.854 | 0.852 | 0.854 | 0.852 | 0.854 | 0.852 |
| 81 | 0.975 | 0.974 | 0.975 | 0.974 | 0.975 | 0.974 | 0.872 | 0.870 | 0.872 | 0.870 | 0.872 | 0.870 |
| 82 | 0.977 | 0.976 | 0.977 | 0.976 | 0.977 | 0.976 | 0.887 | 0.885 | 0.887 | 0.885 | 0.887 | 0.885 |
| 83 | 0.978 | 0.978 | 0.978 | 0.978 | 0.978 | 0.978 | 0.898 | 0.896 | 0.898 | 0.896 | 0.898 | 0.896 |
| 84 | 0.979 | 0.979 | 0.979 | 0.979 | 0.979 | 0.979 | 0.907 | 0.906 | 0.907 | 0.906 | 0.907 | 0.906 |
| 85 | 0.935 | 0.922 | 0.935 | 0.922 | 0.935 | 0.922 | 0.645 | 0.581 | 0.645 | 0.581 | 0.645 | 0.581 |
| 86 | 0.938 | 0.926 | 0.938 | 0.926 | 0.938 | 0.926 | 0.734 | 0.699 | 0.734 | 0.699 | 0.734 | 0.699 |
| 87 | 0.941 | 0.930 | 0.941 | 0.930 | 0.941 | 0.930 | 0.787 | 0.764 | 0.787 | 0.764 | 0.787 | 0.764 |
| 88 | 0.944 | 0.933 | 0.944 | 0.933 | 0.944 | 0.933 | 0.821 | 0.805 | 0.821 | 0.805 | 0.821 | 0.805 |
| 89 | 0.946 | 0.936 | 0.946 | 0.936 | 0.946 | 0.936 | 0.846 | 0.833 | 0.846 | 0.833 | 0.846 | 0.833 |
| 90 | 0.948 | 0.939 | 0.948 | 0.939 | 0.948 | 0.939 | 0.864 | 0.853 | 0.864 | 0.853 | 0.864 | 0.853 |
| 91 | 0.950 | 0.942 | 0.950 | 0.942 | 0.950 | 0.942 | 0.878 | 0.869 | 0.878 | 0.869 | 0.878 | 0.869 |
| 92 | 0.951 | 0.944 | 0.951 | 0.944 | 0.951 | 0.944 | 0.889 | 0.881 | 0.889 | 0.881 | 0.889 | 0.881 |
| 93 | 0.953 | 0.946 | 0.953 | 0.946 | 0.953 | 0.946 | 0.899 | 0.891 | 0.899 | 0.891 | 0.899 | 0.891 |
| 94 | 0.955 | 0.948 | 0.955 | 0.948 | 0.955 | 0.948 | 0.906 | 0.900 | 0.906 | 0.900 | 0.906 | 0.900 |
| 95 | 0.956 | 0.950 | 0.956 | 0.950 | 0.956 | 0.950 | 0.913 | 0.907 | 0.913 | 0.907 | 0.913 | 0.907 |
| 96 | 0.957 | 0.951 | 0.957 | 0.951 | 0.957 | 0.951 | 0.918 | 0.912 | 0.918 | 0.912 | 0.918 | 0.912 |
| 97 | 0.958 | 0.953 | 0.958 | 0.953 | 0.958 | 0.953 | 0.923 | 0.917 | 0.923 | 0.917 | 0.923 | 0.917 |
| 98 | 0.960 | 0.954 | 0.960 | 0.954 | 0.960 | 0.954 | 0.927 | 0.922 | 0.927 | 0.922 | 0.927 | 0.922 |
| 99 | 0.961 | 0.956 | 0.961 | 0.956 | 0.961 | 0.956 | 0.931 | 0.926 | 0.931 | 0.926 | 0.931 | 0.926 |
| 100 | 0.962 | 0.957 | 0.962 | 0.957 | 0.962 | 0.957 | 0.934 | 0.929 | 0.934 | 0.929 | 0.934 | 0.929 |
| 101 | 0.963 | 0.958 | 0.963 | 0.958 | 0.963 | 0.958 | 0.937 | 0.932 | 0.937 | 0.932 | 0.937 | 0.932 |
| 102 | 0.964 | 0.959 | 0.964 | 0.959 | 0.964 | 0.959 | 0.939 | 0.935 | 0.939 | 0.935 | 0.939 | 0.935 |
| 103 | 0.964 | 0.960 | 0.964 | 0.960 | 0.964 | 0.960 | 0.942 | 0.937 | 0.942 | 0.937 | 0.942 | 0.937 |
| 104 | 0.965 | 0.961 | 0.965 | 0.961 | 0.965 | 0.961 | 0.944 | 0.939 | 0.944 | 0.939 | 0.944 | 0.939 |
| 105 | 0.966 | 0.962 | 0.966 | 0.962 | 0.966 | 0.962 | 0.946 | 0.941 | 0.946 | 0.941 | 0.946 | 0.941 |
| 106 | 0.967 | 0.963 | 0.967 | 0.963 | 0.967 | 0.963 | 0.948 | 0.943 | 0.948 | 0.943 | 0.948 | 0.943 |
| 107 | 0.967 | 0.964 | 0.967 | 0.964 | 0.967 | 0.964 | 0.949 | 0.945 | 0.949 | 0.945 | 0.949 | 0.945 |
| 108 | 0.968 | 0.965 | 0.968 | 0.965 | 0.968 | 0.965 | 0.951 | 0.946 | 0.951 | 0.946 | 0.951 | 0.946 |
| 109 | 0.969 | 0.965 | 0.969 | 0.965 | 0.969 | 0.965 | 0.952 | 0.948 | 0.952 | 0.948 | 0.952 | 0.948 |
| 109+ | 0.969 | 0.965 | 0.969 | 0.965 | 0.969 | 0.965 | 0.952 | 0.948 | 0.952 | 0.948 | 0.952 | 0.948 |

### Lung Cancer

**Table P. Probability of 1, 5 and 1 year survival computed from prevalence and mortality data for Lung Cancer**

| Age | Survival probability – 1 year | | Survival probability – 5 year | | Survival probability – 10 year | |
| --- | --- | --- | --- | --- | --- | --- |
|  | **M** | **F** | **M** | **F** | **M** | **F** |
| 15-39 | 0.615 | 0.663 | 0.615 | 0.663 | 0.615 | 0.663 |
| 40-49 | 0.751 | 0.774 | 0.751 | 0.774 | 0.751 | 0.774 |
| 50-59 | 0.784 | 0.802 | 0.784 | 0.802 | 0.784 | 0.802 |
| 60-69 | 0.790 | 0.818 | 0.790 | 0.818 | 0.790 | 0.818 |
| 70-79 | 0.825 | 0.859 | 0.825 | 0.859 | 0.825 | 0.859 |
| >79 | 0.945 | 0.988 | 0.945 | 0.988 | 0.945 | 0.988 |

# Exposure data

This section provides additional methods data on the exposure data to supplement those found in the methods section of the manuscript.

We obtained data from the AURN on rural background concentrations of NO_2_ for 2009 and PM_2.5_ for 2010 and for both pollutants for 2015. In England, there are three and seven concomitant sites for PM_2.5_ and NO_2_, respectively, for all years, and with sufficient data for extrapolation purposes (i.e. at least 75% of days operating within each year). We used one site in Scotland (Auchencorth Moss) to represent PM_2.5_ rural background concentrations in Northern England as there were no other geographically appropriate rural sites available. Table R shows average concentrations of PM_2.5_ and NO_2_ at rural background stations in each year and the difference in average concentrations between each pair of years. This illustrates the method used for forward extrapolation.

**Table Q. Average rural and suburban background concentrations (µg/m3) of PM2.5 in 2010 and NO2 in 2009 and for both pollutants in 2015 as well as absolute differences in concentrations between 2009/2010 and 2015.**

| Pollutant | Sites | Measured rural background concentrations | | Extrapolation (differencing) |
| --- | --- | --- | --- | --- |
|  |  | **2009/2010** | **2015** | **2009/2010 to 2015** |
| PM_2.5_ | 3 | 8.0 | 6.8 | -1.2 |
| NO_2_ | 7 | 10.7 | 8.4 | -2.3 |

Information on background exposure were derived from 2015 satellite-derived PM_2.5_ estimates. These estimates were obtained from the Atmospheric Composition Analysis Group at Dalhousie University, Canada (spatial scale ~ 620m x 620m). We subtracted dust and sea-salt free PM_2.5_ estimates from total PM_2.5_ estimates to obtain background PM_2.5_ estimates. We did not include information on background NO_2_ estimates as this data was not readily available for this study and most NO_2_ is from anthropogenic sources with a very low, spatially non-varying background component.

# References

1. Office for National Statistics. Mid-2015 Population estimates: Pivot table analysis tool for the UK. 2016. Available from: <https://www.ons.gov.uk/peoplepopulationandcommunity/populationandmigration/populationestimates/datasets/populationestimatesforukenglandandwalesscotlandandnorthernireland>.

2. Office for National Statistics. Birth Summary Tables - England and Wales 2016 [cited June 2018]. Available from: <https://www.ons.gov.uk/releases/birthsummarytablesinenglandandwales2016>.

3. Office for National Statistics. Deaths registered in England and Wales: 2015 2016 [cited June 2018]. Available from: <https://www.ons.gov.uk/peoplepopulationandcommunity/birthsdeathsandmarriages/deaths/bulletins/deathsregistrationsummarytables/2015>.

4. Jacquemin B, Siroux V, Sanchez M, Carsin AE, Schikowski T, Adam M, et al. Ambient air pollution and adult asthma incidence in six European cohorts (ESCAPE). Environmental health perspectives. 2015;123(6):613-21. Epub 2015/02/26. doi: 10.1289/ehp.1408206. PubMed PMID: 25712593; PubMed Central PMCID: PMCPMC4455584.

5. Curti S, Gori D, Di Gregori V, Farioli A, Baldasseroni A, Fantini MP, et al. PubMed search filters for the study of putative outdoor air pollution determinants of disease. BMJ open. 2016;6(12):e013092. Epub 2016/12/23. doi: 10.1136/bmjopen-2016-013092. PubMed PMID: 28003291.

6. Davies HT, Crombie IK, Tavakoli M. When can odds ratios mislead? BMJ. 1998;316(7136):989-91. PubMed PMID: 9550961; PubMed Central PMCID: PMCPMC1112884.

7. Department for Environment FaRA. Conversion Factors Between ppb and µg m-3 and ppm and mgm-3 2014. Available from: <https://uk-air.defra.gov.uk/assets/documents/reports/cat06/0502160851_Conversion_Factors_Between_ppb_and.pdf>.

8. Committee on the Medical Effects of Air Pollutants. COMEAP: review of the UK air quality index. 2011.

9. Anderson HR, Favarato G, Atkinson RW. Long-term exposure to air pollution and the incidence of asthma: meta-analysis of cohort studies. Air Quality, Atmosphere & Health. 2013;6(1):47-56.

10. Defra. UK Plan for tackling roadside nitrogen dioxide concentrations: Technical report. 2017.

11. British Lung Foundation. Asthma Statistics 2015. Available from: <https://statistics.blf.org.uk/asthma>.

12. Khreis H, Kelly C, Tate J, Parslow R, Lucas K, Nieuwenhuijsen M. Exposure to traffic-related air pollution and risk of development of childhood asthma: A systematic review and meta-analysis. Environment international. 2016. Epub 2016/11/25. doi: 10.1016/j.envint.2016.11.012. PubMed PMID: 27881237.

13. Public Health England. COPD Prevalence Estimates London: Public Health England; 2008. Available from: <http://www.erpho.org.uk/viewResource.aspx?id=20574>.

14. Committee On The Medical Effects Of Air Pollutants. Long-term Exposure term to Air Pollution and Chronic Bronchitis. 2016. Available from: <https://www.gov.uk/government/publications/comeap-long-term-exposure-to-air-pollution-and-chronic-bronchitis>.

15. Smolina K, Wright FL, Rayner M, Goldacre MJ. Determinants of the decline in mortality from acute myocardial infarction in England between 2002 and 2010: linked national database study. Corrected data on incidence and mortality in 2013 at <http://www.bmj.com/content/347/bmj.f7379.abstract>. BMJ. 2012;344:d8059. doi: 10.1136/bmj.d8059. PubMed PMID: 22279113; PubMed Central PMCID: PMCPMC3266430.

16. British Heart Foundation. Cardiovascular Disease Statistics 2014 2015. Available from: <https://www.bhf.org.uk/research/heart-statistics/heart-statistics-publications/cardiovascular-disease-statistics-2014>.

17. Cesaroni G, Forastiere F, Stafoggia M, Andersen ZJ, Badaloni C, Beelen R, et al. Long term exposure to ambient air pollution and incidence of acute coronary events: prospective cohort study and meta-analysis in 11 European cohorts from the ESCAPE Project. Bmj. 2014;348:f7412.

18. NHS Digital. National Diabetes Audit 2015/2016 NHS Digital2017. Available from: <http://www.content.digital.nhs.uk/catalogue/PUB23241>.

19. Eze IC, Hemkens LG, Bucher HC, Hoffmann B, Schindler C, Kunzli N, et al. Association between ambient air pollution and diabetes mellitus in Europe and North America: systematic review and meta-analysis. Environmental health perspectives. 2015;123(5):381-9. Epub 2015/01/28. doi: 10.1289/ehp.1307823. PubMed PMID: 25625876; PubMed Central PMCID: PMCPMC4421762.

20. British Heart Foundation. Stroke Statistics 2009 2009. Available from: <https://www.bhf.org.uk/-/media/files/publications/research/hs2009_stroke_statistics.pdf>.

21. Scheers H, Jacobs L, Casas L, Nemery B, Nawrot TS. Long-Term Exposure to Particulate Matter Air Pollution Is a Risk Factor for Stroke: Meta-Analytical Evidence. Stroke. 2015;46(11):3058-66. Epub 2015/10/16. doi: 10.1161/strokeaha.115.009913. PubMed PMID: 26463695.

22. Alzheimer's Society. Dementia UK - second edition: Alzheimer's UK; 2014. Available from: <https://www.alzheimers.org.uk/info/20025/policy_and_influencing/251/dementia_uk>.

23. Oudin A, Forsberg B, Adolfsson AN, Lind N, Modig L, Nordin M, et al. Traffic-Related Air Pollution and Dementia Incidence in Northern Sweden: A Longitudinal Study. Environmental health perspectives. 2016;124(3):306-12. Epub 2015/08/26. doi: 10.1289/ehp.1408322. PubMed PMID: 26305859; PubMed Central PMCID: PMCPMC4786976.

24. Office for National Statistics. Birth characteristics 2015 2016. Available from: <https://www.ons.gov.uk/peoplepopulationandcommunity/birthsdeathsandmarriages/livebirths/datasets/birthcharacteristicsinenglandandwales>.

25. Pedersen M, Giorgis-Allemand L, Bernard C, Aguilera I, Andersen AM, Ballester F, et al. Ambient air pollution and low birthweight: a European cohort study (ESCAPE). The Lancet Respiratory medicine. 2013;1(9):695-704. Epub 2014/01/17. doi: 10.1016/s2213-2600(13)70192-9. PubMed PMID: 24429273.

26. Cancer Research UK. Statistics by cancer type - Average Number of New Cases Per Year and Age-Specific Incidence Rates per 100,000 Population, UK 2012-2014 2017. Available from: <http://www.cancerresearchuk.org/health-professional/cancer-statistics/statistics-by-cancer-type>.

27. Statistics OfN. Cancer Survival in England - Adults Diagnosed: 2010 to 2014, followed up to 2015. 2016.

28. Office for National Statistics. Cancer Survival in England: adults diagnosed 2008–2012 and followed up to 2013. 2014.

29. Hamra GB, Laden F, Cohen AJ, Raaschou-Nielsen O, Brauer M, Loomis D. Lung Cancer and Exposure to Nitrogen Dioxide and Traffic: A Systematic Review and Meta-Analysis. Environmental health perspectives. 2015;123(11):1107-12. Epub 2015/04/15. doi: 10.1289/ehp.1408882. PubMed PMID: 25870974; PubMed Central PMCID: PMCPMC4629738.

30. Hamra GB, Guha N, Cohen A, Laden F, Raaschou-Nielsen O, Samet JM, et al. Outdoor particulate matter exposure and lung cancer: a systematic review and meta-analysis. Environmental health perspectives. 2014;122(9):906-11. Epub 2014/06/10. doi: 10.1289/ehp.1408092. PubMed PMID: 24911630; PubMed Central PMCID: PMCPMC4154221.

31. World Health Organization. Review of evidence on health aspects of air pollution–REVIHAAP Project. World Health Organization, Copenhagen, Denmark. 2013.

32. Royal College of Physicians. Every breath we take: The lifelong impact of air pollution 2016. Available from: <https://www.rcplondon.ac.uk/projects/outputs/every-breath-we-take-lifelong-impact-air-pollution>.

33. US Environmental Protection Agency. Integrated Science Assessment for Particulate Matter (Final Report, Dec 2009) 2009. Available from: <https://cfpub.epa.gov/ncea/isa/recordisplay.cfm?deid=216546>.

34. International Agency for Research on Cancer. IARC monograph on the evaluation of carcinogenic risks to humans 2016 [cited Outdoor air pollution ]. Available from: <http://monographs.iarc.fr/ENG/Monographs/vol109/mono109.pdf>.

35. Committee On The Medical Effects Of Air Pollutants (COMEAP). COMEAP: long-term exposure to air pollution and chronic bronchitis 2016. Available from: <https://www.gov.uk/government/publications/comeap-long-term-exposure-to-air-pollution-and-chronic-bronchitis>.

36. Committee on the medical effects of air pollutant (COMEAP). Statement on the evidence for the effects of nitrogen dioxide on health 2015. Available from: <https://www.gov.uk/government/publications/nitrogen-dioxide-health-effects-of-exposure>.

37. US Environmental Protection Agency. Integrated science assessment for oxides of nitrogen – health criteria (final report 2016 EPA/600/R-15/068). 2016.

38. Dr. Craig Currie at Cardiff University. Incidence data: Type 2 diabetes Personal communication.
