# Supplementary material for: Estimating the costs of air pollution to the National Health Service and social care: An assessment and forecast up to 2035
Source: PLoS Med. 2018 Jul 10;15(7):e1002602. doi: 10.1371/journal.pmed.1002602 (PMC6039053; doi:10.1371/journal.pmed.1002602)
Supplement: S3 Text — (DOCX) [file pmed.1002602.s003.docx]

## S4 TEXT: COST METHODS

Relevant sources were collated using a systematic literature review with Mesh terms in PubMed, and completed using Google searches. While multiple studies from the search results were considered, the most relevant, recent studies were used for the final cost estimates The search terms are summarised in. **Table A**. The majority of search results were disregarded because they were theoretical, the cost breakdown was not granular enough or the figures were based on literature that had previously already been stated within the literature review. Papers which have been considered for the final cost estimate are listed in the consecutive tables, while it is also indicated which estimate was used for the final model. If the search with Mesh terms on PubMed returned no results, Google was consulted as a supporting search engine.

**Table B** and **Table C** provide an overview of the overall costs used in the microsimulation model per type of care. These costs were adjusted for inflation in 2015-16 using the Hospital and Community Health Services inflation index,^[[1]](#footnote-1)^ and divided by the prevalence in order to have a “cost per case”.

**Table A. MeSh search terms**

| Condition | Search term | Results found by PubMed | Ambulance | Social Care | Outpatient | Outpatient drugs | A&E |
| --- | --- | --- | --- | --- | --- | --- | --- |
| COPD | (("cost of disease"[MeSH Terms] OR "cost of illness"[MeSH Terms]) AND "pulmonary disease, chronic obstructive"[MeSH Terms]) AND "united kingdom"[MeSH Terms] | 18 | 1 | 4 |  |  |  |
| Stroke | ((((cost of disease[MeSH Terms]) OR cost of illness[MeSH Terms]) AND acute stroke[MeSH Terms])) AND united kingdom[MeSH Terms] | 22 | 1 | 4 | 2 | 1 | 0 |
| Diabetes | ((((cost of disease[MeSH Terms]) OR cost of illness[MeSH Terms]) AND united kingdom[MeSH Terms])) AND Diabetes Mellitus[MeSH Terms] | 38 |  | 3 | 2 | 1 |  |
| Asthma | ((((cost of disease[MeSH Terms]) OR cost of illness[MeSH Terms]) AND united kingdom[MeSH Terms])) AND Asthma[MeSH Terms] | 19 | 1 | 3 | 1 | 0 | 2 |
| CHD | ((((cost of disease[MeSH Terms]) OR cost of illness[MeSH Terms]) AND united kingdom[MeSH Terms])) AND coronary disease[MeSH Terms] | 14 |  | 3 | 2 | 1 | 1 |
| Lung Cancer | ((((cost of disease[MeSH Terms]) OR cost of illness[MeSH Terms]) AND united kingdom[MeSH Terms])) AND Lung Neoplasms | 12 |  | 1 | 1 |  |  |
| Dementia | ((((cost of disease[MeSH Terms]) OR cost of illness[MeSH Terms]) AND united kingdom[MeSH Terms])) AND dementia[MeSH Terms] | 65 | 1 | 3 | 1 | 1 | 0 |

Table B. **Cost-per-case based on total prevalence of each disease, expressed in £ per case**

| Cost type (£ per case) | Primary Care | Social Care | Medication | Secondary |
| --- | --- | --- | --- | --- |
| Asthma | 21.28 | 0.50 | 87.57 | 27.02 |
| Chronic Obstructive Pulmonary Disease (COPD) | 400.43 | 85.30 | 126.79 | 587.48 |
| Coronary Heart Disease (CHD) | 71.57 | 109.70 | 818.60 | 1460.46 |
| Stroke | 36.45 | 76.05 | 504.10 | 722.84 |
| Diabetes | 375.00 | 601.56 | 276.88 | 536.75 |
| Lung cancer | 51.73 | 89.38 | 35.10 | 466.63 |
| Dementia | 430.62 | 6174.47 | 310.24 | 197.24 |

**Table C Final references used to calculate cost per case**

|  |  | Source | Definition | Costs | Units | Year | FINAL COST 2015 (same unit) | Region | Final Costs 2015 per case (£) | Adjustments |
| --- | --- | --- | --- | --- | --- | --- | --- | --- | --- | --- |
| Asthma | Inpatient care | HES - Total | ICD-10: J45 | 90.85 | M | 2010-11 | 97.50 | England | **14.81** |  |
|  | Outpatient care | Neville et al. 2003 [1] | Extrapolated from their study in Scotland | 12.21 | per case | 2015 | 12.21 | Scotland | **12.21** | = Cost per case as the "prevalence" in the case of asthma is proxied by the number of patients |
|  | Primary care | Mukherjee et al. 2016 [2] | READ Codes (appendices 2 and 3), and Weekly Returns Service (WRS) of the Royal College of General Practitioners | 134.27 | M | 2011-12 | 141.13 | England | **21.28** |  |
|  | Prescription | Mukherjee et al. 2016 [2] | Community prescribing based on British National Forumulary (BNF) | 552.51 | M | 2011-12 | 580.75 | England | **87.57** |  |
|  | Social care | Georghiou et al. 2012 [3] | Final year of life (12m before death)  Main source: local authorities, it excludes self-funded cares. | 2567 | per death | 2010-11 | 2754.87 | England | **0.506** | (Cost per patient * nb. Annual death)/prevalence |
| CHD | Inpatient care | HES - Total | ICD-10: I20-I25 | 1278.17 | M | 2010-11 | 1371.71 | England | **1411.60** |  |
|  | Outpatient care | Liu 2002 [4] | TOP-DOWN. The number of CHD outpatient hospital attendances was estimated by using CHD prevalence data from the Health survey for England, and the difference in referral rates between CHD patients and all patients.  We assumed that half of the inpatient hospital episodes related to CHD were followed by at least one outpatient attendance. | 33.32 | M | 1999 | 52.47 | UK | **48.87** |  |
|  | Primary care | Liu 2002 [4] | Doctor and nurse consultations at home or at clinic | 48.8 | M | 1999 | 76.85019967 | UK | **71.57** |  |
|  | Prescription | Liu 2002 [4] | Prescriptions only (does not include dispensing) | 558.17 | M | 1999 | 879.0056547 | UK | **818.60** |  |
|  | Social care | Liu 2002 [4] | Community health and social services  Main sources: Local Authorities and the NHS | 74.8 | M | 1999 | 117.7949782 | UK | **109.70** |  |
| Dementia | Inpatient care | HES - Total | ICD-10: F00-F03 Does not include specialised hospitals | 80.26 | M | 2010-11 | 86.12911859 | England | **122.48** |  |
|  | Outpatient care | Luengo-Fernandez et al. 2010 [5] |  | 317.02 | M | 2008 | 352.5270517 | UK | **430.62** |  |
|  | Primary care | Luengo-Fernandez et al. 2010 [5] |  | 55.044 | M | 2008 | 61.20964817 | UK | **74.77** |  |
|  | Prescription | Luengo-Fernandez et al. 2010 [5] |  | 228.40 | M | 2008 | 253.9826763 | UK | **310.24** |  |
|  | Social care | Luengo-Fernandez et al. 2010 [5] | Main source: Long-term care (residential and nursing homes) and 65+ only | 9091.18 | M | 2008 | 10109.50777 | UK | **6174.47** |  |
| Diabetes type 2 | Inpatient care | HES - Total | ICD-10: E10, E11 and O24.4 | 162.03 | M | 2010-11 | 173.8833505 | England | **76.16** |  |
|  | Outpatient care | Kanavos et al. (2012) [6] | The UK numbers were obtained by taking 2 studies, one focusing on outpatient data (Currie et al. 2010) and the other on inpatient data (Morgan et al. 2010 - complications from diabetes) similar data times, and combining the two to create an annual per patient cost and applying to the latest APHO Prevalence Modelling data to arrive at total expenditure figures; extrapolation to 2010 was achieved by using the GDP deflator. Both diabetes and complications costs are accounted for. | 1158 | M | 2010 | 1242.74851 | UK | **460.60** |  |
|  | Primary care | Hex et al. 2012 [7] | Top-down, calculate excess primary care consultation rate | 950.71 | M | 2010-11 | 1020.292047 | UK | **375.00** |  |
|  | Prescription | Hex et al. 2012 [7] | Includes insulin pump | 701.94 | M | 2010-11 | 753.3067761 | UK | **276.88** |  |
|  | Social care | Institute of Diabetes for Older People and Novo Nordisk 2013 [8] | Includes complications related to diabetes, and refers to Hex et al. (2012) figures, and rely on the assumption that 90% of diabetic patient is type 2.  Main source: residential, nursing, or home care settings | 1279.8 | M | 2010-11 | 1373.462472 | England | **601.56** |  |
| COPD | Inpatient care | HES - Total | ICD-10: J40-J44 | 471.06 | M | 2010-11 | 505.5389808 | England | **582.60** |  |
|  | Outpatient care | Guest (1999) [9] |  | 4.5 | M | 1996/1997 | 4.650297415 | UK | **4.89** |  |
|  | Primary care | Guest (1999) [9] | GP Consultations + GP-initiated diagnostic tests | 369.2 | M | 1996/1997 | 381.5310679 | UK | **400.43** |  |
|  | Prescription | Guest (1999) | Selected drugs in the Medical Data Index database | 116.9 | M | 1996/1997 | 120.8043928 | UK | **126.79** |  |
|  | Social care | Georghiou et al. 2012 [3] | Only palliative care  Main source: local authorities, it excludes self-funded cares. | 2602 | per death | 2010-11 | 2792.427998 | England | **85.30** |  |
| Lung Cancer | Inpatient care | HES - Total | ICD-10: C34 | 208.42 | M | 2010-11 | 223.6686132 | England | **462.14** |  |
|  | Outpatient care | Guest et al. (2006) [10] | Palliative care only | 48 | per death | 2000-01 | 72.54353199 | England | **4.49** | (Cost per patient * nb. Annual death)/prevalence |
|  | Primary care | Guest et al. (2006) [10] | Palliative care only (GP surgery + district nurse visits) | 553.2 | per death | 2000-01 | 836.0642062 | England | **51.73** | (Cost per patient * nb. Annual death)/prevalence |
|  | Prescription | Guest et al. (2006) [10] | Palliative care only | 375.4 | per death | 2000-01 | 567.3508731 | England | **35.10** | (Cost per patient * nb. Annual death)/prevalence |
|  | Social care | Georghiou et al. 2012 [3] | All types of cancer and only over the last 12 months prior death  Main source: local authorities, it excludes self-funded cares. | 1346 | per death | 2010-11 | 1444.507335 | England | **89.38** | (Cost per patient * nb. Annual death)/prevalence |
| Stroke | Inpatient care | HES - Total | ICD-10: I60-I63 | 658.28 | M | 2010-11 | 706.461241 | England | **649.93** |  |
|  | Outpatient care | Saka et al. (2009) [11] | We have allocated 2/3 of outpatient cost | 73.12 | M | 2005 | 90.14710801 | UK | **72.90** |  |
|  | Primary care | Saka et al. (2009) [11] | We have allocated 1/3 of outpatient cost | 36.56 | M | 2005 | 45.073554 | UK | **36.45** |  |
|  | Prescription | Saka et al. (2009) [11] | Oupatient drugs | 505.59 | M | 2005 | 623.3275656 | UK | **504.10** |  |
|  | Social care | Georghiou et al. 2012 [11] | Ischaemic heart disease SC cost for palliative treatment only  Main source: local authorities, it excludes self-funded cares. | 2908 | per death | 2010-11 | 3120.822682 | England | **76.05** | (Cost per patient * nb. Annual death)/prevalence |

Lung cancer costs were the most difficult to estimate, and costs from the literature only provided the financial burden of lung cancer over the last months of life. Dementia costs were taken from the HES database, however this does not include specialist hospitals, which implies that a conservative approach has been used (i.e. Underestimation of the costs).

**References**

1. Neville R HG, Smith B, McCowan C. The economic and human costs of asthma in Scotland. . Primary care respiratory journal. 2003;12(4):115-9.

2. Mukherjee M, Stoddart A, Gupta RP, Nwaru BI, Farr A, Heaven M, et al. The epidemiology, healthcare and societal burden and costs of asthma in the UK and its member nations: analyses of standalone and linked national databases. BMC Medicine. 2016;14(1):113. doi: 10.1186/s12916-016-0657-8.

3. Georghiou T DS, Davies A, Bardsley M Understanding patterns of health and social care at the end of life. 2012.

4. Liu JL, Maniadakis N, Gray A, Rayner M. The economic burden of coronary heart disease in the UK. Heart. 2002;88(6):597-603. Epub 2002/11/16.

5. Luengo-Fernandez R LJ, Gray A. The economic burden of dementia and associated research funding in the United Kingdom. Cambridge: Health Economics Research Centre, University of Oxford for the Alzheimer’s Research Trust, 2010.

6. Kanavos P vdAS, Schurer W. Diabetes expenditure, burden of disease and management in 5 EU countries. London School of Economics, 2012.

7. Hex N, Bartlett C, Wright D, Taylor M, Varley D. Estimating the current and future costs of Type 1 and Type 2 diabetes in the UK, including direct health costs and indirect societal and productivity costs. Diabet Med. 2012;29(7):855-62. Epub 2012/04/28. doi: 10.1111/j.1464-5491.2012.03698.x.

8. Novo Nordisk Ltd. Behind closed doors: The hidden impact of diabetes in social care. 2013.

9. Guest JF. The Annual Cost of Chronic Obstructive Pulmonary Disease to the UK’s National Health Service. Disease Management and Health Outcomes. 1999;5(2):93-100. doi: 10.2165/00115677-199905020-00004.

10. Guest JF, Ruiz FJ, Greener MJ, Trotman IF. Palliative care treatment patterns and associated costs of healthcare resource use for specific advanced cancer patients in the UK. Eur J Cancer Care (Engl). 2006;15(1):65-73. Epub 2006/01/31. doi: 10.1111/j.1365-2354.2005.00623.x.

11. Saka O, McGuire A, Wolfe C. Cost of stroke in the United Kingdom. Age Ageing. 2009;38(1):27-32. Epub 2009/01/15. doi: 10.1093/ageing/afn281.

1. Department of Health (2015.6 Pay & Price series.xls) “Hospital and Community Health Services (HCHS) pay and price inflation is a weighted average of two separate inflation indices, the Pay Cost Index (PCI) and the Health Service Cost Index (HSCI)” [↑](#footnote-ref-1)
