# Supplementary material for: Estimating the costs of air pollution to the National Health Service and social care: An assessment and forecast up to 2035
Source: PLoS Med. 2018 Jul 10;15(7):e1002602. doi: 10.1371/journal.pmed.1002602 (PMC6039053; doi:10.1371/journal.pmed.1002602)
Supplement: S4 Text — (DOCX) [file pmed.1002602.s004.docx]

## S3 TEXT: RESULTS BY DISEASE AND COST PARAMETER

# PM_2.5_

By 2035 it was predicted that there would be 1,327,424 [±9919] new cases of disease attributable to PM_2.5_. CHD and diabetes cases were the greatest contributors to the total cases attributable, with 348,878[±2561] and 273,767[±7683] attributable to PM_2.5_ between 2017 and 2035, respectively. Table A presents the costs attributable to PM_2.5_ by disease for each cost parameter (primary care, secondary care, medication, social care).(**Table A**)

**Table A Cumulative incidence cases attributable to PM2.5 in England, by disease and total between 2017 and 2035.**

| **Year** | **Asthma** | **COPD** | **Diabetes** | **CHD** | **Lung cancer** | **Stroke** | **Low birthweight** | **Total** |
| --- | --- | --- | --- | --- | --- | --- | --- | --- |
| 2017 | 5,564  [±556] | 11,684  [±556] | 14,467  [±1669] | 16,136  [±556] | 1,669  [±556] | 4,451  [±556] | 9,459  [±556] | 63,430  [±2154] |
| 2017-2025 | 62,992  [±1717] | 112,235  [±1717] | 127,578  [±5152] | 154,053  [±1717] | 20,636  [±1717] | 47,499  [±1717] | 82,924  [±1717] | 607,917  [±6651] |
| 2017-2035 | 133,356  [±2575] | 246,916  [±2575] | 273,767  [±7725] | 348,878  [±2575] | 44,290  [±2575] | 106,331  [±2575] | 173,886  [±2575] | 1,327,424  [±9972] |

**Table B** presents the costs attributable to PM_2.5_ by disease for each cost parameter (primary care, secondary, medication, social care). **Table C** presents the costs avoided by disease and cost parameter (£million) due to each scenario: a 1 µg/m3 reduction PM_2.5_ in relative to baseline.

**Table B. Attributable costs by disease and cost parameter (PM2.5) (£million) ALL DISEASES**

|  |  | Asthma | COPD | Diabetes | CHD | Lung Cancer | Stroke | Total |  |
| --- | --- | --- | --- | --- | --- | --- | --- | --- | --- |
| Primary care | 2017 | 0.22[±0.16] | 4.61[±0.64] | 4.42[±2.49] | 0.97[±0.2] | 0.08[±0.04] | 0.15[±0.09] | 10.45[±2.59] |  |
|  | 2017-2025 | 6.47[±0.52] | 134.26[±2.09] | 183.13[±8.28] | 6.89[±0.67] | 0.22[±0.15] | 1.06[±0.3] | 332.02[±8.59] |  |
|  | 2017-2035 | 23.92[±0.86] | 379.11[±3.45] | 655.47[±13.76] | 10.23[±1.11] | 0.21[±0.26] | 1.44[±0.51] | 1070.37[±14.27] |  |
| Secondary care | 2017 | 0.28[±0.2] | 6.76[±0.93] | 6.32[±3.57] | 19.8[±4.14] | 0.74[±0.4] | 2.93[±1.8] | 36.83[±5.84] |  |
|  | 2017-2025 | 8.21[±0.66] | 196.98[±3.06] | 262.12[±11.86] | 767.73[±13.61] | 15.02[±1.39] | 118.64[±6.03] | 1368.7[±19.34] |  |
|  | 2017-2035 | 30.36[±1.09] | 556.2[±5.05] | 938.19[±19.7] | 2597.12[±22.63] | 35.54[±2.38] | 380.26[±10.16] | 4537.67[±32.18] |  |
| Medication | 2017 | 0.9[±0.66] | 1.46[±0.2] | 3.26[±1.84] | 11.1[±2.32] | 0.06[±0.03] | 2.04[±1.25] | 18.81[±3.29] |  |
|  | 2017-2025 | 26.61[±2.15] | 42.51[±0.66] | 135.21[±6.12] | 430.32[±7.63] | 1.13[±0.11] | 82.74[±4.2] | 718.53[±10.88] |  |
|  | 2017-2035 | 98.41[±3.52] | 120.04[±1.09] | 483.96[±10.16] | 1455.71[±12.68] | 2.67[±0.18] | 265.19[±7.08] | 2425.98[±18.11] |  |
| Social care | 2017 | 0.01[±0] | 0.98[±0.14] | 7.09[±4] | 1.49[±0.31] | 0.14[±0.08] | 0.31[±0.19] | 10.01[±4.02] |  |
|  | 2017-2025 | 0.15[±0.01] | 28.6[±0.44] | 293.77[±13.29] | 57.67[±1.02] | 2.88[±0.27] | 12.48[±0.63] | 395.54[±13.35] |  |
|  | 2017-2035 | 0.56[±0.02] | 80.76[±0.73] | 1051.47[±22.08] | 195.08[±1.7] | 6.81[±0.46] | 40.01[±1.07] | 1374.69[±22.18] |  |

**Table C. Costs avoided due to a 1 µg/m3 by disease and cost parameter (PM2.5) (£million)**

|  |  | Asthma | COPD | Diabetes | CHD | Lung Cancer | Stroke | Total |
| --- | --- | --- | --- | --- | --- | --- | --- | --- |
| Primary care | 2017 | 0.03[±0.16] | 0.72[±0.64] | 0.66[±2.49] | 0.13[±0.2] | 0.01[±0.05] | 0.05[±0.09] | 1.59[±2.59] |
|  | 2017-2025 | 0.98[±0.52] | 20.82[±2.15] | 27.28[±8.3] | 1[±0.68] | 0.03[±0.16] | 0.16[±0.31] | 50.27[±8.63] |
|  | 2017-2035 | 2.95[±0.86] | 61.54[±3.59] | 100.84[±13.82] | 1.54[±1.14] | 0.03[±0.27] | 0.26[±0.52] | 167.16[±14.36] |
| Hospitalisation | 2017 | 0.04[±0.2] | 1.06[±0.94] | 0.94[±3.57] | 2.55[±4.15] | 0.06[±0.41] | 0.9[±1.8] | 5.56[±5.86] |
|  | 2017-2025 | 1.24[±0.66] | 30.55[±3.16] | 39.05[±11.88] | 104.57[±13.82] | 1.94[±1.41] | 19.27[±6.06] | 196.62[±19.53] |
|  | 2017-2035 | 3.75[±1.09] | 90.29[±5.27] | 144.34[±19.78] | 378.26[±23.21] | 3.68[±2.4] | 63.4[±10.25] | 683.71[±32.71] |
| Medication | 2017 | 0.13[±0.66] | 0.23[±0.2] | 0.49[±1.84] | 1.43[±2.33] | 0[±0.03] | 0.63[±1.26] | 2.91[±3.29] |
|  | 2017-2025 | 4.02[±2.16] | 6.59[±0.68] | 20.14[±6.13] | 58.61[±7.74] | 0.15[±0.11] | 13.44[±4.23] | 102.95[±10.98] |
|  | 2017-2035 | 12.14[±3.53] | 19.49[±1.14] | 74.46[±10.2] | 212.02[±13.01] | 0.28[±0.18] | 44.22[±7.15] | 362.59[±18.39] |
| Social care | 2017 | 0.0011[±0.0039] | 0.15[±0.14] | 1.06[±4] | 0.19[±0.31] | 0.01[±0.08] | 0.09[±0.19] | 1.51[±4.02] |
|  | 2017-2025 | 0.02[±0.01] | 4.43[±0.46] | 43.77[±13.32] | 7.85[±1.04] | 0.37[±0.27] | 2.03[±0.64] | 58.48[±13.38] |
|  | 2017-2035 | 0.07[±0.02] | 13.11[±0.77] | 161.77[±22.16] | 28.41[±1.74] | 0.7[±0.46] | 6.67[±1.08] | 210.73[±22.28] |

# NO_2_

**Table D** presents the cumulative incidence cases attributable to NO_2_ in England, by disease between 2017 and 2035. By 2035 it was predicted that 1,140,018 [±11800] new cases of disease would be attributable to NO_2_ in England. The highest number of new disease cases attributable to NO_2_ were for diabetes and asthma (573,363 [±7725] and 335,491 [±2575] cases attributable to NO_2_ by 2035 respectively).

**Table D. Cumulative incidence cases attributable to NO2 in England, by disease and total between 2017 and 2035.**

| **Year** | **Asthma** | **Dementia** | **Diabetes** | **Lung cancer** | **Low birthweight** | **Total** |
| --- | --- | --- | --- | --- | --- | --- |
| In 2017 | 18,361  [±556] | 5008  [±1669] | 29,489  [±1669] | 2226  [±556] | 5564  [±556] | 60,648  [±2549] |
| 2017-2025 | 160,185  [±1717] | 42,303  [±5152] | 269,511  [±5152] | 17,734  [±1717] | 49,794  [±1717] | 539,527  [±7870] |
| 2017-2035 | 335,491  [±2575] | 86,617  [±7725] | 573,363  [±7725] | 42,002  [±2575] | 102,545  [±2575] | 1,140,018  [±11800] |

**Table E** presents the costs attributable to NO_2_ by disease for each cost parameter (primary care, secondary, medication, social care). Table F presents the costs avoided by disease and cost parameter (£million) due to each scenario: a 1 µg/m3 reduction in NO~~2~~ and meeting EU limit values for NO_2_ relative to baseline.

**Table E. Attributable costs by disease and cost parameter (NO_2_) (£million)**

|  |  | Asthma (children)† | Asthma (all) | Dementia | Diabetes | Lung Cancer | Total |
| --- | --- | --- | --- | --- | --- | --- | --- |
| Primary care | 2017 | 0.26  [±0.14] | 0.5  [±0.16] | 0.82  [±0.52] | 10.3  7[±2.48] | 0.1 [±0.04] | 11.7  8[±2.54] |
|  | 2017-2025 | 9.49  [±0.45] | 15.9  [±0.52] | 19.97  [±1.76] | 412.4  9[±8.21] | 1.54  [±0.15] | 449.9  [±8.42] |
|  | 2017-2035 | 35.88  [±0.73] | 59.22  [±0.86] | 47.18  [±2.99] | 1503.16  [±13.59] | 3.64  [±0.26] | 1613.2  [±13.95] |
| Secondary care | 2017 | 0.33  [±0.17] | 0.63  [±0.2] | 0.38  [±0.24] | 14.84  [±3.55] | 0.86  [±0.4] | 16.71  [±3.59] |
|  | 2017-2025 | 12.05  [±0.57] | 20.1  9[±0.66] | 9.15  [±0.81] | 590.41  [±11.76] | 13.86  [±1.38] | 633.6  [±11.88] |
|  | 2017-2035 | 45.56  [±0.93] | 75.19  [±1.09] | 21.6  1[±1.37] | 2151.53  [±19.45] | 32.8  2[±2.35] | 2281.15  [±19.67] |
| Medication | 2017 | 1.08  [±0.56] | 2.04  [±0.66] | 0.59  [±0.38] | 7.6  5[±1.83] | 0.07  [±0.03] | 10.36  [±1.98] |
|  | 2017-2025 | 39.05  [±1.85] | 65.42  [±2.15] | 14.39  [±1.27] | 304.56  [±6.06] | 1.04  [±0.1] | 385.41  [±6.56] |
|  | 2017-2035 | 147.65  [±3.02] | 243.69  [±3.52] | 33.99  [±2.16] | 1109.86  [±10.03] | 2.4  7[±0.18] | 1390  [±10.85] |
| Social care | 2017 | 0.01  [±0] | 0.01  [±0] | 23.6  1[±15.01] | 16.63  [±3.98] | 0.17  [±0.08] | 40.41  [±15.53] |
|  | 2017-2025 | 0.22  [±0.01] | 0.37  [±0.01] | 572.77  [±50.53] | 661.7  [±13.18] | 2.6  5[±0.26] | 1237.49  [±52.22] |
|  | 2017-2035 | 0.84  [±0.02] | 1.39  [±0.02] | 1353.08  [±85.86] | 2411.31  [±21.8] | 6.29  [±0.45] | 3772.07  [±88.59] |

**†**this column does not contribute to the totals since these costs are also contained within the ‘Asthma (All)’ column.

**Table F. Costs avoided by disease and cost parameter (£million) due to each scenario: a 1 µg/m^3^ reduction in NO_2_ and meeting EU limit values for NO_2_ relative to baseline.**

|  |  | Year | Asthma | Dementia | Diabetes | Lung Cancer | Total |
| --- | --- | --- | --- | --- | --- | --- | --- |
| Scenario: 1 µg/m3 reduction | Primary care | 2017 | 0.02[±0.16] | 0.03[±0.52] | 0.6[±2.49] | 0.01[±0.05] | 0.66[±2.55] |
|  |  | 2017-2025 | 0.42[±0.53] | 0.63[±1.89] | 18.34[±8.26] | 0.08[±0.15] | 19.36[±8.46] |
|  |  | 2017-2035 | 1.38[±0.86] | 1.55[±3.01] | 60.97[±13.73] | 0.18[±0.26] | 69.38[±14.65] |
|  | Secondary care | 2017 | 0.02[±0.2] | 0.01[±0.24] | 0.86[±3.56] | 0.06[±0.41] | 0.96[±3.6] |
|  |  | 2017-2025 | 0.53[±0.67] | 0.24[±0.81] | 26.24[±11.82] | 0.68[±1.4] | 27.69[±11.95] |
|  |  | 2017-2035 | 1.75[±1.09] | 0.71[±1.38] | 87.27[±19.65] | 1.66[±2.38] | 91.39[±19.87] |
|  | Medication | 2017 | 0.07[±0.66] | 0.02[±0.38] | 0.44[±1.84] | 0.01[±0.03] | 0.54[±1.99] |
|  |  | 2017-2025 | 1.71[±2.16] | 0.38[±1.27] | 13.54[±6.1] | 0.05[±0.1] | 15.69[±6.59] |
|  |  | 2017-2035 | 5.68[±3.54] | 1.11[±2.17] | 45.02[±10.14] | 0.13[±0.18] | 51.94[±10.96] |
|  | Social care | 2017 | 0[±0] | 0.89[±15.03] | 0.97[±3.99] | 0.01[±0.08] | 1.87[±15.55] |
|  |  | 2017-2025 | 0.01[±0.01] | 15.25[±50.73] | 29.41[±13.25] | 0.13[±0.27] | 44.81[±52.43] |
|  |  | 2017-2035 | 0.03[±0.02] | 44.39[±86.23] | 97.81[±22.02] | 0.32[±0.45] | 142.55[±89] |
|  |  | Year | Asthma | Dementia | Diabetes | Lung Cancer | Total |
| Scenario: EU limit values met | Primary care | 2017 | 0.09[±0.16] | 0.07[±0.52] | 2.11[±2.49] | 0.01[±0.05] | 2.27[±2.55] |
|  |  | 2017-2025 | 3.06[±0.52] | 1.66[±1.77] | 76.6[±8.25] | 0.21[±0.15] | 81.53[±8.46] |
|  |  | 2017-2035 | 12.17[±0.86] | 5.17[±3.01] | 301.03[±13.71] | 0.55[±0.26] | 318.93[±14.06] |
|  | Secondary care | 2017 | 0.11[±0.2] | 0.03[±0.24] | 3.02[±3.56] | 0.06[±0.41] | 3.22[±3.59] |
|  |  | 2017-2025 | 3.89[±0.67] | 0.76[±0.81] | 109.63[±11.81] | 1.85[±1.39] | 116.14[±11.94] |
|  |  | 2017-2035 | 15.46[±1.09] | 2.37[±1.38] | 430.87[±19.62] | 4.99[±2.37] | 453.68[±19.84] |
|  | Medication | 2017 | 0.36[±0.66] | 0.05[±0.38] | 1.56[±1.84] | 0.01[±0.03] | 1.97[±1.99] |
|  |  | 2017-2025 | 12.61[±2.16] | 1.2[±1.27] | 56.55[±6.09] | 0.14[±0.1] | 70.51[±6.59] |
|  |  | 2017-2035 | 50.09[±3.54] | 3.73[±2.17] | 222.26[±10.12] | 0.38[±0.18] | 276.46[±10.94] |
|  | Social care | 2017 | 0[±0] | 1.91[±15.03] | 3.38[±3.99] | 0.01[±0.08] | 5.31[±15.55] |
|  |  | 2017-2025 | 0.07[±0.01] | 47.72[±50.72] | 122.87[±13.24] | 0.36[±0.27] | 171.02[±52.42] |
|  |  | 2017-2035 | 0.29[±0.02] | 148.39[±86.2] | 482.89[±21.99] | 0.95[±0.45] | 632.52[±88.96] |

3. COMBINING ATTRIBUTABLE COSTS FOR NO_2_ and PM_2.5_ WHERE EVIDENCE IS MOST ROBUST

**Table G. Attributable costs for NO_2_ and and PM_2.5_ where evidence is most robust (£millions)**

|  | Year | PM_2.5_† | NO_2_* | Total |  |
| --- | --- | --- | --- | --- | --- |
| Primary care | 2017 | 1.419[±0.278] | 0.26[±0.14] | 1.679[±0.31] |  |
|  | 2017-2025 | 14.636[±0.914] | 9.49[±0.45] | 24.126[±1.02] |  |
|  | 2017-2035 | 35.802[±1.515] | 35.88[±0.73] | 71.682[±1.68] |  |
| Hospitalisation | 2017 | 23.744[±4.531] | 0.33[±0.17] | 24.074[±4.53] |  |
|  | 2017-2025 | 909.601[±14.963] | 12.05[±0.57] | 921.651[±14.97] |  |
|  | 2017-2035 | 3043.282[±24.943] | 45.56[±0.93] | 3088.842[±24.96] |  |
| Medication | 2017 | 14.092[±2.716] | 1.08[±0.56] | 15.172[±2.77] |  |
|  | 2017-2025 | 540.802[±8.972] | 39.05[±1.85] | 579.852[±9.16] |  |
|  | 2017-2035 | 1821.976[±14.951] | 147.65[±3.02] | 1969.626[±15.25] |  |
| Social care | 2017 | 1.942[±0.371] | 0.01[±0] | 1.952[±0.37] |  |
|  | 2017-2025 | 73.175[±1.233] | 0.22[±0.01] | 73.395[±1.23] |  |
|  | 2017-2035 | 242.456[±2.059] | 0.84[±0.02] | 243.296[±2.06] |  |
| TOTALS | 2017 | 41.197[±5.303] | 1.68[±0.602] | 42.877[±5.34] |  |
|  | 2017-2025 | 1538.214[±17.514] | 60.81[±1.987] | 1599.024[±17.63] |  |
|  | 2017-2035 | 5143.516[±29.193] | 229.93[±3.243] | 5373.446[±29.37] |  |

†Includes coronary heart disease (CHD), childhood asthma, stroke, lung cancer

*Includes childhood asthma
